# Global burden of thyroid cancer in adolescents and young adults (aged 15–39 years) from 1990 to 2021: A systematic analysis of the Global Burden of Disease Study 2021

**DOI:** 10.1371/journal.pone.0318605

**Published:** 2025-02-14

**Authors:** Zijian Qiu, Shengjian Yu, Lin Zheng, Ying Lou, Xiuxia Chen, Feng Xuan

**Affiliations:** 1 Department of Radiation Oncology, The Quzhou Affiliated Hospital of Wenzhou Medical University, Quzhou People’s Hospital, Quzhou, China; 2 Department of Radiation Oncology, Zhuji Affiliated Hospital of Wenzhou Medical University, Shaoxing, China; 3 Department of Radiation Oncology, Taizhou Cancer Hospital, Wenling, China; 4 Department of Medical Oncology, Zhuji Affiliated Hospital of Wenzhou Medical University, Shaoxing, China; 5 Department of Pathology, Zhuji Affiliated Hospital of Wenzhou Medical University, Shaoxing, China; Debre Tabor University, ETHIOPIA

## Abstract

**Background:**

Thyroid cancer (TC) is the most common malignancy of the endocrine system and head-and-neck region, yet data on its burden in adolescents and young adults (AYAs) is lacking. This study aimed to estimate the global burden of TC among AYAs from 1990 to 2021.

**Methods:**

Utilizing the Global Burden of Disease (GBD) 2021 data, we analyzed age-standardized rates of incidence, prevalence, and disability-adjusted life-years (DALYs) on global, regional, and national scales. Joinpoint regression was employed to determine average annual percentage change (AAPC), with frontier analysis revealing regions for improvement. Decomposition analysis assessed the impacts of population aging, growth, and epidemiological changes. Projections for disease burden extending to 2040 were generated using the Bayesian Age-Period-Cohort model.

**Result:**

In 2021, there were 48.2 thousand incident cases, 436.1 thousand prevalent cases, and 183.5 thousand DALYs worldwide. Meantime, the age-standardized incidence rates (ASIR), age-standardized prevalence rates (ASPR), and age-standardized DALYs rates (ASDR) were 1.6, 14.3 and 6.1 per 100 000, respectively. From 1990 to 2021, the ASIR, ASPR and ASDR increased with AAPCs of 1.73, 1.77, and 0.38, respectively. Socio-demographic resources in Saudi Arabia, Taiwan (Province of China), Iceland, United Arab Emirates, and United States Virgin Islands have the potential to lower ASDR due to TC among AYAs. Furthermore, 13.3 thousand and 34.9 thousand new cases occurred in the males and females in 2021. Among 5 age groups, the highest numbers of incidence, prevalence, and DALYs, along with ASRs, were observed in the 35–39 age group. Global projections indicated a continuous rise in numbers of incidence, prevalence, and DALYs, with estimates of 60.2 thousand, 558.4 thousand, and 199.7 thousand by 2040, respectively.

**Conclusion:**

The global burden of TC among AYAs was on the rise, with significant disparities by regions, genders, and age groups, highlighting the necessity for targeted and effective interventions.

## Introduction

Thyroid cancer (TC), a prominent endocrine malignancy, has emerged as a significant global health concern, ranking seventh in cancer incidence worldwide as of 2022 [1]. While TC generally has a favorable prognosis, it can still significantly impact the quality of life, especially in adolescents and young adults (AYAs) aged 15–39 years [2–4]. Beyond the physical health challenges, young patients aged 15–39 years often face additional issues such as psychological distress and concerns about fertility. The diagnosis and treatment of cancer can lead to anxiety, depression, and even other mental health issues such as suicide [5], posing unique challenges for adolescents and young adults (AYAs) during their critical developmental phases. Moreover, cancer treatment can potentially affect reproductive health, raising concerns about future fertility [6,7]. Addressing these specific challenges in AYAs is crucial for providing comprehensive care. Existing studies have predominantly focused on TC across all ages, neglecting this specific demographic.

To address this research gap, our study leveraged data from the Global Burden of Disease (GBD) 2021 to analyze trends of TC among AYAs. We aimed to deliver a comprehensive analysis of incidence, prevalence, and disability-adjusted life-years (DALYs) for TC among AYAs from 1990 to 2021. Using methods such as Joinpoint regression and Bayesian age-period-cohort (BAPC) prediction model, we evaluated temporal changes in the disease burden of thyroid cancer among AYAs, and the future disease burden. By focusing on the 15–39 age group, our findings offered essential insights for policymakers to improve prevention and treatment strategies and to better allocate resources for TC management in AYAs.

## Methods

### Study population and data collection

The Global Burden of Disease Study 2021, spearheaded by the Institute for Health Metrics and Evaluation (IHME) at the University of Washington, stands as a preeminent dataset in global health research. The dataset provides a comprehensive record of detailed statistics for 371 diseases and injuries and 88 health risk factors across 204 countries and territories from 1990 to 2021 [8,9]. The specific methodologies for the estimation of disease burden and the analysis of risk factors were thoroughly detailed in pertinent scholarly literature [8–11]. In this study, our comprehensive secondary analysis of TC utilized the GBD results tool on the Global Health Data Exchange platform (https://vizhub.healthdata.org/gbd-results/), covering global, Social Demographic Index regions, GBD regions, and 204 countries/territories. Disease burden was measured across three dimensions: incidence, prevalence, and DALYs, with results presented through both numbers and rates. Our study also specifically differentiated by gender, including data for males, females, and an overall perspective, ensuring a thorough and in-depth analysis. Furthermore, this study concentrated on AYAs as a critical demographic, delineating this group as those individuals who were aged between 15 and 39 years. In our study, TC diagnoses were identified using the following International Classification of Diseases (ICD) codes: ICD-9 codes 193–193.9 and 226–226.9, and ICD10 codes C73-C73.9, D09.3, D09.8, D34-D34.9, and D44.0 [8,10].

The analysis presented here strictly adheres to the Guidelines for Accurate and Transparent Health Assessment Reporting (GATHER) [12], ensuring the scientific rigor and reliability of the study findings (S1 Table in S1 File).

### Sociodemographic index (SDI)

The Social Demographic Index (SDI) serves as a pivotal composite indicator for assessing the level of socio-economic development of a country/territory [10]. It is constructed from three principal components: the total fertility rate of the population aged under 25, the average years of education among individuals aged 15 and above, and the lagging indicator of per capita income. The SDI scale ranges from 0 to 1, where 0 indicates the lowest level of development characterized by the fewest years of education, the lowest per capita income, and the highest fertility rate; conversely, 1 denotes the highest level of development. Countries or territories are stratified into five developmental quintiles based on their SDI values: Low (0–0.465816), Low-middle (0.465816–0.618829), Middle (0.618829–0.711975), High-middle (0.711975–0.81030), and High (0.81030–1). For a detailed distribution of locations and their corresponding SDI quintiles, refer to S2–4 Tables in S1 File.

### Ethical approval and consent to participate

This research adhered to the principles of the Declaration of Helsinki and complied with applicable local laws. As the study utilized publicly available data, it was exempted from ethical review by the institutional ethics committee.

### Statistical analysis

#### Age-standardized rate (ASR).

Demographic variations in population age structure can lead to significant heterogeneity in the burden of TC. To ensure the comparability of statistical indicators, we applied age-specific weights to the crude rates based on the age distribution and calculated the age-standardized rate (ASR) using the following formula [13,14]. Subsequently, the age-standardized incidence rate (ASIR), age-standardized prevalence rate (ASPR), and age-standardized DALYs rate (ASDR) were utilized to gauge the burden of thyroid cancer.


$$ASR=\frac{\sum_{i=1}^{N}\alpha_{i}W_{i}}{\sum_{i=1}^{N}W_{i}}\times 100,000$$

where *N* represents the total number of age groups, with *i* indicating the *i*th age group. *ɑ*_*i*_ denotes the age-specific rate for that group, and *W_i_ *refers to the number of individuals in the standard population for the corresponding age group.

#### Joinpoint regression.

Our study employed the Joinpoint Regression software (version 5.1.0.0) from the National Cancer Institute (NCI) to perform trend analysis on age-standardized rates (ASRs), allowing for up to five joinpoints [15]. Through this method, we calculated the annual percentage change (APC) and the average annual percentage change (AAPC), then employed the Monte Carlo permutation method to verify the statistical significance of the APC for each trend segment and the overall AAPC value. The joinpoint regression model enabled us to quantify the temporal trends in ASIR, ASPR, and ASDR across different historical periods and genders in global, SDI regions, GBD regions and 204 countries/territories from 1990 to 2021. When the estimated value of APC or AAPC and the lower limit of its 95% confidence interval (CI) exceed 0, the trend was defined as “increasing”. Conversely, if the estimated value of APC or AAPC and the upper limit of the 95% CI are below 0, it indicated a “decreasing” trend. If the 95% CI includes 0, the disease burden was considered to be “relatively stable” statistically.

#### Decomposition analysis.

In this study, we conducted a decomposition analysis method [16,17], combining demographic and epidemiological data, to assess and quantify the specific contributions of population aging, growth, and epidemiologic changes to the burden of TC among individuals aged 15 to 39 years between 1990 and 2021.

#### Frontier analysis.

Frontier analysis was applied to examine the association between thyroid cancer ASDR and socio-demographic development, identifying the lowest achievable ASDR based on SDI [18,19]. A non-linear frontier was constructed using Data Envelopment Analysis (DEA) with the free disposal hull (FDH) approach, delineating the minimum achievable ASDR for each SDI level. By incorporating 100 bootstrapped samples and employing LOESS regression for smoothing, we ensured robustness. Super-efficient countries or territories were excluded to avoid distortion. The effective difference, defined as the distance from the frontier, provided insights into unrealized opportunities for burden reduction, offering a framework to prioritize interventions in regions lagging behind optimal performance.

#### Spearman’s rank correlation analysis.

In this study, we employed a smooth spline regression model to assess the correlation between the disease burden of TC and SDI across 21 GBD regions and 204 countries/territories. To quantify the strength and statistical significance of this association, we conducted Spearman’s rank correlation analysis to calculate the R indices and the corresponding p-values.

#### Bayesian age-period-cohort model.

Our study utilized the Bayesian Age-Period-Cohort (BAPC) model to assess and forecast the numbers and rates of incidence, prevalence, and DALYs up to the year 2040 [20,21]. The BAPC model, augmented with the Integrated Nested Laplacian Approximation (INLA), is an efficient Bayesian statistical method that precisely differentiates the impacts of age, period, and cohort on health outcomes. The INLA technique enhances computational efficiency, convergence, and robustness, and facilitates model comparison using the Deviance Information Criterion (DIC). The R statistical software, with BAPC (version 0.0.36) and INLA (version 23.04.24) packages, were used for BAPC analyses.

All statistical analyses were meticulously executed via R software (version 4.4.1) and the Joinpoint Regression Program [15] (version 5.1.0.0). P-value <  0.05 defined significance.

## Results

### Global level

In 2021, the global incidence of thyroid cancer in AYAs reached an estimated 48.2 thousand (95% uncertainty interval [UI] 40.8–58.4) cases, with an ASIR of 1.6 (95% CI 1.3–1.9) per 100,000 (Table 1). The prevalence was 436.1 thousand (95% UI 369.0–528.0) cases, and the disease burden in DALYs was 183.5 thousand (95% UI 149.9–232.3) cases, corresponding to an ASPR of 14.3 (95% CI 12.1–17.4) per 100,000 and an ASDR of 6.1 (95% CI 5.0–7.7) per 100,000, respectively (Tables 2 and 3).

**Table 1:** Numbers and age-standardized rates of incidence in 1990 and 2021, and their AAPCs from 1990 to 2021 at the global and regional levels.

	Numbers (thousands), 1990	Age-standardized rate (per 100 000), 1990	Numbers (thousands), 2021	Age-standardized rate (per 100 000), 2021	Total percentage change in number, 1990-2021	AAPC of age-standardized rate, 1990-2021	p value
Global	19.3 (17.2–22.1)	0.9 (0.8–1.1)	48.2 (40.8–58.4)	1.6 (1.3–1.9)	1.5 (1.2 to 2.0)	1.73 (1.60 to 1.87)	<0.001
Sex							
Male	4.7 (4.3–5.2)	0.4 (0.4–0.5)	13.3 (11.1–15.1)	0.9 (0.7–1.0)	1.8 (1.4 to 2.1)	2.15 (2.02 to 2.29)	<0.001
Female	14.6 (12.6–17.2)	1.4 (1.2–1.7)	34.9 (28.2–44.9)	2.3 (1.9–3.0)	1.4 (1.0 to 2.0)	1.56 (1.38 to 1.74)	<0.001
Sociodemographic index							
High SDI	5.7 (5.3–6.1)	1.6 (1.5–1.7)	8.5 (7.7–9.7)	2.1 (2.0–2.4)	0.5 (0.4 to 0.6)	1.16 (0.78 to 1.55)	<0.001
High-middle SDI	4.5 (3.9–5.0)	1.0 (0.9–1.1)	7.9 (6.8–9.6)	1.6 (1.3–1.9)	0.8 (0.5 to 1.2)	1.42 (1.16 to 1.68)	<0.001
Middle SDI	5.0 (4.2–6.0)	0.7 (0.6–0.9)	15.8 (12.6–19.0)	1.6 (1.3–2.0)	2.2 (1.7 to 2.8)	2.68 (2.53 to 2.83)	<0.001
Low-middle SDI	2.9 (2.3–3.9)	0.7 (0.5–0.9)	10.7 (8.1–15.2)	1.4 (1.0–1.9)	2.7 (1.9 to 4.3)	2.24 (1.93 to 2.55)	<0.001
Low SDI	1.3 (1.0–1.7)	0.8 (0.6–1.0)	5.2 (3.8–7.9)	1.3 (0.9–1.9)	3.0 (2.0 to 4.9)	1.64 (1.37 to 1.91)	<0.001
Region							
High-income Asia Pacific	0.9 (0.7–1.1)	1.3 (1.1–1.6)	1.1 (0.9–1.5)	2.0 (1.6–2.6)	0.3 (0.1 to 0.6)	1.44 (1.02 to 1.86)	<0.001
High-income North America	1.8 (1.7–1.9)	1.5 (1.4–1.6)	2.9 (2.7–3.1)	2.2 (2.1–2.4)	0.6 (0.5 to 0.7)	1.51 (0.48 to 2.56)	0.004
Western Europe	2.6 (2.3–2.9)	1.7 (1.6–2.0)	2.4 (2.1–2.8)	1.7 (1.4–1.9)	-0.1 (-0.2 to 0.1)	-0.16 (-0.32 to 0.01)	0.06
Australasia	0.1 (0.1–0.2)	1.4 (1.1–1.9)	0.2 (0.2–0.3)	2.0 (1.4–2.7)	0.9 (0.3 to 1.6)	1.24 (-0.16 to 2.65)	0.08
Andean Latin America	0.1 (0.1–0.1)	0.7 (0.5–1.0)	0.4 (0.3–0.6)	1.5 (1.0–2.1)	3.1 (1.9 to 4.6)	2.85 (2.49 to 3.20)	<0.001
Tropical Latin America	0.3 (0.2–0.3)	0.4 (0.4–0.5)	0.7 (0.6–0.8)	0.8 (0.7–0.9)	1.7 (1.4 to 2.0)	1.69 (1.47 to 1.90)	<0.001
Central Latin America	0.4 (0.4–0.4)	0.7 (0.6–0.7)	1.2 (1.0–1.4)	1.2 (1.0–1.4)	2.0 (1.6 to 2.5)	2.02 (1.53 to 2.50)	<0.001
Southern Latin America	0.2 (0.1–0.2)	0.9 (0.7–1.1)	0.3 (0.2–0.4)	1.2 (0.9–1.5)	0.9 (0.5 to 1.4)	0.89 (0.08 to 1.71)	0.03
Caribbean	0.1 (0.1–0.1)	0.7 (0.6–0.9)	0.2 (0.1–0.2)	1.0 (0.8–1.2)	0.8 (0.5 to 1.2)	0.82 (0.05 to 1.60)	0.04
Central Europe	0.7 (0.6–0.8)	1.4 (1.2–1.5)	0.4 (0.4–0.5)	1.0 (0.9–1.2)	-0.4 (-0.5 to -0.3)	-0.89 (-1.78 to 0.01)	0.053
Eastern Europe	1.1 (1.0–1.2)	1.2 (1.1–1.3)	1.3 (1.1–1.4)	1.6 (1.4–1.8)	0.2 (0.0 to 0.3)	0.86 (-0.48 to 2.21)	0.21
Central Asia	0.2 (0.2–0.3)	0.8 (0.7–1.0)	0.3 (0.2–0.3)	0.7 (0.6–0.9)	0.3 (0.0 to 0.5)	-0.57 (-2.04 to 0.93)	0.45
North Africa and Middle East	1.3 (1.0–1.7)	1.1 (0.8–1.5)	5.9 (4.5–7.5)	2.2 (1.7–2.8)	3.7 (2.4 to 5.3)	2.51 (2.36 to 2.65)	<0.001
South Asia	3.2 (2.5–4.4)	0.8 (0.6–1.1)	13.9 (10.5–19.4)	1.8 (1.3–2.5)	3.3 (2.2 to 5)	2.58 (2.19 to 2.97)	<0.001
Southeast Asia	1.9 (1.3–2.4)	1.0 (0.7–1.3)	5.3 (3.8–7.1)	1.9 (1.3–2.5)	1.8 (1.3 to 2.5)	1.91 (1.73 to 2.09)	<0.001
East Asia	3.5 (2.7–4.4)	0.7 (0.5–0.8)	8.0 (6.4–10.5)	1.4 (1.1–1.9)	1.3 (0.7 to 2.3)	2.61 (2.36 to 2.87)	<0.001
Oceania	0.0 (0.0–0.0)	0.5 (0.3–0.7)	0.0 (0.0–0.0)	0.6 (0.3–0.9)	1.8 (1.1 to 2.8)	0.60 (0.27 to 0.93)	<0.001
Western Sub-Saharan Africa	0.1 (0.1–0.1)	0.1 (0.1–0.2)	0.3 (0.2–0.5)	0.2 (0.1–0.3)	2.7 (1.7 to 4)	1.10 (0.99 to 1.21)	<0.001
Eastern Sub-Saharan Africa	0.8 (0.5–1.0)	1.2 (0.8–1.6)	2.9 (2–4.9)	1.8 (1.2–3.0)	2.8 (1.6 to 5.2)	1.39 (1.32 to 1.47)	<0.001
Central Sub-Saharan Africa	0.0 (0.0–0.1)	0.2 (0.1–0.3)	0.1 (0.1–0.2)	0.2 (0.1–0.4)	2.6 (1.4 to 4.4)	0.96 (0.79 to 1.13)	<0.001
Southern Sub-Saharan Africa	0.1 (0.1–0.2)	0.6 (0.5–0.8)	0.3 (0.2–0.4)	0.8 (0.6–1.1)	1.3 (0.8 to 2.0)	0.75 (-0.03 to 1.53)	0.06

Data in parentheses are 95% uncertainty intervals for numbers, total percentage change, and 95% CIs for age-standardized rates, and AAPCs. AAPC=average annual percentage change.

**Table 2:** Numbers and age-standardized rates of prevalence in 1990 and 2021, and their AAPCs from 1990 to 2021 at the global and regional levels.

	Numbers (thousands), 1990	Age-standardized rate (per 100 000), 1990	Numbers (thousands), 2021	Age-standardized rate (per 100 000), 2021	Total percentage change in number, 1990-2021	AAPC of age-standardized rate, 1990-2021	p value
Global	172.3 (154.3–197)	8.3 (7.4–9.5)	436.1 (369.0–528.0)	14.3 (12.1–17.4)	1.5 (1.2 to 2.0)	1.77 (1.64 to 1.91)	<0.001
Sex							
Male	41.3 (37.8–45.5)	3.9 (3.6–4.3)	119 (99.3–135.8)	7.7 (6.4–8.8)	1.9 (1.4 to 2.2)	2.22 (2.08 to 2.35)	<0.001
Female	131.0 (113.0–154.5)	12.8 (11.0–15.0)	317.1 (255.8–407.4)	21.1 (17.0–27.2)	1.4 (1.0 to 2.1)	1.59 (1.42 to 1.77)	<0.001
Sociodemographic index							
High SDI	51.5 (48.1–55.2)	14.2 (13.2–15.2)	77.7 (70.8–88.3)	19.7 (17.9–22.3)	0.5 (0.4 to 0.6)	1.18 (0.80 to 1.56)	<0.001
High−middle SDI	40.2 (35.4–45.2)	9.0 (7.9–10.1)	71.8 (61.6–87.3)	14.3 (12.2–17.4)	0.8 (0.5 to 1.2)	1.46 (1.20 to 1.71)	<0.001
Middle SDI	44.3 (37.0–53.0)	6.4 (5.3–7.6)	143.8 (114.1–173)	14.7 (11.7–17.7)	2.2 (1.7 to 2.9)	2.75 (2.59 to 2.90)	<0.001
Low−middle SDI	25.1 (19.7–34.1)	5.9 (4.7–8.0)	96.2 (72.8–136.2)	12.3 (9.3–17.3)	2.8 (2.0 to 4.5)	2.33 (2.02 to 2.64)	<0.001
Low SDI	11.0 (8.1–14.7)	6.5 (4.8–8.6)	46.3 (33.6–70.0)	11.2 (8.2–16.9)	3.2 (2.1 to 5.2)	1.78 (1.50 to 2.06)	<0.001
Region							
High−income Asia Pacific	8.1 (6.5–10.1)	11.9 (9.6–14.9)	10.4 (8.3–13.6)	18.3 (14.6–24)	0.3 (0.1 to 0.6)	1.45 (1.03 to 1.88)	<0.001
High−income North America	16.2 (15.1–17.3)	13.3 (12.4–14.2)	26.5 (24.7–28.4)	20.3 (19–21.8)	0.6 (0.5 to 0.7)	1.52 (0.48 to 2.57)	0.004
Western Europe	23.4 (20.9–26.3)	15.9 (14.2–17.8)	21.8 (19.0–25.3)	15.2 (13.2–17.6)	−0.1 (−0.2 to 0.1)	−0.14 (−0.3 to 0.03)	0.10
Australasia	1.1 (0.8–1.5)	13.2 (9.6–17.6)	2.1 (1.5–2.9)	18.0 (12.5–25.1)	0.9 (0.3 to 1.6)	1.25 (−0.14 to 2.67)	0.08
Andean Latin America	0.8 (0.6–1.2)	6.1 (4.4–8.4)	3.5 (2.4–5.1)	13.2 (9.0–18.9)	3.2 (2.0 to 4.8)	2.95 (2.60 to 3.31)	<0.001
Tropical Latin America	2.4 (2.1–2.7)	4.0 (3.5–4.5)	6.5 (5.8–7.3)	6.9 (6.2–7.8)	1.7 (1.5 to 2.0)	1.74 (1.52 to 1.96)	<0.001
Central Latin America	3.5 (3.1–4.0)	5.8 (5.2–6.6)	10.9 (9.3–12.7)	10.8 (9.2–12.6)	2.1 (1.6 to 2.5)	2.08 (1.60 to 2.56)	<0.001
Southern Latin America	1.5 (1.1–1.8)	7.9 (6.2–9.9)	2.8 (2.2–3.5)	10.5 (8.3–13.3)	0.9 (0.6 to 1.4)	0.93 (0.11 to 1.75)	0.03
Caribbean	0.9 (0.7–1.1)	6.6 (5.5–8.0)	1.6 (1.3–2.0)	8.8 (6.9–11.0)	0.8 (0.5 to 1.2)	0.85 (0.05 to 1.65)	0.04
Central Europe	6.2 (5.5–7.0)	12.4 (10.9–14.0)	3.9 (3.3–4.5)	9.6 (8.1–11.1)	−0.4 (−0.5 to −0.3)	−0.85 (−1.74 to 0.05)	0.06
Eastern Europe	9.7 (9.0–10.7)	10.5 (9.7–11.6)	11.5 (10.1–13.1)	14.2 (12.5–16.2)	0.2 (0.0–0.3)	0.86 (−0.46 to 2.20)	0.20
Central Asia	2.0 (1.7–2.3)	7.5 (6.5–8.6)	2.6 (2.2–3.1)	6.5 (5.4–7.8)	0.3 (0.1 to 0.5)	−0.54 (−2.00 to 0.95)	0.48
North Africa and Middle East	11.4 (8.7–15.8)	9.5 (7.3–13.1)	54.1 (41.0–68.3)	20.5 (15.6–25.9)	3.7 (2.5 to 5.4)	2.53 (2.39 to 2.67)	<0.001
South Asia	28.2 (21.7–38.6)	6.9 (5.3–9.4)	124.1 (94.2–174.6)	15.9 (12.1–22.3)	3.4 (2.3 to 5.2)	2.69 (2.30 to 3.08)	<0.001
Southeast Asia	16.7 (11.4–21.4)	9.2 (6.3–11.7)	48.0 (34.1–64.9)	16.9 (12–22.9)	1.9 (1.3 to 2.6)	1.97 (1.74 to 2.20)	<0.001
East Asia	31.4 (24.1–39.2)	5.9 (4.5–7.4)	73.3 (58.1–96.1)	13.2 (10.5–17.3)	1.3 (0.7 to 2.4)	2.68 (2.43 to 2.93)	<0.001
Oceania	0.1 (0.1–0.1)	4.1 (2.4–6.2)	0.3 (0.2–0.4)	5.1 (2.9–8.2)	1.8 (1.1 to 2.8)	0.63 (0.30 to 0.97)	<0.001
Western Sub−Saharan Africa	0.8 (0.5–1.2)	1.2 (0.9–1.8)	3.0 (2.0–4.7)	1.8 (1.2–2.8)	2.8 (1.7 to 4.1)	1.18 (1.08 to 1.28)	<0.001
Eastern Sub−Saharan Africa	6.5 (4.6–8.9)	10.0 (7.2–13.7)	25.9 (17.5–43.4)	16.1 (10.8–26.8)	3.0 (1.7 to 5.5)	1.53 (1.42 to 1.64)	<0.001
Central Sub−Saharan Africa	0.3 (0.1–0.5)	1.4 (0.8–2.5)	1.0 (0.5–1.8)	2.0 (1.0–3.7)	2.7 (1.4 to 4.6)	1.04 (0.9 to 1.19)	<0.001
Southern Sub−Saharan Africa	1.1 (0.8–1.4)	5.5 (4.3–7.2)	2.4 (1.7–3.4)	6.8 (4.9–9.8)	1.2 (0.7 to 2.0)	0.75 (−0.04 to 1.55)	0.06

Data in parentheses are 95% uncertainty intervals for numbers, total percentage change, and 95% CIs for age-standardized rates, and AAPCs. AAPC=average annual percentage change.

**Table 3:** Numbers and age-standardized rates of DALYs in 1990 and 2021, and their AAPCs from 1990 to 2021 at the global and regional levels.

	Numbers (thousands), 1990	Age-standardized rate (per 100 000), 1990	Numbers (thousands), 2021	Age-standardized rate (per 100 000), 2021	Total percentage change in number, 1990-2021	AAPC of age-standardized rate, 1990-2021	p value
Global	114.7 (99.2–136.9)	5.4 (4.7–6.4)	183.5 (149.9–232.3)	6.1 (5.0–7.7)	0.6 (0.3 to 1.0)	0.38 (0.25 to 0.51)	<0.001
Sex							
Male	36.9 (32.6–42.8)	3.4 (3.0–4.0)	64.2 (51.1–75.8)	4.2 (3.3–5.0)	0.7 (0.4 to 1.0)	0.64 (0.49 to 0.78)	<0.001
Female	77.8 (62.6–99.1)	7.4 (6–9.4)	119.3 (90.8–165.2)	8 (6.1–11.2)	0.5 (0.2 to 1.1)	0.23 (0.01 to 0.44)	0.04
Sociodemographic index							
High SDI	13.7 (12.6–15.2)	3.8 (3.5–4.2)	12.6 (10.8–15.0)	3.2 (2.8–3.9)	−0.1 (−0.2 to 0.0)	−0.44 (−0.60 to −0.28)	<0.001
High-middle SDI	19.9 (17.3–22.8)	4.4 (3.8–5.1)	16.9 (14.3–20.5)	3.5 (2.9–4.2)	−0.2 (−0.3 to 0.0)	−0.84 (−1.21 to −0.47)	<0.001
Middle SDI	34.1 (29–40.5)	4.8 (4.1–5.7)	51.0 (41.6–60.3)	5.3 (4.3–6.3)	0.5 (0.3 to 0.8)	0.34 (0.18 to 0.49)	<0.001
Low-middle SDI	29.8 (23.6–40.3)	6.9 (5.5–9.3)	63.0 (48.3–87.0)	8.0 (6.1–11.0)	1.1 (0.6 to 2.0)	0.47 (0.22 to 0.72)	<0.001
Low SDI	17.1 (12.9–22.1)	9.8 (7.4–12.6)	39.9 (29.0–59.1)	9.4 (6.9–13.9)	1.3 (0.7 to 2.4)	−0.14 (−0.38 to 0.10)	0.26
Region							
High-income Asia Pacific	1.9 (1.6–2.4)	2.8 (2.3–3.5)	1.3 (1.0–1.8)	2.4 (1.8–3.2)	−0.3 (−0.4 to −0.2)	−0.46 (−0.72 to −0.19)	<0.001
High-income North America	3.2 (2.9–3.7)	2.7 (2.4–3.0)	4.2 (3.6–4.8)	3.2 (2.8–3.7)	0.3 (0.2 to 0.4)	0.59 (−0.23 to 1.40)	0.16
Western Europe	6.5 (5.9–7.3)	4.4 (4.0–5.0)	3.4 (2.9–4.1)	2.4 (2.1–2.9)	−0.5 (−0.5 to −0.4)	−2.01 (−2.21 to −1.81)	<0.001
Australasia	0.3 (0.2–0.4)	3.3 (2.6–4.3)	0.3 (0.2–0.4)	2.8 (2.0–3.9)	0.2 (−0.2 to 0.6)	−0.35 (−1.26 to 0.57)	0.46
Andean Latin America	0.9 (0.7–1.1)	6.2 (4.6–8.0)	1.6 (1.1–2.1)	5.8 (4.2–7.8)	0.8 (0.4 to 1.4)	−0.27 (−1.15 to 0.62)	0.55
Tropical Latin America	2.0 (1.9–2.2)	3.3 (3–3.6)	3.1 (2.8–3.4)	3.4 (3.1–3.7)	0.5 (0.4 to 0.7)	−0.09 (−0.41 to 0.23)	0.58
Central Latin America	3.0 (2.7–3.2)	4.7 (4.3–5.2)	4.7 (4.1–5.4)	4.6 (4.1–5.3)	0.6 (0.4 to 0.8)	−0.10 (−0.55 to 0.34)	0.65
Southern Latin America	0.9 (0.8–1.1)	4.9 (4.1–5.9)	1.0 (0.8–1.2)	3.7 (3.0–4.5)	0.1 (−0.1 to 0.3)	−1.00 (−1.74 to −0.25)	0.01
Caribbean	0.6 (0.5–0.8)	4.6 (3.8–5.7)	0.9 (0.6–1.2)	4.7 (3.5–6.4)	0.4 (0.1 to 0.7)	−0.08 (−0.65 to 0.48)	0.77
Central Europe	2.9 (2.7–3.2)	5.9 (5.5–6.4)	1.0 (0.8–1.1)	2.4 (2.1–2.7)	−0.7 (−0.7 to −0.6)	−3.00 (−3.88 to −2.11)	<0.001
Eastern Europe	3.5 (3.2–3.8)	3.8 (3.5–4.2)	2.6 (2.3–3.0)	3.3 (2.9–3.9)	−0.2 (−0.3 to −0.1)	−0.53 (−1.81 to 0.77)	0.42
Central Asia	1.4 (1.3–1.6)	5.3 (4.8–5.9)	1.2 (1.0–1.4)	3.0 (2.5–3.5)	−0.2 (−0.3 to 0.0)	−1.99 (−3.65 to −0.31)	0.02
North Africa and Middle East	4.9 (3.8–6.9)	4.0 (3.1–5.6)	11.6 (9.0–14.6)	4.4 (3.4–5.6)	1.4 (0.7 to 2.2)	0.37 (0.12 to 0.63)	0.004
South Asia	36.1 (28.3–49.3)	8.6 (6.8–11.8)	81.4 (62.3–111.6)	10.4 (8–14.2)	1.3 (0.7 to 2.1)	0.55 (0.24 to 0.85)	<0.001
Southeast Asia	11.7 (8.2–14.4)	6.3 (4.4–7.8)	17.0 (12.8–21.6)	6.0 (4.5–7.6)	0.5 (0.2 to 0.8)	−0.17 (−0.34 to 0.00)	0.0499
East Asia	21.5 (16.9–27.2)	4.0 (3.1–5.0)	18.1 (14.3–23.4)	3.4 (2.7–4.4)	−0.2 (−0.3 to 0.2)	−0.53 (−0.94 to −0.12)	0.0108
Oceania	0.1 (0.0–0.1)	3.3 (2.0–4.9)	0.2 (0.1–0.3)	3.3 (1.9–5.3)	1.3 (0.7 to 2.0)	0.02 (−0.14 to 0.19)	0.78
Western Sub-Saharan Africa	1.0 (0.7–1.4)	1.6 (1.1–2.2)	2.5 (1.7–3.8)	1.4 (1.0–2.2)	1.4 (0.8 to 2.2)	−0.27 (−0.45 to −0.09)	0.004
Eastern Sub-Saharan Africa	10.9 (8.1–14.6)	16.3 (12.1–22.0)	24.7 (16.8–39.5)	14.9 (10.2–23.8)	1.3 (0.6 to 2.7)	−0.28 (−0.38 to −0.18)	<0.001
Central Sub-Saharan Africa	0.4 (0.3–0.7)	2.2 (1.3–3.6)	1.0 (0.5–1.7)	2.0 (1.1–3.5)	1.4 (0.6 to 2.5)	−0.37 (−0.46 to −0.28)	<0.001
Southern Sub-Saharan Africa	1.0 (0.8–1.2)	4.9 (4.0–6.2)	1.9 (1.3–2.8)	5.5 (3.8–8.0)	1.0 (0.5 to 1.6)	0.41 (−0.18 to 1.01)	0.17

Data in parentheses are 95% uncertainty intervals for numbers, total percentage change, and 95% CIs for age-standardized rates, and AAPCs. DALY=disability-adjusted life-year. AAPC=average annual percentage change.

**Fig 1:**
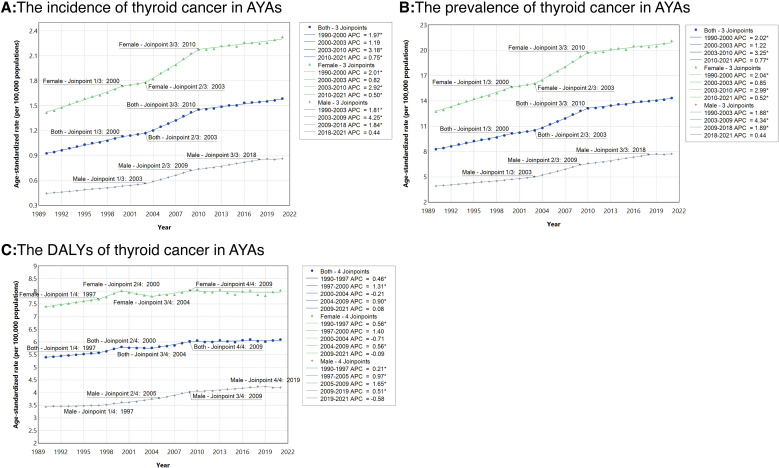
The APCs of age-standardized rates for thyroid cancer among adolescents and young adults at the global level by sex based on the Joinpoint regression analysis model. (A), Age-standardized incidence rates; (B), Age-standardized prevalence rates; (C), Age-standardized DALYs rates. APC=annual percentage change. APC=average percentage change. DALY=disability-adjusted life-year.

From 1990 to 2021, the global incidence number of thyroid cancer in AYAs increased by 1.5-fold (95% UI 1.2–2.0). The AAPC for this period was calculated to be 1.73 (95% CI 1.60–1.87) (Table 1), reflecting a significant upward trend. The steepest rise in ASIR was noted between 2003 and 2010 (APC 3.18), followed by a deceleration from 2010 to 2021 (APC 0.75) (Fig 1A). The prevalence number also increased by a factor of 1.5 (95% UI 1.2–2.0), and the ASPR increased with an AAPC of 1.77(95% CI 1.64 to 1.91). The ASPR increased significantly from 2003 to 2010, with an APC of 3.25 (Fig 1B). During this period, the DALYs number rose by 0.6 (95% UI 0.3–1.0). Although there was a downward trend in ASDR from 2000 to 2004 (APC =  −0.21), the p-value was not statistically significant. ASDR continued to show an upward trend from 1990 to 2021, with an AAPC of 0.38 (95% CI 0.25 to 0.51) (Fig 1C).

### Regional level

#### Incidence.

In 2021, adolescents and young adults in Middle SDI region (15.8 thousand; 95% UI [12.6–19.0]) experienced the highest number of thyroid cancer cases among the five SDI regions (Table 1, and S1 Fig in S1 File). High SDI region, meanwhile, had the highest ASIR, with a value of 2.1 (95%CI: 2.0–2.4) per 100,000. Furthermore, Middle SDI region demonstrated the most notable increase in ASIR from 1990 to 2021 (AAPC: 2.68, 95%CI: 2.53 to 2.83) (Table 1, and S2 Fig in S1 File).

**Fig 2:**
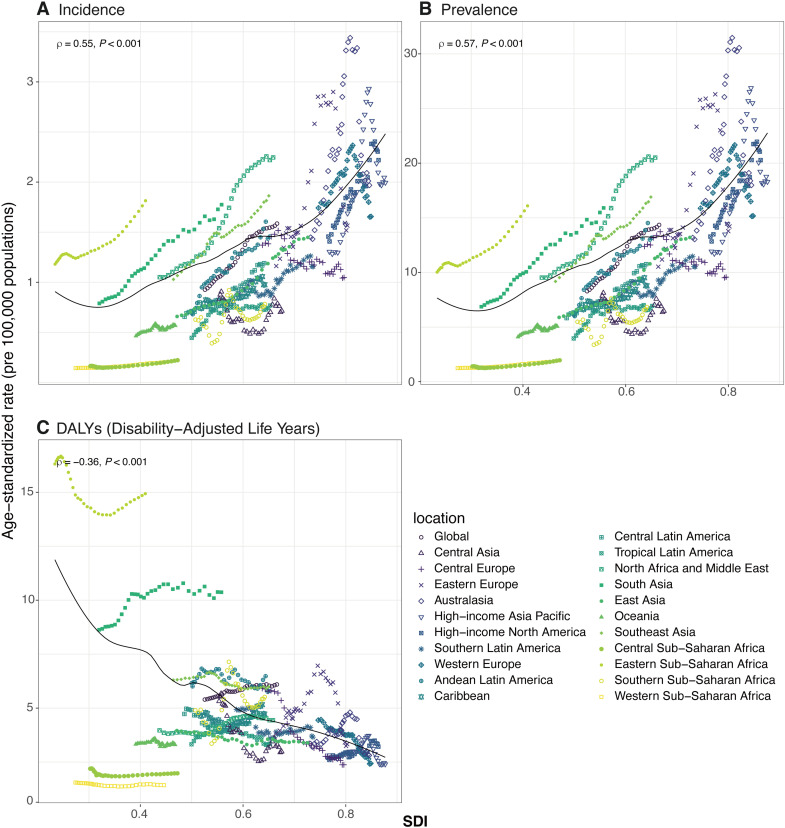
Age-standardized rates for thyroid cancer in adolescents and young adults by SDI across 21 GBD regions with global, from 1990 to 2021. (A), Age-standardized incidence rates; (B), Age-standardized prevalence rates; (C), Age-standardized DALYs rates. SDI = Sociodemographic index. DALYs = disability-adjusted life-years.

Among 21 GBD regions, South Asia reported the highest number of thyroid cancer cases among adolescents and young adults in 2021, at 13.9 thousand (95% UI: 10.5–19.4), whereas Oceania had the fewest (Table 1, and S1 Fig in S1 File). The highest ASIR were recorded in High-income North America and North Africa and the Middle East, both at 2.2 per 100,000 (95% CI [2.1–2.4] and [1.7–2.8], respectively). Conversely, the lowest ASIRs were in Western Sub-Saharan Africa and Central Sub-Saharan Africa, each at 0.2 per 100,000 (95% CI [0.1–0.3] and [0.1–0.4], respectively). Compared to the global average ASIR of 1.6 for thyroid cancer in AYAs, eight regions exceeded this rate, while twelve were below it (Table 1, and S1 Fig in S1 File). From 1990 to 2021, Andean Latin America experienced the most significant rise in ASIR (AAPC: 2.85, 95% CI [2.49 to 3.20]), whereas Oceania showed the smallest (AAPC: 0.60, 95% CI [0.27 to 0.93]). Stability in ASIR was observed in six regions: Western Europe (AAPC: −0.16, 95% CI [−0.32 to 0.01]), Australasia (AAPC:1.24, 95 CI (−0.16 to 2.65]), Central Europe (AAPC: −0.89, 95%CI [−1.78 to 0.01]), Eastern Europe (AAPC: 0.86, 95%CI [−0.48 to 2.21]), Central Asia (AAPC: −0.57, 95%CI [−2.04 to 0.96]), and Southern Sub-Saharan Africa (AAPC: 0.75, 95%CI [−0.03 to 1.53]) (Table 1). Fig 2A illustrates the ASIR trend across 21 GBD regions from 1990 to 2021. Spearman’s rank correlation analysis, with R =  0.55 and p <  0.01, indicated a consistent upward trend in incidence rates alongside increasing SDI.

#### Prevalence.

Within the five SDI regions, the prevalence of thyroid cancer in AYAs followed a distribution similar to incidence, with Middle SDI region having the largest cases of prevalence (143.8 thousand, 95%UI [114.1–173.0]) in 2021, and the most significant ASPR increase (AAPC: 2.75, 95%CI: 2.59 to 2.90) from 1990 to 2021 (Table 2, and S3 Fig in S1 File). Concurrently, High SDI region exhibited the highest ASPR (19.7, 95%CI [17.9–22.3] per 100,000) in 2021 (Table 2, and S3 Fig in S1 File).

For 2021, South Asia reported the highest prevalence number of thyroid cancer in AYAs at 124.1 thousand (95%UI: 94.2–174.6) across GBD regions (Table 2, and S3 Fig in S1 File). North Africa and Middle East had the highest ASPR at 20.5 per 100,000 (95%UI: 15.6–25.9), while Western Sub-Saharan Africa showed the lowest at 1.8 per 100,000 (95%CI: 1.2–2.8). Compared to the global mean of 14.3, 8 regions had higher ASPRs, and 13 had lower rates (Table 2, and S3 Fig in S1 File). Andean Latin America (AAPC: 2.95, 95%CI [2.60 to 3.31]) demonstrated the highest ASPR increase (AAPC: 2.95, 95%CI [2.60–3.31]), and Oceania the lowest (AAPC: 0.63, 95%CI [0.30 to 0.97]). As with ASIR, ASPR remained relatively stable in six regions: Western Europe (AAPC: −0.14, 95% CI [−0.30 to 0.03]), Australasia (AAPC:1.25, 95 CI (−0.14 to 2.67]), Central Europe (AAPC: −0.85, 95%CI [−1.74 to 0.05]), Eastern Europe (AAPC: 0.86, 95%CI [−0.46 to 2.20]), Central Asia (AAPC: −0.54, 95%CI [−2.00 to 0.95]), and Southern Sub-Saharan Africa (AAPC: 0.75, 95%CI [−0.04 to 1.55]) (Table 2). As depicted in Fig 2B, Spearman’s rank correlation analysis (R =  0.57, p <  0.001) indicated a significant upward trend in ASPR among 21 GBD regions associated with increasing SDI between 1990 and 2021.

#### DALYs.

Contrasting with incidence and prevalence, Low-middle SDI region (63.0 thousand, 95%UI [48.3–87.0]) accounted for the largest number of DALYs across the five SDI regions, and Low SDI region shown the highest ASDR (9.4, 95% [6.9–13.9] per 100,000) (Table 3, and S4 Fig in S1 File). In addition, High SDI and High-middle SDI regions shown a downward trend in ASDR, with AAPCs of (−0.44, 95%CI [−0.60 to −0.28]) and (−0.84, 95%CI [−1.21 to −0.47]), respectively (Table 3, and S2 Fig in S1 File).

By 2021, the burden of disease, as measured by DALYs, was highest in South Asia with 81.4 thousand (95%UI [62.3–111.6]), contrasting with Oceania’s lowest of 0.2 thousand (95%UI [0.1–0.3]) (Table 3, and S4 Fig in S1 File). In terms of ASDR, Eastern Sub-Saharan Africa exhibited the highest at 14.9 per 100,000 (95%CI [10.2–23.8]), and Western Sub-Saharan Africa the lowest at 1.4 per 100,000 (95%CI [1.0–2.2]). Globally, only two regions—Eastern Sub-Saharan Africa and South Asia—had ASDRs above the global mean of 6.1, with the remaining 19 regions below this average. Over the period from 1990 to 2021, South Asia showed the most significant rise in ASDR, with an AAPC of 0.55 (95%CI [0.24 to 0.85]), while Central Europe experienced the steepest drop (AAPC: −3.00, 95%CI [−3.88 to −2.11]). During this period, ASDR remained relatively stable in nine regions: High-income North America(AAPC: 0.59, 95%CI [−0.23 to 1.40]), Australasia (AAPC: −0.35, 95%CI [−1.26 to 0.57]), Andean Latin America(AAPC: −0.27, 95%CI [−1.55 to 0.62]), Tropical Latin America(AAPC: −0.09, 95%CI [−0.41 to 0.23]), Central Latin America(AAPC: −0.10, 95%CI [−0.55 to 0.34]), Caribbean (AAPC: −0.08, 95%CI [−0.65 to 0.48]), Eastern Europe (AAPC: −0.53, 95%CI [−1.81 to 0.77]), Oceania (AAPC: 0.02, 95%CI [−0.14 to 0.19]), Southern Sub-Saharan Africa (AAPC: 0.41, 95%CI [−0.18 to 1.01]). In Fig 2C, the ASDR smoothing curves for 21 GBD regions demonstrate a significant decline in ASDR with increasing SDI (R =  −0.36, p <  0.001) from 1990 to 2021.

### National level

#### Incidence.

**Fig 3:**
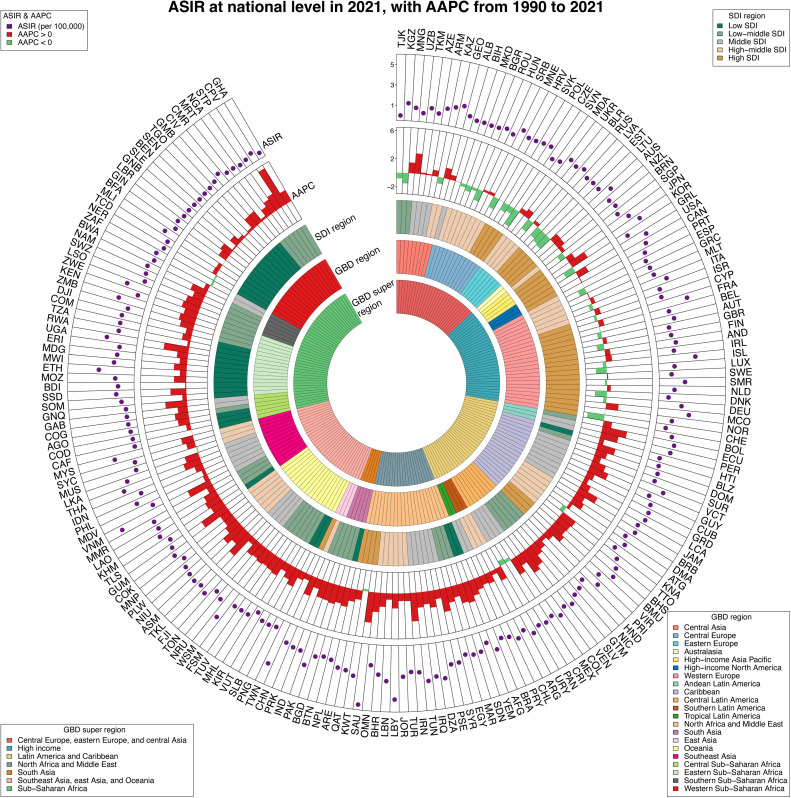
ASIR of thyroid cancer among adolescents and young adults at national level in 2021, and their AAPCs from 1990 to 2021. From outermost to innermost, the layers are ASIR, AAPC, 5 SDI regions, 21 GBD regions, and 7 GBD super regions. The 204 countries and territories were designated by their ISO-3 codes, and a list of their full names can be found in S5 Table of S1 File. ASIR = Age-standardized incidence rates. AAPC=average annual percentage change. SDI = Sociodemographic index.

For the year 2021, India (9.7 thousand; 95% UI [7.5–13.2]), China (7.5 thousand; 95%UI [5.9–10.0]), and Pakistan (2.7 thousand; 95%UI [2.5–2.9]) demonstrated the most incidence cases of thyroid cancer among AYAs (S5 Table, and S5 Fig in S1 File). Meanwhile, Saudi Arabia (5.5; 95% CI [2.9–9.7] per 100,000), Libya (4.8; 95%CI [2.3–9.0] per 100,000), and Viet Nam (4.3; 95%CI [1.9–8.0] per 100,000) experienced the highest ASIR of thyroid cancer among AYAs (S5 Table in S1 File, and Fig 3). Out of all countries/territories, 48 had an ASIR above the global average, 149 below, and the remaining 7 were at the global average (S5 Table in S1 File, and Fig 3). Cabo Verde, Saudi Arabia, and Guam recorded the steepest increases in ASIR between 1990 and 2021, with AAPCs of 5.99 (95% CI: 5.34 to 6.64), 4.37 (95% CI: 4.16 to 4.57), and 4.17(95% CI: 2.40 to 5.96), respectively (S5 Table in S1 File, and Fig 3). S6 Fig in S1 File presents the ASIR trend among 204 countries in 2021. The Spearman’s rank correlation analysis, demonstrating R =  0.35 and p <  0.01, pointed to a consistent increase in ASIR as SDI increased.

#### Prevalence.

**Fig 4:**
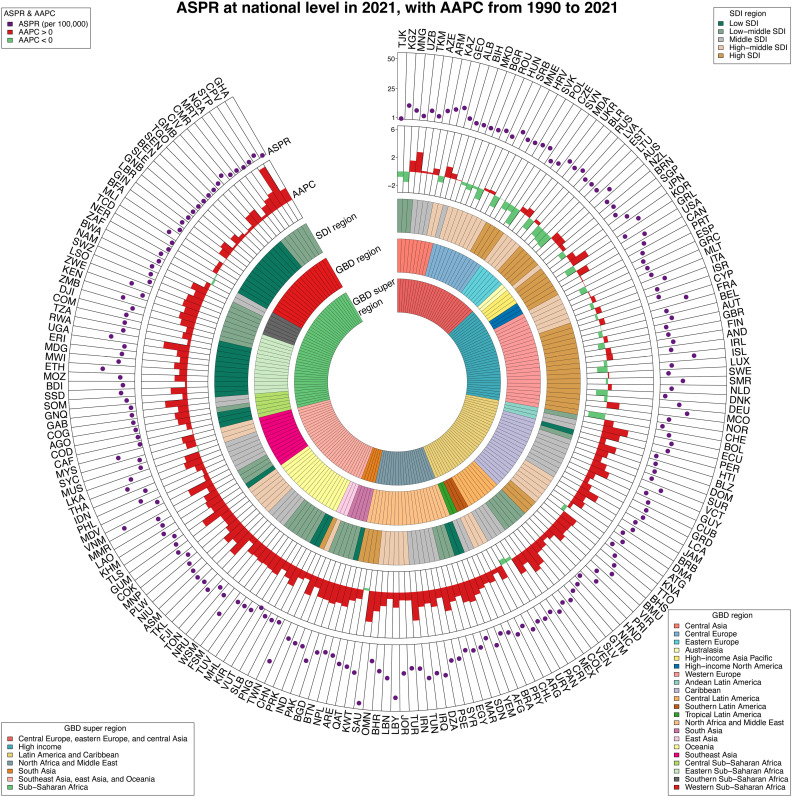
ASPR of thyroid cancer among adolescents and young adults at global level in 2021, and their AAPCs from 1990 to 2021. From outermost to innermost, the layers are ASPR, AAPC, 5 SDI regions, 21 GBD regions, and 7 GBD super regions. The 204 countries and territories were designated by their ISO-3 codes, and a list of their full names can be found in S5 Table of S1 File. ASPR = Age-standardized prevalence rates. AAPC=average annual percentage change. SDI = Sociodemographic index.

In 2021, India (87.6 thousand; 95%UI [67.0–119.0]), China (68.6 thousand; 95%UI [53.6–90.9]), and United States of America (24.3 thousand; 95%UI [22.8–26.1]) exhibited the highest number of AYAs thyroid cancer associated prevalence (S6 Table, and S7 Fig in S1 File). Concurrently, Saudi Arabia (50.0; 95%CI [26.7–88.1] per 100,000), Libya (43.7; 95%CI [20.7–82.7] per 100,000), and Viet Nam (38.8; 95%CI [16.9–73.2] per 100,000) reported the highest ASPR (S6 Table in S1 File, and Fig 4). The ASPR was above average in 53 countries/regions, below in 149, and equal to the global average in the remaining 2 (S6 Table in S1 File, and Fig 4). At national level, the most significant increase in ASPR from 1990 to 2021 were observed in the Cabo Verde (AAPC: 6.03, 95%CI [5.39 to 6.67]), Saudi Arabia (AAPC:4.39, 95%CI [4.18 to 4.60]), and Guam (AAPC:4.16, 95%CI [2.40 to 5.96]) (S6 Table in S1 File, and Fig 4). S6 Fig in S1 File illustrates the ASPR trend across 204 countries/territories in 2021, with Spearman’s rank correlation analysis (R =  0.36, p <  0.01) indicating a significant upward trend in ASPR as SDI values increased.

#### DALYs.

**Fig 5:**
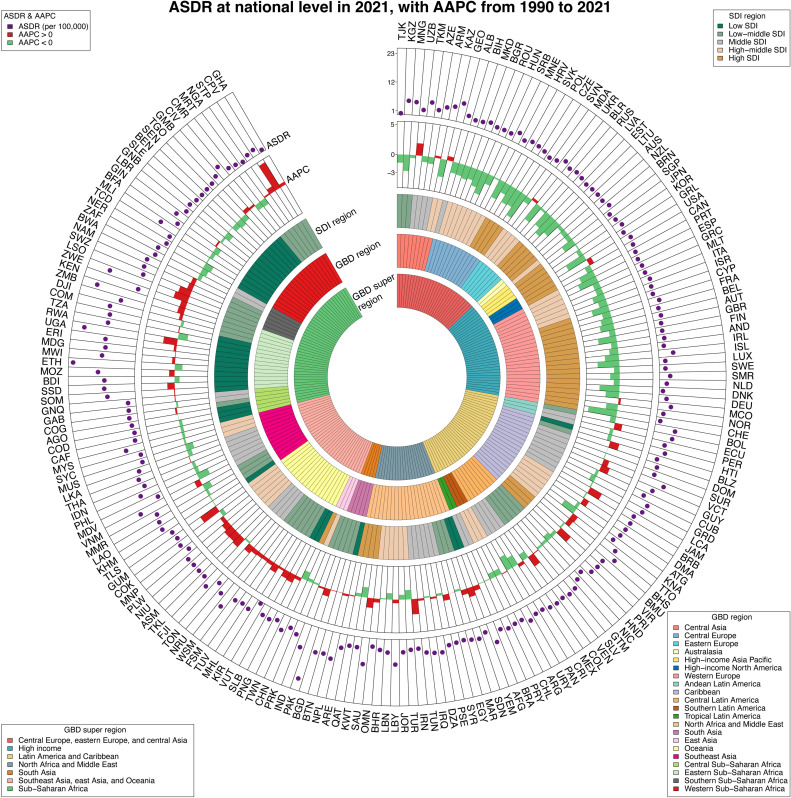
ASDR of thyroid cancer among adolescents and young adults at global level in 2021, and their AAPCs from 1990 to 2021. From outermost to innermost, the layers are ASDR, AAPC, 5 SDI regions, 21 GBD regions, and 7 GBD super regions. The 204 countries and territories were designated by their ISO-3 codes, and a list of their full names can be found in S6 Table of S1 File. ASDR = Age-standardized DALYs rates. DALY = disability-adjusted life-year. AAPC = average annual percentage change. SDI = Sociodemographic index.

By 2021, India (54.0; 95%UI [41.8–72.0]), Pakistan (19.9 thousand; 95%UI [12.3–31.5]), and China (17.2 thousand; 95%UI [13.4–22.4]) demonstrated the highest number of AYAs thyroid cancer associated DALYs (S7 Table, and S8 Fig in S1 File). Additionally, Ethiopia (23.5; 95%CI [14.3–41.6] per 100,000), Pakistan (20.5; 95%CI [12.7–32.3] per 100,000), and Uganda (20.3; 95%CI [11.5–35.9] per 100,000) exhibited the highest ASDR of thyroid cancer in AYAs (S7 Table in S1 File, and Fig 5). Out of all countries/territories, 37 reported an ASIR above the global mean, while 167 were below (S7 Table in S1 File, and Fig 5). Among the 204 countries, the three countries experiencing the sharpest increase in the ASDR over 1990–2021 were the Cabo Verde (AAPC: 4.91, 95%CI [4.01 to 5.81]), Guam (AAPC: 3.14, 95%CI [1.55 to 4.76]), and Iran (Islamic Republic of) (AAPC: 2.52, 95%CI [2.15 to 2.89]) (S7 Table in S1 File, and Fig 5). The ASDR smoothing curve for 204 countries/territories highlights a significant decline in ASDR with increasing SDI values (R =  −0.26, p <  0.001) in 2021 (S6 Fig in S1 File).

**Fig 6:**
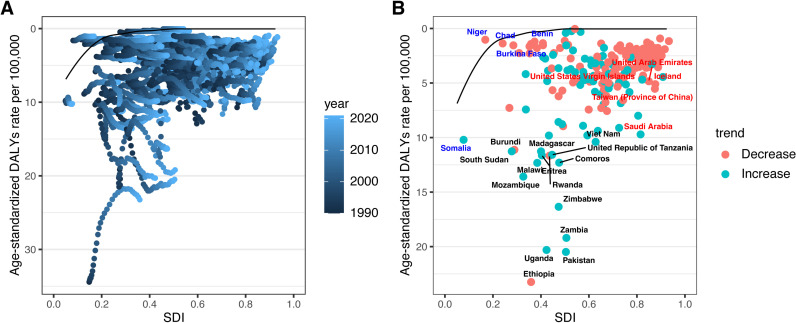
Frontier analysis based on SDI and age-standardized DALYs rate for thyroid cancer among adolescents and young adults. A. Frontier analysis based on SDI and ASDR from 1990 to 2021. B. Frontier analysis based on SDI and ASDR in 2021. The solid black line signifies the frontier, with dots indicating countries and territories. The 15 countries with the greatest effective differences are labeled black. Countries/territories with high SDI (>0.8103) and a high effective difference are labeled red, and those with low SDI (<0.4658) and a small effective difference for their development are labeled blue. Red dots indicate an increase, and blue dots a decrease, in ASDR from 1990 to 2021.

To explore possible reductions in ASDR for TC among AYAs, frontier analysis was performed using SDI values as a factor. The results revealed that the effective difference for a given SDI generally decreased with increasing SDI globally (S8 Table in S1 File, Fig 6). The 15 countries or territories exhibiting the largest effective differences, ranging from 10.24 to 22.87, included South Sudan, Viet Nam, Burundi, Madagascar, United Republic of Tanzania, Eritrea, Rwanda, Malawi, Comoros, Mozambique, Zimbabwe, Zambia, Uganda, Pakistan, and Ethiopia. Notably, five countries or territories with high SDI values (>0.8103), such as Saudi Arabia, Taiwan (Province of China), Iceland, United Arab Emirates, and United States Virgin Islands, exhibited relatively high effective differences (4.65–9.67). Conversely, five countries or territories with low SDI (<0.4658), including Niger, Chad, Burkina Faso, Benin, and Somalia, demonstrated smaller effective differences (0.00–0.85).

### Age and sex patterns

Globally, in 2021, the incidence of new TC cases across five-year age groups from 15 to 39 years showed a progressive increase, with the 35–39 age group peaking at 18.0 thousand cases (95%UI: 15.6–20.6) (S9–S11 Tables in S1 File). This age group also recorded the highest numbers of incidence, prevalence, and DALYs from 1990 to 2021 (S9–S11 Tables, and S9 Fig in S1 File). Specifically, in 2021, the 35–39 age group had an age special incidence rate of 3.2 per 100,000 (95% CI: 2.8–3.7), an age special prevalence rate of 29.0 per 100,000 (95% CI: 25.2–33.3), and an age special DALYs rate of 9.39 per 100,000 (95%CI: 7.8–11.1). From 1990 to 2021, a global increase in the numbers of incidence, prevalence, and DALYs for TC was observed in all five age groups among AYAs. Notably, the 30–34 age group experienced the most significant rise in age special rates for TC incidence (AAPC:3.36, 95%CI [3.19 to 3.52]), prevalence (AAPC:3.39, 95%CI [3.23 to 3.55]), and DALYs (AAPC:1.88, 95%CI [1.78 to 1.99]) (S9–S11 Tables, and S10 Fig in S1 File).

Throughout the period from 1990 to 2021, females had a higher burden of thyroid cancer, as reflected in incidence, prevalence, and DALYs, compared to males (S10 and S11 Figs in S1 File). In 2021, new cases were recorded as 13.3 thousand for males (96%UI: 11.1–15.1) and 34.9 thousand for females (95%UI: 28.2–44.9) (Table 1). Females also exhibited higher ASRs for incidence, prevalence, and DALYs during this period (Fig 1). Consistently, in 2021, females demonstrated higher age-specific rates of incidence, prevalence, and DALYs in each five-year age group compared to males (S9–S11 Tables, and S9 Fig in S1 File). From 1990 to 2021, the ASIR of thyroid cancer among AYAs increased in both males and females, with corresponding AAPC values of 2.15 (95%CI: 2.02–2.30) and 1.56 (95%CI: 1.38–1.74), respectively (Table 1, and Fig 1). An increasing trend was also noted in ASPR (males: AAPC =  2.22; 95%CI [2.08 to 2.35]; females: AAPC =  1.60; 95%CI [1.42 to 1.77]) and in ASDR (males: AAPC = 0.64; 95%CI (0.49 to 0.78); females: AAPC = 0.22; 95%CI [0.01 to 0.44]) during the same period (Tables 2, 3, and Fig 1). While AYA females exhibited higher ASRs for thyroid cancer incidence, prevalence, and DALYs, the AAPC indicated a steeper increase in these metrics for males.

### Decomposition analysis

**Fig 7:**
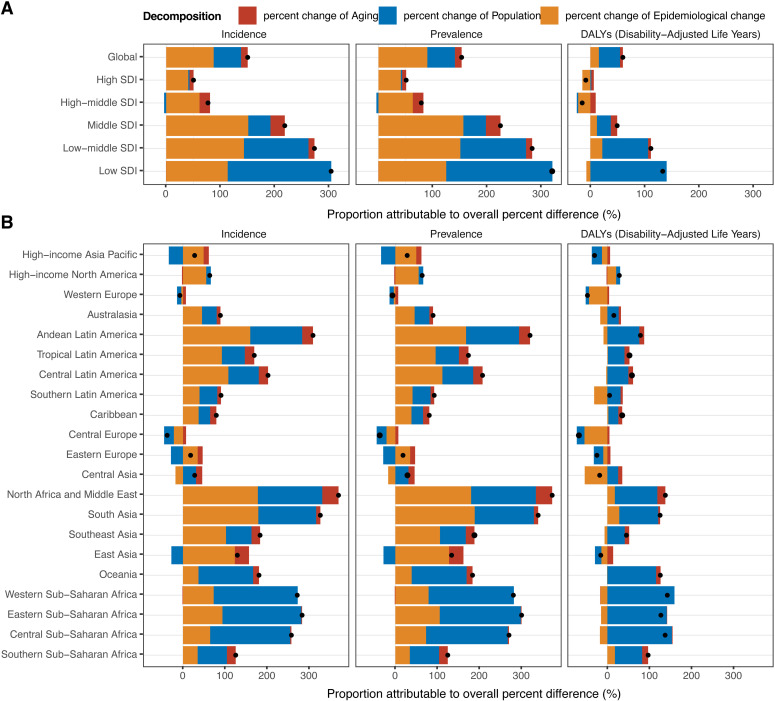
Decomposition analysis of thyroid cancer burden among adolescents and young adults from 1990 to 2021. (A) Global and SDI regions, (B) 21 GBD regions. The black dot represents the overall change value of population growth, aging, and epidemiological change. SDI = Sociodemographic index.

Decomposition analyses from 1990 to 2021 revealed a significant increase in the burden of thyroid cancer among AYAs, with rising incidence, prevalence, and DALYs. By 2021, the number of prevalent cases had increased by 263.9 thousand cases, a 153.2% rise from 1990. New case numbers also saw a significant rise, with an additional 28.9 thousand cases, a 150.2% increase. Additionally, the DALYs due to thyroid cancer increased by 68.8 thousand, a 59.9% rise from 1990 (Fig 7A, and S12–14 Tables in S1 File).

Among the five SDI regions, the influence of population aging on thyroid cancer of incidence, prevalence, and DALYs among AYAs was most pronounced in the Middle SDI region, contributing 26.4%, 26.8%, and 11.7% respectively. Population growth had the greatest impact in Low SDI region, with contributions of 190.9%, 195. 7%, and 140.6% to these metrics. Epidemiological changes in the Middle SDI region increased incidence and prevalence by 151.2% and 156.4%, respectively, contrasting with a 22.3% reduction in DALYs in the High-middle SDI region (Fig 7A, and S12–14 Tables in S1 File).

Although the thyroid cancer burden has generally risen in most GBD regions among AYAs, Western Europe and Central Europe experienced a decrease in these metrics: −7.4%, −6.9%, and −47.2% for Western Europe, and −37.7%, −37.0%, and −67.6% for Central Europe. Furthermore, the contribution of population growth in High-income Asia Pacific, Western Europe, Central Europe, Eastern Europe, and East Asia to incidence (−33.7%, −10.1%, −23.3%, −28.7% and −27.7%), prevalence (−33.8%, −10.1%, −23.4%, −28.7%, and −28.1%), and DALYs (−24.2%, −7.9%, −18.3%, −22.5, and −15.3%) was also negative. In terms of contributing factors to prevalence, population aging was highest in North Africa and the Middle East at 38.8%, population growth was most pronounced in western sub-Saharan South Africa at 202.7%, and epidemiological changes were most impactful in South Asia at 188.7% (Fig 7B, and S12–14 Tables in S1 File).

### Prediction analysis of thyroid cancer in AYAs till 2040

**Fig 8:**
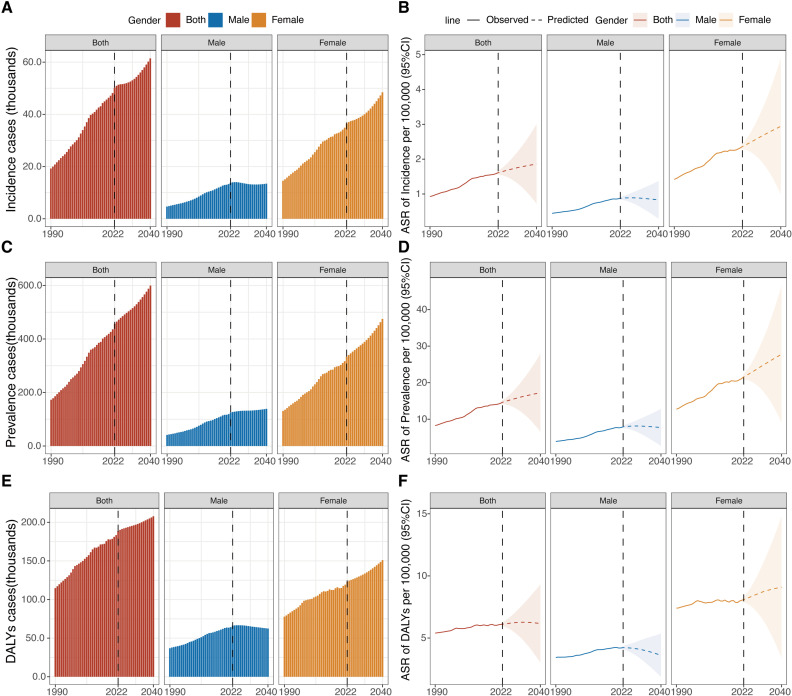
BAPC prediction of thyroid cancer burden by sex among adolescents and young adults from 1990 to 2040. (A), Number of incidence cases; (B), Age-standardized incidence rates (ASIR); (C), Number of prevalence cases; (D), Age-standardized prevalence rates (ASPR); (E), Number of DALYs; (F), Age-standardized DALYs rates (ASDR). BAPC = Bayesian Age-Period-Cohort model. DALYs = disability-adjusted life-years.

Based on GBD 2021 study, our research projected a global increase in thyroid cancer burden for AYAs from 2022 to 2040. Fig 8, utilizing the BAPC model, anticipated a general upward trend in burden for both genders, with males exhibiting a distinct pattern of initial increase and subsequent decline. In addition, the ASIR, ASPR, and ASDR for thyroid cancer in the AYAs were projected to remain higher in women than in men over the next 19 years (Fig 8, and S15 Table in S1 File). By 2040, global predictions had foreseen 60.2 thousand incident cases, 558.4 thousand prevalent cases and 199.7 thousand DALYs due to thyroid cancer among AYAs (S14 Table in S1 File).

## Discussion

Employing the most recent GBD 2021 database, our study, which we believe to be the first, offered an in-depth analysis of the global, regional, and national burden of thyroid cancer among AYAs over 32 years (1990–2021). The key findings include: First, the numbers and ASRs of incidence, prevalence, and DALYs for TC among AYAs demonstrated a substantial rise worldwide between 1990 and 2021. Second, disease burden exhibited significant disparities across SDI and GBD regions. As SDI values rose, there was a corresponding increase in ASIR and ASPR. In contrast, ASDR decreased with higher SDI values. Third, women consistently bore a greater disease burden than men between 1990 and 2021. Meantime, the age group 30-34 experienced the sharpest rise in disease burden, while the 35–39 age group maintained the highest disease burden. Lastly, it was anticipated that the global disease burden of thyroid cancer among AYAs would continue to increase by 2040, particularly for females.

From 1990 to 2021, the global burden of TC among AYAs, quantified by the numbers and ASRs of incidence, prevalence, and DALYs, increased significantly. Despite limited understanding of the causes, the increasing trends of TC in AYAs may be attributed to several risk factors. First, lifestyle changes, such as cumulative exposure to metabolic syndrome [22–24], use of mobile phones [25,26], reduced sleep duration [27] and dietary habits [28], may lead to an increased incidence and prevalence of TC in AYAs. Second, the widespread adoption of advanced diagnostic methods, including ultrasonography and fine-needle aspiration, facilitates early detection of TC [29–32]. Third, specific environmental factors, such as exposure to PFAS [33,34] and exposure to ionizing radiation [35–37], may contribute to an increased incidence of thyroid cancer.

The SDI is a composite indicator that measures the development status of a country or region, closely correlated with health outcomes, reflecting the interplay between social, economic, and demographic factors. It serves as a crucial tool for predicting and understanding health trends and disparities across different global contexts [38,39]. An increase in the SDI is linked to a consistent upward trend in the ASIR and ASPR across the majority of the GBD regions, with Middle SDI regions such as Andean Latin America and East Asia experiencing the most pronounced increases. This could be primarily attributed to their recent rapid development and higher rates of TC detection. Conversely, the ASDR for TC among AYAs was observed to decline with an increase in High SDI regions, particularly in Western and Central Europe, where early diagnosis and improved treatment played a critical role. Interestingly, despite possessing advanced healthcare infrastructure and resources, five countries or territories—Saudi Arabia, Taiwan (Province of China), Iceland, the United Arab Emirates, and the United States Virgin Islands—still perform well below the frontier line. Optimizing the allocation of healthcare resources, ensuring equitable access to healthcare services, and developing individual treatment plans may reduce the burden of ASDR in these high SDI countries [40]. To further dissect the factors affecting incidence, prevalence, and DALYs of TC in AYAs over the past 32 years, we dissected the incidence, prevalence, and DALYs across three dimensions: population aging, population growth, and epidemiological changes. In Middle SDI regions, epidemiological changes were the primary driver of the 151.2% and 156.4% increase in incidence and prevalence, respectively. Similarly, High and Middle-High SDI regions also predominantly experienced epidemiological changes affecting the reduction in the burden of TC DALYs, with respective contributions of 14.7% and 22.4%. By contrast, Low SDI region was predominantly affected by population growth, which resulted in substantial increases in incidence, prevalence, and DALYs of 190.9%, 195. 7%, and 140.6%, respectively.

According to Global Cancer Observatory (GLOBOCAN) 2022 (https://gco.iarc.fr/) [41], the global ASIR for thyroid cancer in the AYA group was reported to 7.31 per 100,000, with the highest rate observed in North America (10.91), followed by Asia (9.14), Oceania (7.18), and Europe (6.53), Latin America and the Caribbean (6.18), and the lowest rates in Africa (0.92). These results exhibited some discrepancies with our study’s outcomes, potentially because of the following factors: First, the delineation of regions varies; for instance, GLOBOCAN encompasses Oceania, which includes the high-incidence Australia, whereas GBD categorizes Australia under Australasia [42]. Second, there is a difference in data sources: GLOBOCAN mainly relies on cancer registries or regional reports, which cover high-income regions more broadly but are less present in low- and middle-income countries (LMICs), where there is a lack of national registries [43,44]. GBD, on the other hand, accesses a wide range of data sources, including cancer registries, hospital records, population surveys, and death reports [8,45]. Third, the age-standardization approaches differ: GLOBOCAN utilizes the WHO World Standard Population for age-standardizing data, highlighting the disease characteristics of aging populations. GBD, conversely, reflects the actual population structures of various regions and considers multidimensional factors like age, gender, and socioeconomic status. Furthermore, previous studies have also noted inconsistencies in ASIR of oral and gastrointestinal cancers between two databases, with the most significant differences in LMICs lacking well-established cancer registries and healthcare administrative systems [43,44]. Given the observed inconsistencies in ASIR of thyroid cancer between GLOBOCAN and GBD, it could be valuable to consider the following strategies: 1) Establish and enhance cancer registration systems, prioritizing the development of population-based cancer registries in LMICs; 2) Advance modeling and estimation approaches to enhance the accuracy of regional and global cancer burden estimations; 3) Foster international collaboration to achieve a balanced improvement in the quality of worldwide cancer data.

Between 1990 and 2021, gender differences in the global burden of thyroid cancer among AYAs were noted, with women consistently exhibiting a higher disease burden than men. This is consistent with previous studies reporting a higher incidence in women than men in all age groups [1,32]. This disparity may be attributed to four main factors. First, women’s greater propensity to seek and utilize healthcare services, which provides them with more screening opportunities [46]. Second, the influence of estrogen [47], which promotes tumor growth through genomic and non-genomic pathways mediated by membrane-bound receptors and also modulates tumor progression by affecting the tumor microenvironment [48]. Third, women’s immune systems differ significantly from men’s, leading to a higher risk of autoimmune diseases such as Hashimoto’s thyroiditis, which is associated with an increased risk of thyroid cancer [49]. Autoimmune reactions may promote chronic inflammation of thyroid cells, potentially raising the risk of thyroid cancer [50]. Finally, androgen stimulation of the androgen receptor suppresses the growth of thyroid cancer cells, potentially accounting for the lower incidence in men [51,52]. These findings emphasize the need for gender-specific strategies in the prevention, diagnosis, and treatment of thyroid cancer.

Using BAPC models and GBD projections for global population trends, we predicted a continuous increase in the numbers and ASRs of incidence, prevalence, DALYs of thyroid cancer in AYAs from 2022 to 2040, particularly in females. By 2040, the incidence ratio of thyroid cancer between males and females was expected to be around 1:3, with the prevalence ratio nearing 1:4. Contributing factors might include global population growth, enhanced cancer detection, and growing exposure of children to electronic devices, which could increase future cancer risks due to ionizing radiation [36]. Interestingly, in AYAs males, ASIR were projected to rise, and then decline, with a similar trend observed in ASPR and ASDR. Further investigation is needed to clarify the role of sex hormones [48,52] or other influences in these trends.

### Limitations

This comprehensive assessment filled gaps in epidemiological data on thyroid cancer among AYAs, providing crucial insights for developing effective public health strategies to address this growing challenge. However, there were several limitations inherent in this study which should be acknowledged. Firstly, the data from the GBD study did not include pathological subtypes of thyroid cancer, which limited the depth of the analysis. Secondly, the varying quality and completeness of data in LMICs often lead to the use of estimates, which can introduce inaccuracies in the assessment of the true disease burden. Thirdly, the uneven global distribution of healthcare resources resulted in disparities that could cause underdiagnosis or misdiagnosis in regions with limited medical infrastructure, potentially masking the true extent of disease prevalence. Finally, the continuity and comparability of data was complicated by the evolution of diagnostic criteria and techniques during the 32-year study, which might impact the long-term analysis of disease trends, as newer technologies might increase the detection rate of thyroid cancer. Therefore, ongoing validation through more real-world studies is necessary in the future.

## Conclusion

Thyroid cancer in adolescents and young adults represents a major public health concern worldwide. Between 1990 and 2021, there was a consistent increase in the age-standardized rates of incidence, prevalence, and DALYs for thyroid cancer in this demographic, with females consistently showing higher disease burden than males. Significant disparities existed across regions and countries, with increased SDI values correlating with higher ASIR and ASPR, yet contrastingly, ASDR declined as SDI values rose. Furthermore, the 35-39 age group exhibited the highest disease burden across the five age groups. BAPC model projections suggested that the numbers and ASRs would continue to increase globally from 2022 to 2040. Effective resource allocation and strategic preventive and therapeutic measures are essential for managing thyroid cancer among AYAs and ensuring a balanced health landscape worldwide.

## Supporting information

S1 File S1 Table. The Guidelines for Accurate and Transparent Health Assessment Reporting (GATHER). S2 Table. The Socio-demographic Index (SDI) reference values from the Global Burden of Disease (GBD) data released by the Institute for Health Metrics and Evaluation (IHME) in 2021. S3 Table. The Socio-demographic Index (SDI) values of 204 countries/territories from the Global Burden of Disease (GBD) data released by the Institute for Health Metrics and Evaluation (IHME) in 2021. S4 Table. The correspondence of Global Burden of Disease (GBD) regions with 204 countries/territories from the GBD data released by the Institute for Health Metrics and Evaluation (IHME) in 2021. S5 Table. Incidence of thyroid cancer in adolescents and young adults (AYAs), and their average annual percentage changes in 204 countries/territories from 1990 to 2021. S6 Table. Prevalence of thyroid cancer in adolescents and young adults (AYAs), and their average annual percentage changes in 204 countries/territories from 1990 to 2021. S7 Table. DALYs of thyroid cancer in adolescents and young adults (AYAs), and their average annual percentage changes in 204 countries/territories from 1990 to 2021. DALY = disability-adjusted life-year. S8 Table. The results of frontier analysis based on SDI and age-standardized DALYs rate for thyroid cancer in adolescents and young adults from 1990 to 2021. S9 Table. Incidence of thyroid cancer in adolescents and young adults (AYAs), and their average annual percentage changes from 1990 to 2021 by age at the global level. S10 Table. Prevalence of thyroid cancer in adolescents and young adults (AYAs), and their average annual percentage changes from 1990 to 2021 by age at the global level. S11 Table. DALYs of thyroid cancer in adolescents and young adults (AYAs), and their average annual percentage changes from 1990 to 2021 by age at the global level. DALY = disability-adjusted life-year. S12 Table. Decomposition of the percentage changes in thyroid cancer among adolescents and young adults (AYAs) worldwide from 1990 to 2021, alterations in incidence as influenced by aging, population growth, and epidemiological change. S13 Table. Decomposition of the percentage changes in thyroid cancer among adolescents and young adults (AYAs) worldwide from 1990 to 2021, alterations in prevalence as influenced by aging, population growth, and epidemiological change. S14 Table. Decomposition of the percentage changes in thyroid cancer among adolescents and young adults (AYAs) worldwide from 1990 to 2021, alterations in DALYs as influenced by aging, population growth, and epidemiological change. DALYs = disability-adjusted life-years. S15 Table. Prediction results of Bayesian age–period–cohort analysis (BAPC) in thyroid cancer among adolescents and young adults (AYAs) worldwide in 2040. S1 Fig. Cases and age-standardized rates of incidence at the regional level in 1990 and 2021. (A) Incidence cases; (B) Age-standardized incidence rates. S2 Fig. The APCs of ASR for thyroid cancer among adolescents and young adults (AYAs) at the global and regional level based on the Joinpoint regression analysis model. (A) Incidence cases; (B) Prevalence cases; (C) DALYs cases. DALYs = disability-adjusted life-years. S3 Fig. Cases and age-standardized rates of prevalence at the regional level in 1990 and 2021. (A) Prevalence cases; (B) Age-standardized prevalence rates. S4 Fig. Cases and age-standardized rates of DALYs at the regional level in 1990 and 2021. (A) Prevalence cases; (B) Age-standardized prevalence rates. DALYs = disability-adjusted life-years. S5 Fig. Numbers of incidence (thousand) for thyroid cancer among adolescents and young adults at global level in 2021. S6 Fig. Age-standardized rates for thyroid cancer in adolescents and young adults by SDI across 204 countries/territories in 2021. (A), Age-standardized incidence rates; (B), Age-standardized prevalence rates; (C), Age-standardized DALYs rates. SDI = Sociodemographic index. DALYs = disability-adjusted life-years. S7 Fig. Number of prevalence (thousand) for thyroid cancer among adolescents and young adults at global level in 2021. S8 Fig. Number of DALYs (thousand) for thyroid cancer among adolescents and young adults at global level in 2021. S9 Fig. Trends of incidence, prevalence and DALYs for thyroid cancer among adolescents and young adults from 1990 to 2021. (A) Trends in incident cases and age special incidence rate. (B) Trends in death cases and age special death rate. (C) Trends in DALYs cases and age special DALYs rate. DALYs = disability-adjusted life-years. S10 Fig. The results of Joinpoint regression analysis in five age groups at the global level from 1990 to 2021. (A) Incidence cases; (B) Prevalence cases; (C) DALYs cases. DALYs = disability-adjusted life-years. S11 Fig. The trends of numbers in incidence (A), prevalence (B), and DALYs (C) for thyroid cancer among adolescents and young adults by sex, from 1990 to 2021. DALYs = disability-adjusted life-years.
(PDF)

## Abbreviations

TC: Thyroid cancer
AYAs: Adolescents and young adults
GBD: Global Burden of Disease
DALYs: Disability-adjusted life-years
BAPC: Bayesian age-period-cohort
SDI: Sociodemographic Index
ASR: Age-standardized rate
ASIR: Age-standardized incidence
ASPR: Age-standardized prevalence
ASDR: Age-standardized DALY rate
APC: Annual percentage change
AAPC: Average annual percentage change
UI: Uncertainty interval
CI: Confidence interval
LMICs: Low- and middle-income countries.
