# Supplementary material for: Global burden of thyroid cancer in adolescents and young adults (aged 15–39 years) from 1990 to 2021: A systematic analysis of the Global Burden of Disease Study 2021
Source: PLoS One. 2025 Feb 14;20(2):e0318605. doi: 10.1371/journal.pone.0318605 (PMC11828416; doi:10.1371/journal.pone.0318605)

Global burden of thyroid cancer in adolescents and young adults (aged 15-39 years) from 1990 to 2021: a systematic analysis of the Global Burden of Disease Study 2021

Zijian Qiu[1], Shengjian Yu[2], Lin Zheng[3], Ying Lou[4], Xiuxia Chen[5], Feng Xuan[2, *]

S1 File:

Table S1: The Guidelines for Accurate and Transparent Health Assessment Reporting (GATHER).

Table S2: The Socio-demographic Index (SDI) reference values from the Global Burden of Disease (GBD) data released by the Institute for Health Metrics and Evaluation (IHME) in 2021.

Table S3. The Socio-demographic Index (SDI) values of 204 countries/territories from the Global Burden of Disease (GBD) data released by the Institute for Health Metrics and Evaluation (IHME) in 2021.

Table S4: The correspondence of Global Burden of Disease (GBD) regions with 204 countries/territories from the GBD data released by the Institute for Health Metrics and Evaluation (IHME) in 2021.

Table S5: Incidence of thyroid cancer in adolescents and young adults (AYAs), and their average annual percentage changes in 204 countries/territories from 1990 to 2021.

Table S6: Prevalence of thyroid cancer in adolescents and young adults (AYAs), and their average annual percentage changes in 204 countries/territories from 1990 to 2021.

Table S7: DALYs of thyroid cancer in adolescents and young adults (AYAs), and their average annual percentage changes in 204 countries/territories from 1990 to 2021.

Table S8. The results of frontier analysis based on SDI and age-standardized DALYs rate for thyroid cancer in adolescents and young adults from 1990 to 2021.

Table S9: Incidence, of thyroid cancer in adolescents and young adults (AYAs), and their average annual percentage changes from 1990 to 2021 by age at the global level.

Table S10: Prevalence of thyroid cancer in adolescents and young adults (AYAs), and their average annual percentage changes from 1990 to 2021 by age at the global level.

Table S11: DALYs of thyroid cancer in adolescents and young adults (AYAs), and their average annual percentage changes from 1990 to 2021 by age at the global level.

Table S12: Decomposition of the percentage changes in thyroid cancer among adolescents and young adults (AYAs) worldwide from 1990 to 2021, alterations in incidence as influenced by aging, population growth, and epidemiological change.

Table S13 Decomposition of the percentage changes in thyroid cancer among adolescents and young adults (AYAs) worldwide from 1990 to 2021, alterations in prevalence as influenced by aging, population growth, and epidemiological change.

Table S14: Decomposition of the percentage changes in thyroid cancer among adolescents and young adults (AYAs) worldwide from 1990 to 2021, alterations in DALYs as influenced by aging, population growth, and epidemiological change.

Table S15: Prediction results of Bayesian age–period–cohort analysis (BAPC) in thyroid cancer among adolescents and young adults (AYAs) worldwide in 2040.

Figure S1: Cases and age-standardized rates of incidence at the regional level in 1990 and 2021. (A) Incidence cases; (B) Age-standardized incidence rates.

Figure S2. The APCs of ASR for thyroid cancer among adolescents and young adults (AYAs) at the global and regional level based on the Joinpoint regression analysis model. (A) Incidence cases; (B) Prevalence cases; (C) DALYs cases. DALYs=disability-adjusted life-years.

Figure S3. Cases and age-standardized rates of prevalence at the regional level in 1990 and 2021. (A) Prevalence cases; (B) Age-standardized prevalence rates.

Figure S4. Cases and age-standardized rates of DALYs at the regional level in 1990 and 2021. (A) DALYs cases; (B) Age-standardized DALYs rates. DALYs=disability-adjusted life-years.

Figure S5. Numbers of incidence (thousand) for thyroid cancer among adolescents and young adults at global level in 2021.

Figure S6. Age-standardized rates for thyroid cancer in adolescents and young adults by SDI across 204 countries/territories in 2021. (A), Age-standardized incidence rates; (B), Age-standardized prevalence rates; (C), Age-standardized DALYs rates. SDI= Sociodemographic index. DALYs=disability-adjusted life-years.

Figure S7. Number of prevalence (thousand) for thyroid cancer among adolescents and young adults at global level in 2021.

Figure S8. Number of DALYs (thousand) for thyroid cancer among adolescents and young adults at global level in 2021.

Figure S9. Trends of incidence, prevalence and DALYs for thyroid cancer among adolescents and young adults from 1990 to 2021. (A) Trends in incident cases and age special incidence rate. (B) Trends in death cases and age special death rate. (C) Trends in DALYs cases and age special DALYs rate. DALYs=disability-adjusted life-years.

Figure S10. The results of Joinpoint regression analysis in five age groups at the global level from 1990 to 2021. (A) Incidence cases; (B) Prevalence cases; (C) DALYs cases. DALYs=disability-adjusted life-years.

Figure S11. The trends of numbers in incidence (A), prevalence (B), and DALYs (C) for thyroid cancer among adolescents and young adults by sex, from 1990 to 2021. DALYs=disability-adjusted life-years.

Table S1: The Guidelines for Accurate and Transparent Health Assessment Reporting (GATHER).

**Checklist of information that should be included in new reports of global health estimates**		
Item	Checklist item	Reported on page#
**Objectives and funding**		
**1**	Define the indicator(s), populations (including age, sex, and geographic entities), and time period(s)for which estimates were made	pages:3-5
**2**	List the funding sources for the work.	NA
**Data Inputs**		
**For all data inputs from multiple sources that are synthesized as part of the study:**		
**3**	Describe how the data were identified and how the data were accessed.	pages:3-7
**4**	Specify the inclusion and exclusion criteria. Identify all ad-hoc exclusions.	pages:3-7
**5**	Provide information on all included data sources and their main characteristics. For each data source used, report reference information or contact name/institution, population represented, data collection method, year(s)of data collection, sex and age range, diagnostic criteria or measurement method, and sample size, as relevant.	pages:3-7
**6**	Identify and describe any categories of input data that have potentially important biases (e.g., based on characteristics listed in item 5)	page:29
**For data inputs that contribute to the analysis but were not synthesized as part of the study:**		
**7**	Describe and give sources for any other data inputs.	NA
**For all data inputs:**		
**8**	Provide all data inputs in a file format from which data can be efficiently extracted (e.g., a spreadsheet rather than a PDF), including all relevant meta-data listed in item 5. For any data inputs that cannot be shared because of ethical or legal reasons, such as third-party ownership, provide a contact name or the name of the institution that retains the right to the data.	pages:3-7
**Data analysis**		
**9**	Provide a conceptual overview of the data analysis method. A diagram may be helpful.	pages:3-7

**10**	Provide a detailed description of all steps of the analysis, including mathematical formulae. This description should cover, as relevant, data cleaning, data pre-processing, data adjustments and weighting of data sources, and mathematical or statistical model(s).	pages:3-7
**11**	Describe how candidate models were evaluated and how the final model(s)were selected	pages:3-7
**12**	Provide the results of an evaluation of model performance, if done, as well as the results of any relevant sensitivity analysis.	pages:3-7
**13**	Describe methods for calculating uncertainty of the estimates. State which sources of uncertainty were, and were not, accounted for in the uncertainty analysis.	pages:3-7
**14**	State how analytic or statistical source code used to generate estimates can be accessed	page:7
**Results and Discussion**		
**15**	Provide published estimates in a file format from which data can be efficiently extracted	NA
**16**	Report a quantitative measure of the uncertainty of the estimates (e.g. uncertainty intervals).	pages:8-25
**17**	Interpret results in light of existing evidence. If updating a previous set of estimates, describe the reasons for changes in estimates.	pages:25-29
**18**	Discuss limitations of the estimates. Include a discussion of any modelling assumptions or data limitations that affect interpretation of the estimates.	page:29

Table S2: The Socio-demographic Index (SDI) reference values from the Global Burden of Disease (GBD) data released by the Institute for Health Metrics and Evaluation (IHME) in 2021.

Location id	Location name	Lower bound	Upper bound
44637	Low SDI	0	0.46581580319161997
44636	Low-middle SDI	0.46581580319161997	0.6188294452454329
44639	Middle SDI	0.6188294452454329	0.7119746219361235
44634	High-middle SDI	0.7119746219361235	0.8102959891918925
44635	High SDI	0.8102959891918925	1

Table S3. The Socio-demographic Index (SDI) values of 204 countries/territories from the Global Burden of Disease (GBD) data released by the Institute for Health Metrics and Evaluation (IHME) in 2021.

location	SDI	SDI level
Tajikistan	0.541511187	Low-middle SDI
Kyrgyzstan	0.603979328	Low-middle SDI
Mongolia	0.617621565	Low-middle SDI
Uzbekistan	0.662621694	Middle SDI
Turkmenistan	0.682160776	Middle SDI
Azerbaijan	0.694851274	Middle SDI
Armenia	0.701833194	Middle SDI
Kazakhstan	0.725144495	High-middle SDI
Georgia	0.732473604	High-middle SDI
Albania	0.706849791	Middle SDI
Bosnia and Herzegovina	0.723077893	High-middle SDI
North Macedonia	0.750629703	High-middle SDI
Bulgaria	0.768150939	High-middle SDI
Romania	0.768453864	High-middle SDI
Hungary	0.790754768	High-middle SDI
Serbia	0.792416294	High-middle SDI
Montenegro	0.795800584	High-middle SDI
Croatia	0.798341027	High-middle SDI
Slovakia	0.81061053	High SDI
Poland	0.812042809	High SDI

location	SDI	SDI level
Czechia	0.828450433	High SDI
Slovenia	0.842430731	High SDI
Republic of Moldova	0.732214875	High-middle SDI
Ukraine	0.760773913	High-middle SDI
Belarus	0.784484711	High-middle SDI
Russian Federation	0.808536005	High-middle SDI
Latvia	0.830663516	High SDI
Estonia	0.844917787	High SDI
Lithuania	0.856484049	High SDI
Australia	0.844252814	High SDI
New Zealand	0.849442499	High SDI
Brunei Darussalam	0.810234367	High-middle SDI
Singapore	0.856097766	High SDI
Japan	0.871241813	High SDI
Republic of Korea	0.886675267	High SDI
Greenland	0.826210336	High SDI
United States of America	0.862448354	High SDI
Canada	0.87317068	High SDI
Portugal	0.744151851	High-middle SDI
Spain	0.769283698	High-middle SDI
Greece	0.791854408	High-middle SDI
Malta	0.801585034	High-middle SDI

location	SDI	SDI level
Italy	0.805773534	High-middle SDI
Israel	0.809011652	High-middle SDI
Cyprus	0.835630545	High SDI
France	0.838364875	High SDI
Belgium	0.853654016	High SDI
Austria	0.853837004	High SDI
United Kingdom	0.859000182	High SDI
Finland	0.859831368	High SDI
Andorra	0.869444113	High SDI
Ireland	0.87375385	High SDI
Iceland	0.87636168	High SDI
Luxembourg	0.884428955	High SDI
Sweden	0.886880299	High SDI
San Marino	0.888005474	High SDI
Netherlands	0.888464256	High SDI
Denmark	0.896424204	High SDI
Germany	0.902957091	High SDI
Monaco	0.908262831	High SDI
Norway	0.91613281	High SDI
Switzerland	0.933059111	High SDI
Bolivia (Plurinational State of)	0.599010799	Low-middle SDI
Ecuador	0.661017053	Middle SDI

**location**	**SDI**	**SDI level**
Peru	0.662054037	Middle SDI
Haiti	0.448278285	Low SDI
Belize	0.610229002	Low-middle SDI
Dominican Republic	0.619388201	Middle SDI
Suriname	0.633665739	Middle SDI
Saint Vincent and the Grenadines	0.637195963	Middle SDI
Guyana	0.650812335	Middle SDI
Cuba	0.668729864	Middle SDI
Grenada	0.668993028	Middle SDI
Saint Lucia	0.672509735	Middle SDI
Jamaica	0.683263064	Middle SDI
Barbados	0.746748764	High-middle SDI
Dominica	0.746967185	High-middle SDI
Antigua and Barbuda	0.749886887	High-middle SDI
Saint Kitts and Nevis	0.754987055	High-middle SDI
Trinidad and Tobago	0.768763254	High-middle SDI
Bahamas	0.805020668	High-middle SDI
Bermuda	0.821365422	High SDI
United States Virgin Islands	0.821830853	High SDI
Puerto Rico	0.825525847	High SDI
Honduras	0.513037248	Low-middle SDI
Nicaragua	0.523958472	Low-middle SDI

location	SDI	SDI level
Guatemala	0.539972424	Low-middle SDI
El Salvador	0.563775188	Low-middle SDI
Venezuela (Bolivarian Republic of)	0.596513059	Low-middle SDI
Colombia	0.655442913	Middle SDI
Mexico	0.664575304	Middle SDI
Costa Rica	0.700340477	Middle SDI
Panama	0.708864828	Middle SDI
Uruguay	0.719283445	High-middle SDI
Argentina	0.723122973	High-middle SDI
Chile	0.771514716	High-middle SDI
Paraguay	0.635718099	Middle SDI
Brazil	0.653043887	Middle SDI
Afghanistan	0.337199998	Low SDI
Yemen	0.450376375	Low SDI
Sudan	0.541949735	Low-middle SDI
Morocco	0.562698301	Low-middle SDI
Egypt	0.606787094	Low-middle SDI
Syrian Arab Republic	0.623004075	Middle SDI
Palestine	0.631011665	Middle SDI
Algeria	0.659500924	Middle SDI
Iraq	0.662626231	Middle SDI
Tunisia	0.682432216	Middle SDI

location	SDI	SDI level
Iran (Islamic Republic of)	0.697207398	Middle SDI
Turkey	0.712692673	High-middle SDI
Jordan	0.725307227	High-middle SDI
Libya	0.725771399	High-middle SDI
Lebanon	0.744746351	High-middle SDI
Bahrain	0.753043204	High-middle SDI
Oman	0.773391602	High-middle SDI
Saudi Arabia	0.815143493	High SDI
Kuwait	0.846651055	High SDI
Qatar	0.846860584	High SDI
United Arab Emirates	0.849317734	High SDI
Nepal	0.433174635	Low SDI
Bhutan	0.473062378	Low-middle SDI
Bangladesh	0.492420885	Low-middle SDI
Pakistan	0.504028689	Low-middle SDI
India	0.575401649	Low-middle SDI
Democratic People's Republic of Korea	0.5698546	Low-middle SDI
China	0.72162976	High-middle SDI
Taiwan (Province of China)	0.874747053	High SDI
Papua New Guinea	0.417797443	Low SDI
Solomon Islands	0.429360316	Low SDI
Vanuatu	0.473100706	Low-middle SDI

location	SDI	SDI level
Kiribati	0.527186583	Low-middle SDI
Marshall Islands	0.574091128	Low-middle SDI
Tuvalu	0.576620529	Low-middle SDI
Micronesia (Federated States of)	0.587534967	Low-middle SDI
Samoa	0.593392769	Low-middle SDI
Nauru	0.625177834	Middle SDI
Tonga	0.626349936	Middle SDI
Fiji	0.675051631	Middle SDI
Tokelau	0.686425621	Middle SDI
American Samoa	0.723727533	High-middle SDI
Niue	0.72622205	High-middle SDI
Palau	0.754046931	High-middle SDI
Northern Mariana Islands	0.771535213	High-middle SDI
Cook Islands	0.779109955	High-middle SDI
Guam	0.803982203	High-middle SDI
Timor-Leste	0.444667619	Low SDI
Cambodia	0.473621491	Low-middle SDI
Lao People's Democratic Republic	0.489136091	Low-middle SDI
Myanmar	0.53390084	Low-middle SDI
Viet Nam	0.627933721	Middle SDI
Maldives	0.650886627	Middle SDI
Philippines	0.651219329	Middle SDI

location	SDI	SDI level
Indonesia	0.656868336	Middle SDI
Thailand	0.682547933	Middle SDI
Sri Lanka	0.701534935	Middle SDI
Mauritius	0.718260446	High-middle SDI
Seychelles	0.730150775	High-middle SDI
Malaysia	0.742523828	High-middle SDI
Central African Republic	0.30916769	Low SDI
Democratic Republic of the Congo	0.383179849	Low SDI
Angola	0.453721949	Low SDI
Congo	0.583075236	Low-middle SDI
Gabon	0.634691393	Middle SDI
Equatorial Guinea	0.657857456	Middle SDI
Somalia	0.077688109	Low SDI
South Sudan	0.278371125	Low SDI
Burundi	0.289374365	Low SDI
Mozambique	0.326462614	Low SDI
Ethiopia	0.358823295	Low SDI
Malawi	0.384553634	Low SDI
Madagascar	0.400246943	Low SDI
Eritrea	0.403863943	Low SDI
Uganda	0.423261181	Low SDI
Rwanda	0.435588706	Low SDI

location	SDI	SDI level
United Republic of Tanzania	0.446568273	Low SDI
Comoros	0.475978688	Low-middle SDI
Djibouti	0.487958371	Low-middle SDI
Zambia	0.505948954	Low-middle SDI
Kenya	0.523768077	Low-middle SDI
Zimbabwe	0.473819486	Low-middle SDI
Lesotho	0.510393066	Low-middle SDI
Eswatini	0.585459713	Low-middle SDI
Namibia	0.617564872	Low-middle SDI
Botswana	0.642721629	Middle SDI
South Africa	0.679626598	Middle SDI
Niger	0.168072774	Low SDI
Chad	0.240436019	Low SDI
Mali	0.268579941	Low SDI
Burkina Faso	0.285118402	Low SDI
Guinea	0.336401293	Low SDI
Liberia	0.352442452	Low SDI
Guinea-Bissau	0.353109621	Low SDI
Sierra Leone	0.358665881	Low SDI
Benin	0.373486574	Low SDI
Senegal	0.408054193	Low SDI
Togo	0.408533695	Low SDI

location	SDI	SDI level
Gambia	0.40971416	Low SDI
Coted'Ivoire	0.425941883	Low SDI
Cameroon	0.479691223	Low-middle SDI
Mauritania	0.4989451	Low-middle SDI
Nigeria	0.503390833	Low-middle SDI
Sao Tome and Principe	0.505413747	Low-middle SDI
Cabo Verde	0.533534539	Low-middle SDI
Ghana	0.56493039	Low-middle SDI

Table S4: The correspondence of Global Burden of Disease (GBD) regions with 204 countries/territories from the GBD data released by the Institute for Health Metrics and Evaluation (IHME) in 2021.

Super Region	21 GBD regions	SDI value	204 countries/territories
High income	High-income Asia Pacific	0.877157409	Brunei Darussalam, Singapore, Japan, Republic of Korea
High income	High-income North America	0.86421664	Greenland, United States of America, Canada
High income	Western Europe	0.848728514	Portugal, Spain, Greece, Malta, Italy, Israel, Cyprus, France, Belgium, Austria, United Kingdom, Finland, Andorra, Ireland, Iceland, Luxembourg, Sweden, San Marino, Netherlands, Denmark, Germany, Monaco, Norway, Switzerland
High income	Australasia	0.845644432	Australia, New Zealand

**Super Region**	**21 GBD regions**	**SDI value**	**204 countries/territories**
Central Europe, eastern Europe, and central Asia	Eastern Europe	0.803414319	Republic of Moldova, Ukraine, Belarus, Russian Federation, Latvia, Estonia, Lithuania
Central Europe, eastern Europe, and central Asia	Central Europe	0.795780357	Albania, Bosnia and Herzegovina, North Macedonia, Bulgaria, Romania, Hungary, Serbia, Montenegro, Croatia, Slovakia, Poland, Czechia, Slovenia
Latin America and Caribbean	Southern Latin America	0.743029817	Uruguay, Argentina, Chile
Southeast Asia, east Asia, and Oceania	East Asia	0.722912119	China, Democratic People's Republic of Korea, Taiwan (Province of China)
Central Europe, eastern Europe, and central Asia	Central Asia	0.674963478	Tajikistan, Kyrgyzstan, Mongolia, Uzbekistan, Turkmenistan, Azerbaijan, Armenia, Kazakhstan, Georgia
Latin America and Caribbean	North Africa and Middle East	0.658716072	Afghanistan, Yemen, Sudan, Morocco, Egypt, Syrian Arab Republic, Palestine, Algeria, Iraq, Tunisia, Iran (Islamic Republic of), Turkey, Jordan, Libya, Lebanon, Bahrain, Oman, Saudi Arabia, Kuwait, Qatar, United Arab Emirates
Latin America and Caribbean	Andean Latin America	0.654007956	Bolivia (Plurinational State of), Ecuador, Peru
Southeast Asia, east Asia, and Oceania	Southeast Asia	0.64907177	Timor-Leste, Cambodia, Lao People's Democratic Republic, Myanmar, Viet Nam, Maldives, Philippines, Indonesia, Thailand, Sri Lanka, Mauritius, Seychelles, Malaysia

Super Region	21 GBD regions	SDI value	204 countries/territories
Latin America and Caribbean	Tropical Latin America	0.648941531	Paraguay, Brazil
Sub-Saharan Africa	Southern Sub-Saharan Africa	0.643347819	Zimbabwe, Lesotho, Eswatini, Namibia, Botswana, South Africa
Latin America and Caribbean	Caribbean	0.6423146	Haiti, Belize, Dominican Republic, Suriname, Saint Vincent and the Grenadines, Guyana, Cuba, Grenada, Saint Lucia, Jamaica, Barbados, Dominica, Antigua and Barbuda, Saint Kitts and Nevis, Trinidad and Tobago, Bahamas, Bermuda, United States Virgin Islands, Puerto Rico
Latin America and Caribbean	Central Latin America	0.641931122	Honduras, Nicaragua, Guatemala, El Salvador, Venezuela (Bolivarian Republic of), Colombia, Mexico, Costa Rica, Panama
South Asia	South Asia	0.559642669	Nepal, Bhutan, Bangladesh, Pakistan, India
Sub-Saharan Africa	Central Sub-Saharan Africa	0.484517732	Central African Republic, Democratic Republic of the Congo, Angola, Congo, Gabon, Equatorial Guinea
Southeast Asia, east Asia, and Oceania	Oceania	0.467359461	Papua New Guinea, Solomon Islands, Vanuatu, Kiribati, Marshall Islands, Tuvalu, Micronesia (Federated States of), Samoa, Nauru, Tonga, Fiji, Tokelau, American Samoa, Niue, Palau, Northern Mariana Islands, Cook Islands, Guam
Sub-Saharan Africa	Western Sub-Saharan Africa	0.446420999	Niger, Chad, Mali, Burkina Faso, Guinea, Liberia, Guinea-Bissau, Sierra Leone, Benin, Senegal, Togo, Coted'Ivoire, Gambia, Cameroon, Mauritania, Nigeria, Sao Tome and Principe, Cabo Verde, Ghana
Sub-Saharan Africa	Eastern Sub-Saharan Africa	0.412187942	Somalia, South Sudan, Burundi, Mozambique, Ethiopia, Malawi, Madagascar, Eritrea, Uganda, Rwanda, United Republic of Tanzania, Comoros, Djibouti, Zambia, Kenya

Table S5: Incidence of thyroid cancer in adolescents and young adults (AYAs), and their average annual percentage changes in 204 countries/territories from 1990 to 2021.

Location	ISO Code	Numbers (thousands), 1990	Age-standardised rate (per 100 000), 1990	Numbers (thousands), 2021	Age-standardised rate (per 100 000), 2021	Total percentage change in number, 1990-2021	AAPC of age-standardised rate, 1990-2021	p value
Tajikistan	TJK	0(0 - 0)	0(0 - 0)	0(0 - 0)	0(0 - 0)	0.8(-0.1 to 2.7)	-0.8(-1.16 to -0.44)	<0.001
Kyrgyzstan	KGZ	0(0 - 0)	1.7(1.1 - 2.5)	0(0 - 0.1)	1.2(0.7 - 1.8)	0.1(-0.4 to 1)	-1.5(-3.28 to 0.31)	0.10
Mongolia	MNG	0(0 - 0)	0.5(0.3 - 0.9)	0(0 - 0)	0.8(0.5 - 1.3)	2(0.7 to 3.9)	1.52(0.68 to 2.38)	<0.001
Uzbekistan	UZB	0(0 - 0)	0.2(0.1 - 0.2)	0.1(0 - 0.1)	0.4(0.2 - 0.5)	3.5(1.5 to 6.6)	2.87(2.3 to 3.45)	<0.001
Turkmenistan	TKM	0(0 - 0)	0.8(0.7 - 1)	0(0 - 0)	0.9(0.7 - 1.3)	0.7(0.2 to 1.3)	0.24(-1.88 to 2.4)	0.83
Azerbaijan	AZE	0(0 - 0)	0.5(0.3 - 0.8)	0(0 - 0)	0.5(0.3 - 0.9)	0.7(-0.2 to 2.9)	0.17(-0.43 to 0.77)	0.58
Armenia	ARM	0(0 - 0)	0.8(0.5 - 1.1)	0(0 - 0)	1.1(0.7 - 1.6)	0.2(-0.3 to 1.1)	0.86(-0.47 to 2.21)	0.20
Kazakhstan	KAZ	0.1(0.1 - 0.1)	1.8(1.4 - 2.2)	0.1(0.1 - 0.1)	1.4(1 - 1.9)	-0.1(-0.3 to 0.2)	-0.93(-3.03 to 1.22)	0.39
Georgia	GEO	0(0 - 0)	1(0.6 - 1.4)	0(0 - 0)	1.7(1.1 - 2.6)	0.1(-0.4 to 0.7)	1.52(-0.63 to 3.71)	0.17
Albania	ALB	0(0 - 0)	0.7(0.4 - 1)	0(0 - 0)	0.9(0.5 - 1.5)	-0.1(-0.5 to 0.6)	0.71(-0.03 to 1.45)	0.06
Bosnia and Herzegovina	BIH	0(0 - 0)	0.6(0.5 - 0.8)	0(0 - 0)	0.7(0.4 - 1)	-0.4(-0.6 to 0)	0.04(-0.52 to 0.6)	0.90
North Macedonia	MKD	0(0 - 0)	0.8(0.6 - 1.1)	0(0 - 0)	0.7(0.5 - 1.1)	0(-0.4 to 0.5)	-0.46(-1.31 to 0.41)	0.30
Bulgaria	BGR	0(0 - 0)	0.8(0.6 - 1.1)	0(0 - 0)	0.7(0.5 - 0.9)	-0.4(-0.6 to -0.2)	-0.85(-2.44 to 0.76)	0.30

Location	ISO Code	Numbers (thousands), 1990	Age-standardised rate (per 100 000), 1990	Numbers (thousands), 2021	Age-standardised rate (per 100 000), 2021	Total percentage change in number, 1990-2021	AAPC of age-standardised rate, 1990-2021	p value
Romania	ROU	0.1(0.1 - 0.1)	0.9(0.6 - 1.2)	0(0 - 0.1)	0.7(0.5 - 1.1)	-0.5(-0.6 to -0.2)	-0.39(-1.13 to 0.36)	0.30
Hungary	HUN	0.1(0 - 0.1)	1.7(1.2 - 2.3)	0(0 - 0)	0.9(0.6 - 1.3)	-0.6(-0.7 to -0.4)	-1.65(-2.92 to -0.36)	0.012
Serbia	SRB	0(0 - 0)	0.6(0.4 - 1.1)	0(0 - 0)	0.7(0.4 - 1.1)	0(-0.4 to 0.6)	0.3(-0.17 to 0.76)	0.22
Montenegro	MNE	0(0 - 0)	1.4(1 - 2)	0(0 - 0)	1.5(1 - 2.1)	-0.1(-0.4 to 0.3)	0.43(-0.17 to 1.04)	0.16
Croatia	HRV	0(0 - 0)	1.7(1.2 - 2.3)	0(0 - 0)	1.1(0.7 - 1.5)	-0.5(-0.7 to -0.3)	-1.65(-3.33 to 0.05)	0.06
Slovakia	SVK	0(0 - 0)	1.2(0.7 - 1.9)	0(0 - 0)	1.1(0.7 - 1.9)	-0.1(-0.5 to 0.7)	-0.06(-0.82 to 0.71)	0.88
Poland	POL	0.3(0.2 - 0.4)	1.9(1.5 - 2.3)	0.2(0.2 - 0.2)	1.3(1 - 1.7)	-0.4(-0.5 to -0.2)	-1.18(-2.33 to -0.01)	0.048
Czechia	CZE	0.1(0.1 - 0.1)	1.9(1.4 - 2.7)	0.1(0 - 0.1)	1.4(0.9 - 2.1)	-0.3(-0.6 to 0)	-0.91(-1.89 to 0.08)	0.07
Slovenia	SVN	0(0 - 0)	1.1(0.8 - 1.4)	0(0 - 0)	0.5(0.3 - 0.7)	-0.6(-0.7 to -0.5)	-2.56(-3.37 to -1.76)	<0.001
Republic of Moldova	MDA	0(0 - 0)	0.6(0.5 - 0.7)	0(0 - 0)	0.7(0.6 - 0.9)	0(-0.2 to 0.3)	0.7(-0.68 to 2.1)	0.32
Ukraine	UKR	0.2(0.1 - 0.3)	1.1(0.7 - 1.5)	0.3(0.2 - 0.4)	1.5(0.9 - 2.4)	0.2(-0.3 to 0.8)	1.15(0.37 to 1.93)	0.004
Belarus	BLR	0.1(0.1 - 0.1)	1.8(1.2 - 2.6)	0(0 - 0.1)	1.3(0.8 - 2)	-0.4(-0.6 to 0)	-1.62(-6.92 to 3.98)	0.56
Russian Federation	RUS	0.7(0.7 - 0.8)	1.1(1.1 - 1.2)	0.9(0.8 - 1)	1.6(1.5 - 1.8)	0.3(0.2 to 0.5)	0.48(-1.64 to 2.64)	0.66
Latvia	LVA	0(0 - 0)	1.8(1.2 - 2.6)	0(0 - 0)	1.2(0.8 - 1.7)	-0.6(-0.7 to -0.3)	-1.56(-3.52 to 0.45)	0.13
Estonia	EST	0(0 - 0)	2.2(1.5 - 3.2)	0(0 - 0)	1.3(0.8 - 1.8)	-0.6(-0.7 to -0.4)	-1.83(-3.8 to 0.19)	0.08
Lithuania	LTU	0(0 - 0)	2.2(1.5 - 3.2)	0(0 - 0)	1.2(0.8 - 1.7)	-0.7(-0.8 to -0.5)	-2(-3.3 to -0.69)	0.003
Australia	AUS	0.1(0.1 - 0.2)	1.6(1.1 - 2.2)	0.2(0.1 - 0.3)	2.1(1.4 - 3)	0.9(0.2 to 1.6)	1.18(-0.38 to 2.76)	0.14
New Zealand	NZL	0(0 - 0)	0.8(0.6 - 1.1)	0(0 - 0)	1.2(0.9 - 1.6)	1.1(0.5 to 2)	1.14(-0.02 to 2.31)	0.054

Location	ISO Code	Numbers (thousands), 1990	Age-standardised rate (per 100 000), 1990	Numbers (thousands), 2021	Age-standardised rate (per 100 000), 2021	Total percentage change in number, 1990-2021	AAPC of age-standardised rate, 1990-2021	p value
Brunei Darussalam	BRN	0(0 - 0)	1.2(0.6 - 2.1)	0(0 - 0)	1.4(0.8 - 2.2)	1.2(0.4 to 2.9)	0.56(0.3 to 0.81)	<0.001
Singapore	SGP	0(0 - 0)	1.4(1 - 2)	0(0 - 0)	1(0.7 - 1.5)	0.1(-0.2 to 0.5)	-0.9(-1.82 to 0.04)	0.06
Japan	JPN	0.6(0.5 - 0.8)	1.4(1.1 - 1.7)	0.6(0.5 - 0.7)	1.8(1.5 - 2)	0(-0.1 to 0.2)	1.05(0.58 to 1.52)	<0.001
Republic of Korea	KOR	0.2(0.1 - 0.4)	1.2(0.7 - 2)	0.5(0.3 - 0.8)	2.5(1.4 - 4.3)	1(0.3 to 2.1)	2.61(2.16 to 3.05)	<0.001
Greenland	GRL	0(0 - 0)	1(0.5 - 1.8)	0(0 - 0)	0.6(0.3 - 1.1)	-0.5(-0.8 to 0.1)	-1.55(-2.16 to -0.93)	<0.001
United States of America	USA	1.6(1.5 - 1.6)	1.4(1.3 - 1.5)	2.7(2.5 - 2.9)	2.3(2.1 - 2.4)	0.7(0.6 to 0.8)	1.66(0.69 to 2.63)	<0.001
Canada	CAN	0.2(0.2 - 0.3)	1.8(1.3 - 2.5)	0.2(0.2 - 0.3)	1.8(1.2 - 2.5)	0.1(-0.2 to 0.4)	0.12(-0.89 to 1.14)	0.82
Portugal	PRT	0(0 - 0.1)	1.2(0.8 - 1.7)	0(0 - 0.1)	1.2(0.8 - 1.8)	-0.1(-0.4 to 0.4)	0.01(-0.92 to 0.94)	0.98
Spain	ESP	0.2(0.1 - 0.2)	1.3(0.9 - 1.7)	0.1(0.1 - 0.2)	0.9(0.7 - 1.3)	-0.3(-0.5 to 0)	-1.06(-1.49 to -0.64)	<0.001
Greece	GRC	0(0 - 0)	1(0.8 - 1.2)	0(0 - 0)	1(0.8 - 1.3)	-0.1(-0.3 to 0.1)	0.11(-0.45 to 0.67)	0.71
Malta	MLT	0(0 - 0)	1(0.7 - 1.3)	0(0 - 0)	1.1(0.8 - 1.6)	0.2(-0.2 to 0.7)	0.57(-1.38 to 2.56)	0.57
Italy	ITA	0.5(0.4 - 0.7)	2.5(2 - 3.1)	0.4(0.3 - 0.5)	2.1(1.7 - 2.6)	-0.3(-0.4 to -0.1)	-0.53(-1.03 to -0.02)	0.043
Israel	ISR	0(0 - 0)	1.1(0.8 - 1.4)	0(0 - 0.1)	1.1(0.8 - 1.5)	0.9(0.4 to 1.6)	0.78(-1.94 to 3.58)	0.58
Cyprus	CYP	0(0 - 0)	1.1(0.6 - 2)	0(0 - 0)	1(0.6 - 1.7)	0.8(0 to 2.5)	-0.37(-1.32 to 0.58)	0.44
France	FRA	0.6(0.5 - 0.8)	2.7(2.1 - 3.6)	0.7(0.5 - 1)	3.4(2.4 - 4.8)	0.2(-0.1 to 0.5)	0.76(0.16 to 1.36)	0.013
Belgium	BEL	0.1(0 - 0.1)	1.3(1 - 1.7)	0.1(0 - 0.1)	1.4(1 - 1.8)	0(-0.2 to 0.4)	-0.08(-1.27 to 1.13)	0.90
Austria	AUT	0.1(0 - 0.1)	2(1.5 - 2.8)	0.1(0 - 0.1)	1.7(1.2 - 2.3)	-0.1(-0.4 to 0.2)	-0.6(-1.36 to 0.18)	0.13

Location	ISO Code	Numbers (thousands), 1990	Age-standardised rate (per 100 000), 1990	Numbers (thousands), 2021	Age-standardised rate (per 100 000), 2021	Total percentage change in number, 1990-2021	AAPC of age-standardised rate, 1990-2021	p value
United Kingdom	GBR	0.2(0.2 - 0.2)	0.8(0.8 - 0.9)	0.2(0.2 - 0.3)	1(0.9 - 1.1)	0.4(0.3 to 0.5)	0.56(-0.3 to 1.42)	0.20
Finland	FIN	0(0 - 0)	1.6(1.2 - 2.1)	0(0 - 0)	1.1(0.8 - 1.5)	-0.4(-0.6 to -0.1)	-1.01(-2.6 to 0.62)	0.22
Andorra	AND	0(0 - 0)	1.1(0.6 - 1.9)	0(0 - 0)	1.4(0.8 - 2.3)	0.4(-0.3 to 1.6)	0.76(0.36 to 1.16)	<0.001
Ireland	IRL	0(0 - 0)	0.9(0.7 - 1.2)	0(0 - 0)	1.1(0.8 - 1.5)	0.6(0.1 to 1.4)	0.88(-0.37 to 2.15)	0.17
Iceland	ISL	0(0 - 0)	4.1(2.9 - 5.5)	0(0 - 0)	3.1(2.2 - 4.4)	-0.1(-0.4 to 0.3)	-0.49(-1.72 to 0.75)	0.44
Luxembourg	LUX	0(0 - 0)	1.6(1.3 - 2)	0(0 - 0)	1(0.8 - 1.3)	0(-0.2 to 0.3)	-1.53(-2.81 to -0.23)	0.021
Sweden	SWE	0(0 - 0)	0.7(0.6 - 0.9)	0(0 - 0)	0.7(0.5 - 0.9)	0.1(-0.1 to 0.5)	0.15(-1.27 to 1.59)	0.83
San Marino	SMR	0(0 - 0)	2.1(1.4 - 3.3)	0(0 - 0)	2(1 - 3.5)	-0.1(-0.5 to 0.4)	-0.31(-0.59 to -0.04)	0.024
Netherlands	NLD	0.1(0 - 0.1)	0.8(0.6 - 1.1)	0.1(0 - 0.1)	1(0.7 - 1.4)	0.1(-0.2 to 0.4)	0.52(0.25 to 0.8)	<0.001
Denmark	DNK	0(0 - 0)	0.8(0.6 - 1)	0(0 - 0)	0.4(0.3 - 0.6)	-0.5(-0.6 to -0.2)	-1.69(-2.94 to -0.43)	0.009
Germany	DEU	0.6(0.5 - 0.9)	2(1.5 - 2.8)	0.5(0.3 - 0.7)	1.8(1.2 - 2.5)	-0.2(-0.4 to 0.1)	-0.46(-0.87 to -0.05)	0.029
Monaco	MCO	0(0 - 0)	1.5(0.9 - 2.4)	0(0 - 0)	2.6(1.3 - 4.4)	0.7(0 to 1.9)	1.79(1.66 to 1.91)	<0.001
Norway	NOR	0(0 - 0)	1(0.8 - 1.2)	0(0 - 0)	1(0.8 - 1.3)	0.1(-0.1 to 0.4)	-0.04(-0.66 to 0.58)	0.90
Switzerland	CHE	0(0 - 0)	1(0.7 - 1.3)	0(0 - 0)	0.5(0.4 - 0.7)	-0.4(-0.6 to -0.2)	-2.44(-4.22 to -0.63)	0.009
Bolivia (Plurinational State of)	BOL	0(0 - 0)	0.9(0.4 - 1.5)	0.1(0 - 0.1)	1.2(0.6 - 2.2)	2.1(0.8 to 5.1)	1.23(1.11 to 1.35)	<0.001
Ecuador	ECU	0(0 - 0)	0.6(0.4 - 0.9)	0.1(0.1 - 0.2)	1.6(1.1 - 2.4)	4.2(2.6 to 6.1)	3.59(3.04 to 4.14)	<0.001
Peru	PER	0.1(0 - 0.1)	0.7(0.4 - 1.1)	0.2(0.1 - 0.4)	1.4(0.8 - 2.4)	3(1.5 to 5.4)	2.53(1.21 to 3.88)	<0.001

Location	ISO Code	Numbers (thousands), 1990	Age-standardised rate (per 100 000), 1990	Numbers (thousands), 2021	Age-standardised rate (per 100 000), 2021	Total percentage change in number, 1990-2021	AAPC of age-standardised rate, 1990-2021	p value
Haiti	HTI	0(0 - 0)	0.5(0.2 - 0.9)	0(0 - 0.1)	0.6(0.3 - 1)	1.7(0.6 to 3.8)	0.68(0.15 to 1.21)	0.011
Belize	BLZ	0(0 - 0)	0.3(0.2 - 0.4)	0(0 - 0)	0.7(0.5 - 0.9)	5.9(4.5 to 7.5)	2.61(1.53 to 3.69)	<0.001
Dominican Republic	DOM	0(0 - 0)	0.5(0.3 - 0.8)	0(0 - 0.1)	0.8(0.5 - 1.4)	1.8(0.8 to 3.2)	1.67(1.25 to 2.08)	<0.001
Suriname	SUR	0(0 - 0)	0.6(0.3 - 0.9)	0(0 - 0)	0.8(0.4 - 1.4)	1.2(0.4 to 2.4)	1.07(-0.01 to 2.16)	0.052
Saint Vincent and the Grenadines	VCT	0(0 - 0)	1.1(0.8 - 1.4)	0(0 - 0)	2(1.5 - 2.6)	1(0.5 to 1.5)	2.08(1.51 to 2.65)	<0.001
Guyana	GUY	0(0 - 0)	0.4(0.2 - 0.5)	0(0 - 0)	0.9(0.5 - 1.3)	1.3(0.5 to 2.2)	2.83(1.52 to 4.16)	<0.001
Cuba	CUB	0(0 - 0.1)	1(0.7 - 1.3)	0.1(0 - 0.1)	1.4(0.9 - 1.9)	0.2(-0.1 to 0.7)	1.11(-0.02 to 2.26)	0.054
Grenada	GRD	0(0 - 0)	1.2(0.8 - 1.8)	0(0 - 0)	1.5(1 - 2.2)	0.6(0.1 to 1.3)	0.67(-0.17 to 1.52)	0.12
Saint Lucia	LCA	0(0 - 0)	1(0.8 - 1.3)	0(0 - 0)	1.6(1.2 - 2.1)	1.3(0.7 to 2)	1.37(0.31 to 2.44)	0.011
Jamaica	JAM	0(0 - 0)	0.5(0.3 - 0.7)	0(0 - 0)	1.2(0.7 - 1.9)	2.3(1.1 to 4.1)	2.99(1.78 to 4.21)	<0.001
Barbados	BRB	0(0 - 0)	0.9(0.7 - 1.1)	0(0 - 0)	1.3(0.9 - 1.9)	0.5(0.1 to 0.9)	1.44(0.63 to 2.25)	<0.001
Dominica	DMA	0(0 - 0)	0.4(0.3 - 0.6)	0(0 - 0)	0.7(0.4 - 1.2)	0.8(0.2 to 1.7)	1.81(1.21 to 2.42)	<0.001
Antigua and Barbuda	ATG	0(0 - 0)	0.9(0.7 - 1.2)	0(0 - 0)	1.2(0.9 - 1.5)	0.8(0.5 to 1.3)	0.45(-0.67 to 1.58)	0.43
Saint Kitts and Nevis	KNA	0(0 - 0)	0.6(0.5 - 0.8)	0(0 - 0)	0.5(0.3 - 0.8)	0.3(-0.1 to 0.8)	-0.63(-1.54 to 0.29)	0.18

Location	ISO Code	Numbers (thousands), 1990	Age-standardised rate (per 100 000), 1990	Numbers (thousands), 2021	Age-standardised rate (per 100 000), 2021	Total percentage change in number, 1990-2021	AAPC of age-standardised rate, 1990-2021	p value
Trinidad and Tobago	TTO	0(0 - 0)	0.7(0.5 - 0.8)	0(0 - 0)	1.4(0.9 - 1.9)	1.5(0.8 to 2.4)	2.29(1.11 to 3.49)	<0.001
Bahamas	BHS	0(0 - 0)	1(0.8 - 1.3)	0(0 - 0)	1.9(1.4 - 2.6)	1.7(0.9 to 2.6)	2.1(1.1 to 3.12)	<0.001
Bermuda	BMU	0(0 - 0)	1.2(0.8 - 1.7)	0(0 - 0)	2.2(1.5 - 3.2)	0.3(0 to 0.9)	2(0.82 to 3.21)	<0.001
United States Virgin Islands	VIR	0(0 - 0)	0.6(0.3 - 0.9)	0(0 - 0)	1(0.6 - 1.8)	0.1(-0.3 to 0.8)	2.44(2.19 to 2.7)	<0.001
Puerto Rico	PRI	0(0 - 0)	1.1(0.8 - 1.6)	0(0 - 0)	1.7(1.1 - 2.4)	0.1(-0.2 to 0.6)	1.16(-0.5 to 2.85)	0.17
Honduras	HND	0(0 - 0)	0.2(0.1 - 0.4)	0(0 - 0)	0.3(0.1 - 0.5)	2.2(0.7 to 4.9)	0.63(0.02 to 1.25)	0.044
Nicaragua	NIC	0(0 - 0)	0.4(0.2 - 0.6)	0(0 - 0)	0.7(0.4 - 1.1)	2.7(1.6 to 4.5)	1.33(0.8 to 1.87)	<0.001
Guatemala	GTM	0(0 - 0)	0.5(0.4 - 0.7)	0.1(0 - 0.1)	0.9(0.7 - 1.1)	2.9(2.1 to 3.9)	1.73(-0.13 to 3.62)	0.07
El Salvador	SLV	0(0 - 0)	0.6(0.4 - 0.8)	0(0 - 0)	1.4(0.9 - 2)	2.4(1.4 to 3.5)	2.9(1.9 to 3.92)	<0.001
Venezuela (Bolivarian Republic of)	VEN	0(0 - 0)	0.4(0.3 - 0.5)	0.1(0.1 - 0.2)	1.1(0.8 - 1.5)	2.6(1.5 to 3.9)	3.46(2.77 to 4.16)	<0.001
Colombia	COL	0.1(0.1 - 0.2)	1(0.7 - 1.4)	0.3(0.2 - 0.5)	1.6(1 - 2.4)	1.4(0.7 to 2.4)	1.56(1.21 to 1.92)	<0.001
Mexico	MEX	0.2(0.2 - 0.2)	0.5(0.5 - 0.6)	0.6(0.5 - 0.7)	1.2(1 - 1.3)	2.6(2.1 to 3)	2.52(2.21 to 2.83)	<0.001
Costa Rica	CRI	0(0 - 0)	1.4(1 - 2)	0(0 - 0)	1.4(1 - 2)	0.7(0.2 to 1.2)	-0.48(-1.74 to 0.8)	0.46
Panama	PAN	0(0 - 0)	1.6(1.1 - 2.3)	0(0 - 0)	1.3(0.8 - 1.9)	0.4(-0.1 to 1.1)	-0.77(-1.85 to 0.32)	0.16
Uruguay	URY	0(0 - 0)	1.1(0.7 - 1.6)	0(0 - 0)	1.7(1.2 - 2.4)	0.7(0.1 to 1.5)	1.89(1.51 to 2.28)	<0.001
Argentina	ARG	0.1(0.1 - 0.1)	0.8(0.6 - 1.2)	0.2(0.1 - 0.3)	1(0.7 - 1.4)	0.8(0.3 to 1.5)	0.6(-0.32 to 1.53)	0.20

Location	ISO Code	Numbers (thousands), 1990	Age-standardised rate (per 100 000), 1990	Numbers (thousands), 2021	Age-standardised rate (per 100 000), 2021	Total percentage change in number, 1990-2021	AAPC of age-standardised rate, 1990-2021	p value
Chile	CHL	0.1(0 - 0.1)	0.9(0.7 - 1.3)	0.1(0.1 - 0.2)	1.5(1.1 - 2)	1.2(0.5 to 2.3)	1.5(0.74 to 2.27)	<0.001
Paraguay	PRY	0(0 - 0)	0.7(0.4 - 1)	0(0 - 0)	1(0.5 - 1.6)	2.1(1 to 4)	1.31(0.6 to 2.02)	<0.001
Brazil	BRA	0.3(0.2 - 0.3)	0.4(0.4 - 0.5)	0.7(0.6 - 0.8)	0.8(0.7 - 0.9)	1.7(1.4 to 2)	1.69(1.46 to 1.91)	<0.001
Afghanistan	AFG	0(0 - 0.1)	0.8(0.2 - 2.5)	0.2(0.1 - 0.4)	1.7(0.6 - 3.5)	7.7(3.8 to 18.4)	2.26(2.05 to 2.48)	<0.001
Yemen	YEM	0(0 - 0)	0.3(0.1 - 0.6)	0.1(0 - 0.2)	0.7(0.4 - 1.3)	6.9(2.9 to 16.7)	3.02(1.95 to 4.1)	<0.001
Sudan	SDN	0(0 - 0.1)	0.6(0.2 - 1.6)	0.2(0.1 - 0.4)	1.4(0.6 - 2.6)	4.8(1.6 to 14.5)	2.77(2.54 to 3.01)	<0.001
Morocco	MAR	0.1(0 - 0.1)	0.8(0.5 - 1.5)	0.2(0.1 - 0.4)	1.4(0.7 - 3)	1.6(0.3 to 4.6)	1.67(1.5 to 1.83)	<0.001
Egypt	EGY	0.1(0.1 - 0.2)	0.6(0.4 - 1)	0.5(0.3 - 0.8)	1.2(0.7 - 2)	3.2(1.6 to 5.5)	2.19(2.11 to 2.28)	<0.001
Syrian Arab Republic	SYR	0(0 - 0.1)	0.7(0.3 - 1.8)	0.1(0 - 0.2)	1.8(0.9 - 3.6)	2(0.5 to 5.5)	3.34(2.82 to 3.86)	<0.001
Palestine	PSE	0(0 - 0)	1.2(0.6 - 2.2)	0(0 - 0.1)	1.8(1.1 - 3)	3.7(1.9 to 7)	1.3(0.67 to 1.93)	<0.001
Algeria	DZA	0.1(0.1 - 0.2)	1.5(0.8 - 2.6)	0.6(0.3 - 1.1)	3.1(1.6 - 5.8)	3.3(1.8 to 6)	2.39(2.17 to 2.62)	<0.001
Iraq	IRQ	0.1(0.1 - 0.2)	1.6(0.8 - 2.6)	0.5(0.3 - 0.8)	2.8(1.6 - 4.9)	3.7(1.9 to 7.3)	1.79(1.22 to 2.37)	<0.001
Tunisia	TUN	0(0 - 0.1)	1.6(0.9 - 2.8)	0.1(0.1 - 0.3)	3.1(1.6 - 5.8)	2(0.7 to 4.3)	2.17(1.85 to 2.48)	<0.001
Iran (Islamic Republic of)	IRN	0.1(0.1 - 0.2)	0.6(0.4 - 1)	0.9(0.4 - 1.3)	2.2(1 - 3.1)	6.5(3.3 to 10)	4.06(3.58 to 4.55)	<0.001
Turkey	TUR	0.3(0.2 - 0.6)	1.5(0.8 - 2.6)	0.7(0.4 - 1.2)	2.1(1.2 - 3.6)	1.1(0.3 to 2.4)	1.12(0.64 to 1.61)	<0.001
Jordan	JOR	0(0 - 0)	1.9(1 - 3.3)	0.1(0.1 - 0.2)	2.6(1.5 - 4.3)	4.4(2.5 to 8.2)	1.08(0.48 to 1.69)	<0.001
Libya	LBY	0(0 - 0.1)	2.6(1.3 - 4.9)	0.2(0.1 - 0.3)	4.8(2.3 - 9)	3.1(1.3 to 6.3)	2.13(1.25 to 3.02)	<0.001
Lebanon	LBN	0(0 - 0)	2.3(1.2 - 4.1)	0.1(0 - 0.1)	3(1.8 - 5)	2.2(0.8 to 4.6)	0.9(0.59 to 1.21)	<0.001

Location	ISO Code	Numbers (thousands), 1990	Age-standardised rate (per 100 000), 1990	Numbers (thousands), 2021	Age-standardised rate (per 100 000), 2021	Total percentage change in number, 1990-2021	AAPC of age-standardised rate, 1990-2021	p value
Bahrain	BHR	0(0 - 0)	1.3(0.7 - 2.3)	0(0 - 0)	2.4(1.3 - 4)	4.4(2.5 to 8.3)	1.96(1.07 to 2.86)	<0.001
Oman	OMN	0(0 - 0)	0.7(0.4 - 1.2)	0(0 - 0.1)	1.5(0.8 - 2.7)	6.4(3.3 to 11.2)	2.77(2.06 to 3.48)	<0.001
Saudi Arabia	SAU	0.1(0 - 0.2)	1.5(0.8 - 2.6)	1.2(0.6 - 2.1)	5.5(2.9 - 9.7)	12.1(6.4 to 24.5)	4.37(4.16 to 4.57)	<0.001
Kuwait	KWT	0(0 - 0)	2.8(2 - 3.9)	0.1(0.1 - 0.1)	2.8(1.9 - 3.9)	2(1.2 to 3)	-0.32(-6.02 to 5.73)	0.92
Qatar	QAT	0(0 - 0)	1.5(0.8 - 2.5)	0.1(0 - 0.1)	2.3(1.4 - 3.9)	11.7(6.8 to 18.7)	1.34(-0.3 to 2.99)	0.11
United Arab Emirates	ARE	0(0 - 0)	1.3(0.7 - 2.3)	0.1(0.1 - 0.2)	2.2(1.2 - 3.8)	6.3(3.7 to 10.8)	1.76(0.71 to 2.82)	<0.001
Nepal	NPL	0(0 - 0.1)	0.7(0.3 - 1.3)	0.2(0.1 - 0.4)	1.6(0.8 - 3.2)	3.2(1.4 to 7.3)	2.7(2.47 to 2.94)	<0.001
Bhutan	BTN	0(0 - 0)	0.7(0.3 - 1.3)	0(0 - 0)	1.5(0.7 - 3.4)	2.1(0.5 to 5.4)	2.4(2.13 to 2.68)	<0.001
Bangladesh	BGD	0.3(0.2 - 0.5)	0.8(0.4 - 1.4)	1.2(0.5 - 2.8)	1.7(0.7 - 4.1)	2.9(1 to 6.9)	2.74(2.39 to 3.1)	<0.001
Pakistan	PAK	0.5(0.3 - 0.8)	1.3(0.8 - 2.1)	2.7(1.6 - 4.6)	2.8(1.6 - 4.8)	4.6(2.5 to 8.4)	2.54(2.34 to 2.75)	<0.001
India	IND	2.4(1.8 - 3.4)	0.7(0.6 - 1)	9.7(7.5 - 13.2)	1.6(1.2 - 2.2)	3(1.9 to 4.7)	2.49(1.92 to 3.07)	<0.001
Democratic People's Republic of Korea	PRK	0.1(0 - 0.1)	1(0.5 - 1.9)	0.2(0.1 - 0.3)	1.6(0.8 - 2.9)	1.1(0.2 to 2.7)	1.41(1.29 to 1.53)	<0.001
China	CHN	3.1(2.4 - 4)	0.6(0.5 - 0.8)	7.5(5.9 - 10)	1.4(1.1 - 1.9)	1.4(0.7 to 2.6)	2.8(2.48 to 3.12)	<0.001
Taiwan (Province of China)	TWN	0.3(0.2 - 0.4)	3.2(2.2 - 4.4)	0.3(0.2 - 0.5)	4(2.7 - 5.7)	0.2(-0.1 to 0.6)	0.82(0.5 to 1.15)	<0.001

Location	ISO Code	Numbers (thousands), 1990	Age-standardised rate (per 100 000), 1990	Numbers (thousands), 2021	Age-standardised rate (per 100 000), 2021	Total percentage change in number, 1990-2021	AAPC of age-standardised rate, 1990-2021	p value
Papua New Guinea	PNG	0(0 - 0)	0.3(0.1 - 0.6)	0(0 - 0)	0.4(0.2 - 0.8)	3(1.5 to 5.6)	1.15(0.85 to 1.45)	<0.001
Solomon Islands	SLB	0(0 - 0)	0.3(0.1 - 0.5)	0(0 - 0)	0.6(0.3 - 1)	4.4(2.3 to 8.2)	2.61(2.32 to 2.9)	<0.001
Vanuatu	VUT	0(0 - 0)	0.3(0.2 - 0.5)	0(0 - 0)	0.5(0.3 - 0.9)	3(1.4 to 5.7)	1.94(1.54 to 2.34)	<0.001
Kiribati	KIR	0(0 - 0)	0(0 - 0)	0(0 - 0)	0(0 - 0)	1.6(0.5 to 3.5)	1.2(1.08 to 1.33)	<0.001
Marshall Islands	MHL	0(0 - 0)	0.3(0.2 - 0.6)	0(0 - 0)	0.7(0.4 - 1.3)	2.3(0.9 to 4.3)	2.36(2.22 to 2.5)	<0.001
Tuvalu	TUV	0(0 - 0)	0.4(0.2 - 0.6)	0(0 - 0)	0.7(0.4 - 1.2)	1.6(0.6 to 3.6)	2.17(2.06 to 2.28)	<0.001
Micronesia (Federated States of)	FSM	0(0 - 0)	0.4(0.2 - 0.7)	0(0 - 0)	0.8(0.4 - 1.4)	1(0.2 to 2.5)	2.02(1.96 to 2.08)	<0.001
Samoa	WSM	0(0 - 0)	1.4(0.7 - 2.5)	0(0 - 0)	2.7(1.2 - 5.4)	1.7(0.5 to 3.7)	2.2(2.02 to 2.38)	<0.001
Nauru	NRU	0(0 - 0)	0.6(0.3 - 1)	0(0 - 0)	1.1(0.5 - 2)	1.1(0.3 to 2.5)	1.96(1.78 to 2.13)	<0.001
Tonga	TON	0(0 - 0)	0.4(0.2 - 0.6)	0(0 - 0)	0.7(0.4 - 1.3)	1.4(0.4 to 3.1)	2.15(1.87 to 2.42)	<0.001
Fiji	FJI	0(0 - 0)	1.3(0.7 - 2.2)	0(0 - 0)	1.7(0.9 - 3)	0.6(-0.1 to 1.7)	0.87(0.25 to 1.49)	0.006
Tokelau	TKL	0(0 - 0)	0.4(0.2 - 0.8)	0(0 - 0)	1.3(0.8 - 2.3)	1.7(0.6 to 3.9)	3.8(3.15 to 4.47)	<0.001
American Samoa	ASM	0(0 - 0)	0.7(0.4 - 1.3)	0(0 - 0)	1.5(0.8 - 2.5)	0.9(0.1 to 2.6)	2.41(1.96 to 2.86)	<0.001
Niue	NIU	0(0 - 0)	0.5(0.3 - 0.9)	0(0 - 0)	1.4(0.8 - 2.3)	1(0.2 to 2.7)	3.22(2.88 to 3.56)	<0.001

Location	ISO Code	Numbers (thousands), 1990	Age-standardised rate (per 100 000), 1990	Numbers (thousands), 2021	Age-standardised rate (per 100 000), 2021	Total percentage change in number, 1990-2021	AAPC of age-standardised rate, 1990-2021	p value
Palau	PLW	0(0 - 0)	0.6(0.3 - 1.1)	0(0 - 0)	1.2(0.6 - 2.1)	0.9(0.1 to 2)	2.04(1.89 to 2.2)	<0.001
Northern Mariana Islands	MNP	0(0 - 0)	0.4(0.2 - 0.8)	0(0 - 0)	0.7(0.4 - 1.2)	0.2(-0.3 to 1.3)	1.56(0.57 to 2.56)	0.002
Cook Islands	COK	0(0 - 0)	0.9(0.5 - 1.5)	0(0 - 0)	1.3(0.7 - 2.4)	0.3(-0.3 to 1.5)	1.56(0.76 to 2.36)	<0.001
Guam	GUM	0(0 - 0)	0.3(0.2 - 0.7)	0(0 - 0)	1(0.7 - 1.4)	1.8(0.3 to 3.4)	4.17(2.4 to 5.96)	<0.001
Timor-Leste	TLS	0(0 - 0)	0.4(0.2 - 0.7)	0(0 - 0)	0.7(0.4 - 1.3)	1.9(0.6 to 4.3)	1.93(1.03 to 2.85)	<0.001
Cambodia	KHM	0(0 - 0)	0.7(0.3 - 1.3)	0.1(0.1 - 0.2)	1.4(0.7 - 2.7)	3.3(1.4 to 6.5)	2.28(2.09 to 2.47)	<0.001
Lao People's Democratic Republic	LAO	0(0 - 0)	0.6(0.3 - 1.2)	0(0 - 0.1)	1.1(0.5 - 2.1)	3.1(1.3 to 6.6)	1.94(1.76 to 2.12)	<0.001
Myanmar	MMR	0.1(0.1 - 0.3)	0.9(0.4 - 1.6)	0.3(0.1 - 0.5)	1.1(0.6 - 2.1)	0.9(0 to 2.5)	0.97(0.86 to 1.07)	<0.001
Viet Nam	VNM	0.4(0.2 - 0.7)	1.4(0.7 - 2.7)	1.8(0.8 - 3.4)	4.3(1.9 - 8)	3.8(2 to 6.8)	3.57(3.44 to 3.69)	<0.001
Maldives	MDV	0(0 - 0)	0.4(0.1 - 0.7)	0(0 - 0)	0.5(0.3 - 0.8)	4.8(2.1 to 14.1)	0.66(0.04 to 1.29)	0.036
Philippines	PHL	0.4(0.3 - 0.5)	1.6(1.1 - 2.1)	0.8(0.6 - 1.3)	1.8(1.3 - 2.8)	1.3(0.7 to 2.3)	0.5(0.19 to 0.81)	0.002
Indonesia	IDN	0.5(0.3 - 0.7)	0.7(0.4 - 1)	1.2(0.8 - 1.8)	1(0.7 - 1.5)	1.4(0.5 to 2.8)	1.22(1.01 to 1.43)	<0.001
Thailand	THA	0.3(0.2 - 0.5)	1.1(0.7 - 1.9)	0.6(0.3 - 1)	2.5(1.4 - 4.3)	1.1(0.2 to 2.4)	2.55(1.73 to 3.37)	<0.001
Sri Lanka	LKA	0.1(0 - 0.1)	1(0.6 - 1.7)	0.2(0.1 - 0.3)	1.9(0.9 - 3.5)	1.1(0.2 to 2.6)	2.02(1.11 to 2.94)	<0.001
Mauritius	MUS	0(0 - 0)	0.6(0.5 - 0.8)	0(0 - 0)	0.7(0.5 - 0.9)	0.1(-0.1 to 0.3)	-0.14(-3.31 to 3.12)	0.93
Seychelles	SYC	0(0 - 0)	0.4(0.2 - 0.6)	0(0 - 0)	0.6(0.3 - 0.9)	1.2(0.4 to 2.4)	1.23(0.99 to 1.47)	<0.001
Malaysia	MYS	0.1(0 - 0.2)	1.3(0.7 - 2.4)	0.3(0.2 - 0.6)	2.4(1.3 - 4.2)	2.6(1.3 to 4.4)	1.74(1.5 to 1.98)	<0.001

Location	ISO Code	Numbers (thousands), 1990	Age-standardised rate (per 100 000), 1990	Numbers (thousands), 2021	Age-standardised rate (per 100 000), 2021	Total percentage change in number, 1990-2021	AAPC of age-standardised rate, 1990-2021	p value
Central African Republic	CAF	0(0 - 0)	0.2(0.1 - 0.3)	0(0 - 0)	0.2(0.1 - 0.3)	1.2(0.3 to 2.3)	0.02(-0.17 to 0.21)	0.83
Democratic Republic of the Congo	COD	0(0 - 0)	0.2(0.1 - 0.3)	0.1(0 - 0.1)	0.2(0.1 - 0.4)	2.1(0.9 to 4.2)	0.61(0.34 to 0.87)	<0.001
Angola	AGO	0(0 - 0)	0.2(0.1 - 0.3)	0(0 - 0.1)	0.3(0.1 - 0.5)	4.1(2.1 to 7.3)	1.65(1.13 to 2.17)	<0.001
Congo	COG	0(0 - 0)	0.2(0.1 - 0.4)	0(0 - 0)	0.4(0.2 - 0.8)	3.6(1.6 to 6.7)	1.95(1.15 to 2.76)	<0.001
Gabon	GAB	0(0 - 0)	0.3(0.1 - 0.4)	0(0 - 0)	0.4(0.2 - 0.8)	2.1(0.7 to 4.4)	1.43(1.15 to 1.7)	<0.001
Equatorial Guinea	GNQ	0(0 - 0)	0.2(0.1 - 0.3)	0(0 - 0)	0.4(0.2 - 0.8)	10.5(5 to 20.5)	3.1(2.32 to 3.89)	<0.001
Somalia	SOM	0(0 - 0)	0.7(0.3 - 1.2)	0.1(0 - 0.1)	0.8(0.4 - 1.5)	2.3(1.1 to 4.4)	0.61(0.48 to 0.75)	<0.001
South Sudan	SSD	0(0 - 0)	0.7(0.3 - 1.3)	0(0 - 0.1)	1.3(0.6 - 2.6)	2(0.7 to 4.5)	2.26(1.85 to 2.68)	<0.001
Burundi	BDI	0(0 - 0)	1(0.5 - 1.9)	0.1(0 - 0.1)	1.1(0.5 - 2.1)	1.7(0.5 to 4.8)	0.14(-0.03 to 0.32)	0.11
Mozambique	MOZ	0(0 - 0.1)	0.8(0.4 - 1.7)	0.1(0.1 - 0.3)	1.3(0.6 - 3)	3.2(1.4 to 5.9)	1.74(1.42 to 2.05)	<0.001
Ethiopia	ETH	0.4(0.2 - 0.6)	2.2(1.3 - 3.6)	1.3(0.7 - 2.4)	3(1.7 - 5.7)	2.4(0.9 to 6.1)	0.92(0.79 to 1.05)	<0.001
Malawi	MWI	0(0 - 0.1)	0.9(0.4 - 1.7)	0.1(0 - 0.2)	1.5(0.7 - 3.3)	2.7(0.9 to 6.1)	1.82(1.45 to 2.19)	<0.001
Madagascar	MDG	0(0 - 0.1)	0.9(0.5 - 1.6)	0.1(0.1 - 0.3)	1.3(0.6 - 2.4)	2.7(1.1 to 5.1)	1.07(0.97 to 1.18)	<0.001
Eritrea	ERI	0(0 - 0)	0.7(0.4 - 1.2)	0(0 - 0.1)	1.1(0.5 - 2.3)	2.7(1.1 to 5.8)	1.55(1.36 to 1.75)	<0.001
Uganda	UGA	0(0 - 0.1)	0.8(0.4 - 1.5)	0.4(0.2 - 0.7)	2.5(1.3 - 4.6)	7.3(3.7 to 13.6)	3.57(2.89 to 4.26)	<0.001
Rwanda	RWA	0(0 - 0.1)	1.3(0.7 - 2.3)	0.1(0 - 0.2)	1.5(0.7 - 3.1)	1.3(0.2 to 4.4)	0.35(0.19 to 0.5)	<0.001

Location	ISO Code	Numbers (thousands), 1990	Age-standardised rate (per 100 000), 1990	Numbers (thousands), 2021	Age-standardised rate (per 100 000), 2021	Total percentage change in number, 1990-2021	AAPC of age-standardised rate, 1990-2021	p value
United Republic of Tanzania	TZA	0.1(0 - 0.2)	1(0.5 - 1.9)	0.3(0.2 - 0.6)	1.5(0.7 - 2.9)	2.6(1 to 5.4)	1.25(1.08 to 1.42)	<0.001
Comoros	COM	0(0 - 0)	1(0.4 - 2.1)	0(0 - 0)	1.5(0.7 - 3.2)	2(0.6 to 5)	0.92(-1.5 to 3.41)	0.46
Djibouti	DJI	0(0 - 0)	0.6(0.3 - 1.3)	0(0 - 0)	1.1(0.5 - 2.5)	5.1(2.1 to 10.2)	1.98(1.77 to 2.19)	<0.001
Zambia	ZMB	0(0 - 0.1)	1.1(0.6 - 1.9)	0.2(0.1 - 0.5)	2.3(0.8 - 6)	4.8(1.4 to 13.8)	2.37(2.13 to 2.6)	<0.001
Kenya	KEN	0(0 - 0)	0.3(0.2 - 0.5)	0.1(0.1 - 0.2)	0.5(0.3 - 1)	3.6(2 to 6.3)	1.96(1.54 to 2.38)	<0.001
Zimbabwe	ZWE	0(0 - 0.1)	0.9(0.5 - 1.6)	0.1(0.1 - 0.2)	1.9(0.9 - 3.8)	2.7(1.1 to 5.4)	2.54(1.78 to 3.31)	<0.001
Lesotho	LSO	0(0 - 0)	0.2(0.1 - 0.4)	0(0 - 0)	0.4(0.2 - 0.7)	2.2(0.7 to 4.8)	2.28(1.67 to 2.9)	<0.001
Eswatini	SWZ	0(0 - 0)	0.3(0.2 - 0.6)	0(0 - 0)	0.6(0.2 - 1.1)	2.1(0.6 to 4.5)	1.61(1.44 to 1.79)	<0.001
Namibia	NAM	0(0 - 0)	0.4(0.2 - 0.7)	0(0 - 0)	0.7(0.3 - 1.5)	3(1.3 to 5.9)	2.3(1.77 to 2.84)	<0.001
Botswana	BWA	0(0 - 0)	0.3(0.1 - 0.6)	0(0 - 0)	0.4(0.2 - 0.8)	2.1(0.8 to 4.1)	0.64(-0.25 to 1.53)	0.16
South Africa	ZAF	0.1(0.1 - 0.1)	0.6(0.5 - 0.9)	0.1(0.1 - 0.2)	0.5(0.4 - 0.9)	0.7(0.3 to 1.6)	-0.3(-1.33 to 0.74)	0.57
Niger	NER	0(0 - 0)	0.1(0.1 - 0.3)	0(0 - 0)	0.1(0.1 - 0.2)	1.7(0.4 to 3.7)	-0.22(-0.54 to 0.1)	0.18
Chad	TCD	0(0 - 0)	0.1(0.1 - 0.3)	0(0 - 0)	0.2(0.1 - 0.3)	2.4(0.7 to 5.1)	0.65(0.5 to 0.81)	<0.001
Mali	MLI	0(0 - 0)	0.8(0.4 - 1.4)	0.1(0 - 0.1)	0.9(0.4 - 1.9)	2.4(0.8 to 4.8)	0.58(0.32 to 0.83)	<0.001
Burkina Faso	BFA	0(0 - 0)	0.2(0.1 - 0.4)	0(0 - 0)	0.2(0.1 - 0.4)	1.7(0.5 to 3.8)	-0.05(-0.38 to 0.29)	0.78
Guinea	GIN	0(0 - 0)	0.3(0.2 - 0.5)	0(0 - 0)	0.4(0.2 - 0.9)	2.7(1.2 to 5.5)	1.26(1.06 to 1.47)	<0.001
Liberia	LBR	0(0 - 0)	0.2(0.1 - 0.3)	0(0 - 0)	0.3(0.1 - 0.5)	2.9(1 to 6)	1.63(1.28 to 1.98)	<0.001
Guinea-Bissau	GNB	0(0 - 0)	0.2(0.1 - 0.5)	0(0 - 0)	0.3(0.1 - 0.5)	1.5(0.3 to 3.5)	0.19(-0.03 to 0.41)	0.10
Sierra Leone	SLE	0(0 - 0)	0.1(0.1 - 0.3)	0(0 - 0)	0.2(0.1 - 0.4)	2.3(0.7 to 5)	1.27(0.82 to 1.72)	<0.001

Location	ISO Code	Numbers (thousands), 1990	Age-standardised rate (per 100 000), 1990	Numbers (thousands), 2021	Age-standardised rate (per 100 000), 2021	Total percentage change in number, 1990-2021	AAPC of age-standardised rate, 1990-2021	p value
Benin	BEN	0(0 - 0)	0.1(0.1 - 0.3)	0(0 - 0)	0.2(0.1 - 0.3)	2.4(1 to 4.8)	0.53(0.28 to 0.78)	<0.001
Senegal	SEN	0(0 - 0)	0.2(0.1 - 0.4)	0(0 - 0)	0.2(0.1 - 0.4)	1.6(0.4 to 3.7)	0.2(-0.49 to 0.89)	0.57
Togo	TGO	0(0 - 0)	0.2(0.1 - 0.4)	0(0 - 0)	0.2(0.1 - 0.5)	1.9(0.7 to 3.8)	0.36(0.08 to 0.63)	0.011
Gambia	GMB	0(0 - 0)	0.2(0.1 - 0.4)	0(0 - 0)	0.4(0.2 - 0.8)	3.7(1.6 to 7.2)	1.83(0.65 to 3.02)	0.002
Coted'Ivoire	CIV	0(0 - 0)	0.4(0.2 - 0.8)	0.1(0 - 0.2)	0.7(0.3 - 1.5)	3.4(1.4 to 6.9)	1.87(1.42 to 2.34)	<0.001
Cameroon	CMR	0(0 - 0)	0.2(0.1 - 0.5)	0(0 - 0.1)	0.3(0.1 - 0.5)	2.8(1.1 to 5.5)	0.32(0.11 to 0.54)	0.002
Mauritania	MRT	0(0 - 0)	0.1(0.1 - 0.3)	0(0 - 0)	0.2(0.1 - 0.5)	2.2(0.6 to 5.5)	1.31(0.98 to 1.64)	<0.001
Nigeria	NGA	0(0 - 0)	0(0 - 0.1)	0.1(0 - 0.1)	0.1(0 - 0.1)	3.6(1.4 to 7.4)	1.92(1.63 to 2.22)	<0.001
Sao Tome and Principe	STP	0(0 - 0)	0.1(0 - 0.2)	0(0 - 0)	0.2(0 - 0.4)	4.2(2.1 to 8.9)	2.3(1.21 to 3.4)	<0.001
Cabo Verde	CPV	0(0 - 0)	0.1(0 - 0.2)	0(0 - 0)	0.4(0.1 - 0.9)	15.2(0.8 to 45.7)	5.99(5.34 to 6.64)	<0.001
Ghana	GHA	0(0 - 0)	0(0 - 0)	0(0 - 0)	0(0 - 0.1)	3.6(1.8 to 6.6)	1.8(1.61 to 1.98)	<0.001

Data in parentheses are 95% uncertainty intervals for numbers, total percentage change, and 95% CIs for age-standardised rates, and AAPCs. AAPC=average annual percentage change.

Table S6: Prevalence of thyroid cancer in adolescents and young adults (AYAs), and their average annual percentage changes in 204 countries/territories from 1990 to 2021.

Location	ISO Code	Numbers (thousands), 1990	Age-standardised rate (per 100 000), 1990	Numbers (thousands), 2021	Age-standardised rate (per 100 000), 2021	Total percentage change in number, 1990-2021	AAPC of age-standardised rate, 1990-2021	p value
Tajikistan	TJK	0(0 - 0)	0.1(0 - 0.1)	0(0 - 0)	0.1(0 - 0.1)	0.8(-0.1 to 2.7)	-0.78(-1.14 to -0.41)	<0.001
Kyrgyzstan	KGZ	0.3(0.2 - 0.4)	15.4(10 - 22.9)	0.3(0.2 - 0.5)	11.1(6.8 - 16.6)	0.1(-0.4 to 1)	-1.47(-3.26 to 0.34)	0.11
Mongolia	MNG	0(0 - 0.1)	4.5(2.6 - 7.7)	0.1(0.1 - 0.2)	7.3(4.4 - 11.6)	2(0.7 to 4)	1.6(0.77 to 2.45)	<0.001
Uzbekistan	UZB	0.1(0.1 - 0.2)	1.3(0.9 - 2)	0.5(0.3 - 0.7)	3.2(2.1 - 4.7)	3.5(1.5 to 6.7)	2.89(2.32 to 3.47)	<0.001
Turkmenistan	TKM	0.1(0.1 - 0.1)	7.5(6 - 9.3)	0.2(0.1 - 0.2)	8.3(6 - 11.6)	0.7(0.2 to 1.3)	0.27(-1.85 to 2.43)	0.81
Azerbaijan	AZE	0.1(0.1 - 0.2)	4.2(2.3 - 7.2)	0.2(0.1 - 0.4)	4.6(2.4 - 8.1)	0.7(-0.2 to 3)	0.2(-0.39 to 0.8)	0.51
Armenia	ARM	0.1(0.1 - 0.2)	7.1(4.6 - 10.4)	0.1(0.1 - 0.2)	10.2(6.8 - 14.7)	0.2(-0.3 to 1.1)	0.89(-0.44 to 2.23)	0.19
Kazakhstan	KAZ	1.1(0.9 - 1.3)	16(12.8 - 19.5)	1(0.7 - 1.3)	12.8(9.4 - 16.9)	-0.1(-0.3 to 0.2)	-0.89(-3 to 1.27)	0.42
Georgia	GEO	0.2(0.1 - 0.3)	8.6(5.6 - 12.7)	0.2(0.1 - 0.3)	15.6(10.2 - 23.3)	0.1(-0.4 to 0.8)	1.54(-0.6 to 3.72)	0.16
Albania	ALB	0.1(0.1 - 0.1)	6.2(4 - 9.3)	0.1(0 - 0.1)	8.1(4.5 - 13.8)	-0.1(-0.5 to 0.6)	0.76(0.13 to 1.4)	0.018
Bosnia and Herzegovina	BIH	0.1(0.1 - 0.1)	5.6(4.1 - 7.5)	0.1(0 - 0.1)	5.9(3.8 - 9.4)	-0.4(-0.6 to 0)	0.08(-0.47 to 0.63)	0.79
North Macedonia	MKD	0.1(0 - 0.1)	7.1(5.1 - 9.6)	0.1(0 - 0.1)	6.3(4.2 - 9.7)	0(-0.4 to 0.5)	-0.43(-1.28 to 0.43)	0.33
Bulgaria	BGR	0.2(0.2 - 0.3)	7.2(5.5 - 9.6)	0.1(0.1 - 0.2)	5.9(4.2 - 8.4)	-0.4(-0.6 to -0.2)	-0.82(-2.43 to 0.8)	0.32
Romania	ROU	0.7(0.5 - 1)	8(5.6 - 11.2)	0.4(0.3 - 0.6)	6.6(4.3 - 9.6)	-0.4(-0.6 to -0.2)	-0.35(-1.1 to 0.4)	0.36
Hungary	HUN	0.6(0.4 - 0.8)	15(10.6 - 20.4)	0.3(0.2 - 0.4)	8.6(5.8 - 12.3)	-0.6(-0.7 to -0.4)	-1.61(-2.88 to -0.33)	0.014
Serbia	SRB	0.2(0.1 - 0.4)	5.7(3.4 - 9.5)	0.2(0.1 - 0.3)	6.2(3.6 - 10.3)	0(-0.4 to 0.6)	0.34(-0.13 to 0.8)	0.16

Location	ISO Code	Numbers (thousands), 1990	Age-standardised rate (per 100 000), 1990	Numbers (thousands), 2021	Age-standardised rate (per 100 000), 2021	Total percentage change in number, 1990-2021	AAPC of age-standardised rate, 1990-2021	p value
Montenegro	MNE	0(0 - 0)	13(9.3 - 18.3)	0(0 - 0)	13.5(9.3 - 19.3)	-0.1(-0.4 to 0.3)	0.44(-0.17 to 1.05)	0.16
Croatia	HRV	0.3(0.2 - 0.4)	15.1(10.9 - 21.1)	0.1(0.1 - 0.2)	9.7(6.6 - 13.9)	-0.5(-0.7 to -0.3)	-1.63(-3.31 to 0.08)	0.06
Slovakia	SVK	0.2(0.1 - 0.4)	10.7(6.5 - 17.3)	0.2(0.1 - 0.4)	10.5(6 - 17.3)	-0.1(-0.5 to 0.7)	-0.03(-0.79 to 0.73)	0.93
Poland	POL	2.8(2.2 - 3.4)	17(13.8 - 21)	1.8(1.4 - 2.3)	12.1(9.3 - 15.4)	-0.4(-0.5 to -0.2)	-1.14(-2.28 to 0.02)	0.06
Czechia	CZE	0.7(0.5 - 1)	17.5(12.4 - 24.3)	0.5(0.3 - 0.7)	13.2(8.6 - 19.3)	-0.3(-0.6 to 0.1)	-0.89(-1.87 to 0.1)	0.08
Slovenia	SVN	0.1(0.1 - 0.1)	9.6(7.1 - 12.8)	0(0 - 0)	4.5(3.1 - 6.4)	-0.6(-0.7 to -0.5)	-2.54(-3.34 to -1.73)	<0.001
Republic of Moldova	MDA	0.1(0.1 - 0.1)	5.2(4.2 - 6.5)	0.1(0.1 - 0.1)	6.6(5.2 - 8.5)	0(-0.2 to 0.3)	0.73(-0.65 to 2.13)	0.30
Ukraine	UKR	2(1.3 - 2.8)	9.8(6.7 - 13.8)	2.3(1.4 - 3.7)	13.8(8.4 - 22)	0.2(-0.3 to 0.8)	1.16(0.38 to 1.94)	0.003
Belarus	BLR	0.7(0.5 - 1)	16.4(11 - 23.8)	0.4(0.3 - 0.6)	12(7.6 - 17.8)	-0.4(-0.6 to 0)	-1.61(-6.9 to 3.98)	0.57
Russian Federation	RUS	6.4(6.1 - 6.8)	10.1(9.6 - 10.7)	8.5(7.5 - 9.4)	14.8(13.2 - 16.4)	0.3(0.2 to 0.5)	0.49(-1.62 to 2.64)	0.65
Latvia	LVA	0.2(0.1 - 0.2)	16.1(10.8 - 23.5)	0.1(0 - 0.1)	10.7(7 - 15.5)	-0.6(-0.7 to -0.3)	-1.54(-3.51 to 0.47)	0.13
Estonia	EST	0.1(0.1 - 0.2)	19.7(13.2 - 28.7)	0.1(0 - 0.1)	11.4(7.7 - 16.4)	-0.6(-0.7 to -0.4)	-1.81(-3.78 to 0.2)	0.08
Lithuania	LTU	0.3(0.2 - 0.4)	20.4(13.8 - 29.4)	0.1(0.1 - 0.1)	10.9(7.5 - 15.3)	-0.7(-0.8 to -0.5)	-2(-3.3 to -0.68)	0.003
Australia	AUS	1(0.7 - 1.4)	14.4(10.2 - 19.7)	1.9(1.3 - 2.7)	19.4(13 - 27.6)	0.9(0.2 to 1.6)	1.19(-0.36 to 2.76)	0.13
New Zealand	NZL	0.1(0.1 - 0.1)	7(5.2 - 9.3)	0.2(0.1 - 0.3)	10.8(7.9 - 14.6)	1.1(0.5 to 2)	1.19(0.04 to 2.36)	0.043
Brunei Darussalam	BRN	0(0 - 0)	10.5(5.1 - 19.3)	0(0 - 0)	12.4(7 - 20.5)	1.2(0.4 to 3)	0.58(0.33 to 0.84)	<0.001
Singapore	SGP	0.2(0.1 - 0.3)	12.9(9 - 17.9)	0.2(0.1 - 0.3)	9.3(6.5 - 13.4)	0.1(-0.2 to 0.5)	-0.88(-1.8 to 0.06)	0.07

Location	ISO Code	Numbers (thousands), 1990	Age-standardised rate (per 100 000), 1990	Numbers (thousands), 2021	Age-standardised rate (per 100 000), 2021	Total percentage change in number, 1990-2021	AAPC of age-standardised rate, 1990-2021	p value
Japan	JPN	5.7(4.6 - 7.1)	12.4(10 - 15.5)	5.9(4.9 - 6.7)	16.4(13.6 - 18.6)	0(-0.1 to 0.2)	1.06(0.59 to 1.53)	<0.001
Republic of Korea	KOR	2.2(1.3 - 3.7)	10.6(6.2 - 18.3)	4.3(2.4 - 7.2)	23.3(13.1 - 39.4)	1(0.3 to 2.2)	2.64(2.19 to 3.09)	<0.001
Greenland	GRL	0(0 - 0)	9(4.1 - 16.6)	0(0 - 0)	5.6(2.8 - 10)	-0.5(-0.8 to 0.2)	-1.5(-2.12 to -0.88)	<0.001
United States of America	USA	14.2(13.4 - 14.9)	12.9(12.2 - 13.6)	24.3(22.8 - 26.1)	20.7(19.4 - 22.2)	0.7(0.6 to 0.8)	1.66(0.69 to 2.64)	<0.001
Canada	CAN	2(1.4 - 2.8)	16.7(11.5 - 23.2)	2.1(1.4 - 2.9)	16.2(11.1 - 22.5)	0.1(-0.2 to 0.4)	0.13(-0.88 to 1.15)	0.81
Portugal	PRT	0.4(0.3 - 0.6)	10.8(7.4 - 15.2)	0.4(0.2 - 0.6)	11.4(7.5 - 16.8)	-0.1(-0.4 to 0.5)	0.04(-0.88 to 0.98)	0.93
Spain	ESP	1.7(1.2 - 2.3)	11.8(8.6 - 15.7)	1.2(0.8 - 1.7)	8.7(6 - 12.2)	-0.3(-0.5 to 0)	-1.03(-1.46 to -0.61)	<0.001
Greece	GRC	0.3(0.3 - 0.4)	8.9(7.2 - 10.9)	0.3(0.2 - 0.4)	9.5(7.5 - 11.8)	-0.1(-0.3 to 0.1)	0.12(-0.44 to 0.68)	0.68
Malta	MLT	0(0 - 0)	8.9(6.3 - 12.3)	0(0 - 0)	10.2(7 - 14.4)	0.2(-0.2 to 0.7)	0.58(-1.37 to 2.58)	0.56
Italy	ITA	4.8(3.9 - 5.9)	22.7(18.4 - 27.9)	3.5(2.8 - 4.2)	19.6(15.8 - 23.9)	-0.3(-0.4 to -0.1)	-0.51(-1.02 to 0)	0.051
Israel	ISR	0.2(0.1 - 0.2)	9.6(6.9 - 12.9)	0.3(0.3 - 0.5)	10.4(7.5 - 14.1)	0.9(0.4 to 1.6)	0.8(-1.92 to 3.6)	0.57
Cyprus	CYP	0(0 - 0.1)	10.4(5.8 - 18)	0.1(0 - 0.1)	9.5(5.6 - 15.9)	0.8(0 to 2.6)	-0.35(-1.29 to 0.6)	0.47
France	FRA	5.7(4.3 - 7.6)	24.9(18.8 - 32.8)	6.6(4.7 - 9.3)	31.4(22 - 43.8)	0.2(-0.1 to 0.5)	0.78(0.18 to 1.39)	0.011
Belgium	BEL	0.5(0.3 - 0.6)	11.8(8.8 - 15.8)	0.5(0.4 - 0.6)	12.4(9.2 - 16.6)	0(-0.2 to 0.4)	-0.06(-1.25 to 1.15)	0.93
Austria	AUT	0.6(0.4 - 0.8)	18.6(13.4 - 25.2)	0.5(0.3 - 0.7)	15.4(10.8 - 21.2)	-0.1(-0.4 to 0.2)	-0.58(-1.35 to 0.2)	0.14
United Kingdom	GBR	1.6(1.5 - 1.7)	7.6(7.1 - 8.2)	2.2(2 - 2.4)	9.2(8.5 - 9.9)	0.4(0.3 to 0.5)	0.57(-0.28 to 1.43)	0.19
Finland	FIN	0.3(0.2 - 0.4)	14.5(10.7 - 19.4)	0.2(0.1 - 0.2)	9.8(6.9 - 13.6)	-0.4(-0.6 to -0.1)	-0.98(-2.58 to 0.64)	0.23
Andorra	AND	0(0 - 0)	10.4(5.9 - 17)	0(0 - 0)	12.9(7 - 21)	0.4(-0.3 to 1.6)	0.78(0.38 to 1.17)	<0.001

Location	ISO Code	Numbers (thousands), 1990	Age-standardised rate (per 100 000), 1990	Numbers (thousands), 2021	Age-standardised rate (per 100 000), 2021	Total percentage change in number, 1990-2021	AAPC of age-standardised rate, 1990-2021	p value
Ireland	IRL	0.1(0.1 - 0.1)	8.1(6 - 10.9)	0.2(0.1 - 0.2)	10.2(7.2 - 14.2)	0.6(0.1 to 1.5)	0.91(-0.35 to 2.18)	0.16
Iceland	ISL	0(0 - 0.1)	37.2(26.9 - 50.5)	0(0 - 0.1)	28.8(20 - 40.5)	-0.1(-0.3 to 0.3)	-0.48(-1.71 to 0.76)	0.45
Luxembourg	LUX	0(0 - 0)	14.8(11.8 - 18.2)	0(0 - 0)	9.6(7.5 - 12.1)	0(-0.2 to 0.3)	-1.49(-2.78 to -0.2)	0.024
Sweden	SWE	0.2(0.2 - 0.3)	6.6(5.1 - 8.5)	0.2(0.2 - 0.3)	6.6(4.9 - 8.6)	0.1(-0.1 to 0.5)	0.17(-1.25 to 1.61)	0.82
San Marino	SMR	0(0 - 0)	19.6(12.3 - 30.1)	0(0 - 0)	18.5(9.2 - 31.7)	-0.1(-0.5 to 0.4)	-0.3(-0.58 to -0.03)	0.030
Netherlands	NLD	0.5(0.3 - 0.6)	7.4(5.3 - 9.9)	0.5(0.4 - 0.7)	9.1(6.4 - 12.6)	0.1(-0.2 to 0.5)	0.55(0.27 to 0.82)	<0.001
Denmark	DNK	0.1(0.1 - 0.2)	6.9(5 - 9.3)	0.1(0.1 - 0.1)	4(2.8 - 5.6)	-0.4(-0.6 to -0.2)	-1.67(-2.91 to -0.41)	0.009
Germany	DEU	5.9(4.4 - 7.9)	18.7(13.9 - 25.2)	4.7(3.2 - 6.6)	16.2(11 - 22.6)	-0.2(-0.4 to 0.1)	-0.44(-0.86 to -0.03)	0.036
Monaco	MCO	0(0 - 0)	13.5(7.8 - 21.9)	0(0 - 0)	23.4(12 - 40.1)	0.7(0 to 1.9)	1.8(1.67 to 1.92)	<0.001
Norway	NOR	0.2(0.1 - 0.2)	9.2(7.4 - 11.3)	0.2(0.1 - 0.2)	9(7 - 11.6)	0.1(-0.1 to 0.4)	-0.02(-0.64 to 0.61)	0.96
Switzerland	CHE	0.3(0.2 - 0.3)	9(6.6 - 12.1)	0.1(0.1 - 0.2)	4.5(3.2 - 6.2)	-0.4(-0.6 to -0.2)	-2.42(-4.2 to -0.61)	0.009
Bolivia (Plurinational State of)	BOL	0.2(0.1 - 0.3)	7.4(3.7 - 13)	0.5(0.3 - 1)	11.2(5.8 - 19.8)	2.2(0.8 to 5.3)	1.35(1.22 to 1.47)	<0.001
Ecuador	ECU	0.2(0.1 - 0.3)	5.4(3.8 - 7.6)	1.1(0.7 - 1.6)	14.7(9.6 - 21.7)	4.3(2.6 to 6.2)	3.66(3.11 to 4.21)	<0.001
Peru	PER	0.5(0.3 - 0.7)	6(3.7 - 9.5)	2(1.1 - 3.3)	13.1(7.2 - 21.9)	3.1(1.5 to 5.6)	2.63(1.31 to 3.96)	<0.001
Haiti	HTI	0.1(0 - 0.2)	4.3(1.9 - 8)	0.3(0.1 - 0.5)	4.9(2.3 - 9.1)	1.8(0.7 to 4)	0.84(0.06 to 1.63)	0.034
Belize	BLZ	0(0 - 0)	2.5(2 - 3.2)	0(0 - 0)	6.2(4.8 - 8)	5.9(4.5 to 7.5)	2.62(1.55 to 3.7)	<0.001
Dominican Republic	DOM	0.1(0.1 - 0.2)	4.4(2.8 - 7)	0.3(0.2 - 0.6)	7.5(4.3 - 12.7)	1.9(0.9 to 3.3)	1.72(1.31 to 2.13)	<0.001

Location	ISO Code	Numbers (thousands), 1990	Age-standardised rate (per 100 000), 1990	Numbers (thousands), 2021	Age-standardised rate (per 100 000), 2021	Total percentage change in number, 1990-2021	AAPC of age-standardised rate, 1990-2021	p value
Suriname	SUR	0(0 - 0)	5(2.9 - 8)	0(0 - 0)	7.2(4 - 12.6)	1.2(0.4 to 2.5)	1.11(0.03 to 2.19)	0.043
Saint Vincent and the Grenadines	VCT	0(0 - 0)	9.6(7.4 - 12.3)	0(0 - 0)	18.1(13.6 - 23.7)	1(0.6 to 1.6)	2.11(1.54 to 2.68)	<0.001
Guyana	GUY	0(0 - 0)	3.1(2.2 - 4.4)	0(0 - 0)	7.6(4.8 - 11.6)	1.3(0.5 to 2.3)	2.89(1.56 to 4.24)	<0.001
Cuba	CUB	0.4(0.3 - 0.5)	8.6(6.1 - 12)	0.5(0.3 - 0.7)	12.3(8.6 - 17.4)	0.2(-0.1 to 0.7)	1.14(0 to 2.29)	0.049
Grenada	GRD	0(0 - 0)	10.8(7.2 - 15.9)	0(0 - 0)	13.8(9.3 - 20.3)	0.6(0.1 to 1.3)	0.7(-0.14 to 1.55)	0.10
Saint Lucia	LCA	0(0 - 0)	9.1(7.1 - 11.5)	0(0 - 0)	14.3(10.5 - 19.4)	1.3(0.7 to 2.1)	1.41(0.34 to 2.49)	0.009
Jamaica	JAM	0(0 - 0.1)	4.5(3.1 - 6.5)	0.1(0.1 - 0.2)	10.8(6.4 - 17.4)	2.3(1.1 to 4.2)	3(1.8 to 4.22)	<0.001
Barbados	BRB	0(0 - 0)	8(6.3 - 10.1)	0(0 - 0)	12.2(8.6 - 17)	0.5(0.1 to 1)	1.47(0.66 to 2.28)	<0.001
Dominica	DMA	0(0 - 0)	3.7(2.3 - 5.6)	0(0 - 0)	6.5(3.9 - 10.5)	0.8(0.2 to 1.7)	1.84(1.24 to 2.44)	<0.001
Antigua and Barbuda	ATG	0(0 - 0)	8.5(6.5 - 11)	0(0 - 0)	10.7(8.2 - 13.9)	0.8(0.5 to 1.3)	0.47(-0.65 to 1.6)	0.41
Saint Kitts and Nevis	KNA	0(0 - 0)	5.7(4.5 - 7.1)	0(0 - 0)	4.8(3.1 - 6.9)	0.3(-0.1 to 0.8)	-0.55(-1.47 to 0.37)	0.24
Trinidad and Tobago	TTO	0(0 - 0)	5.9(4.7 - 7.3)	0.1(0 - 0.1)	12.4(8.5 - 17.4)	1.5(0.8 to 2.4)	2.33(1.14 to 3.52)	<0.001
Bahamas	BHS	0(0 - 0)	9(7.2 - 11.3)	0(0 - 0)	17.1(12.3 - 23.5)	1.7(0.9 to 2.7)	2.12(1.12 to 3.13)	<0.001
Bermuda	BMU	0(0 - 0)	10.8(7.7 - 15.3)	0(0 - 0)	20.5(13.9 - 29.2)	0.4(0 to 0.9)	2.03(0.84 to 3.24)	<0.001

Location	ISO Code	Numbers (thousands), 1990	Age-standardised rate (per 100 000), 1990	Numbers (thousands), 2021	Age-standardised rate (per 100 000), 2021	Total percentage change in number, 1990-2021	AAPC of age-standardised rate, 1990-2021	p value
United States Virgin Islands	VIR	0(0 - 0)	5(3 - 8.1)	0(0 - 0)	9.3(5.2 - 15.8)	0.1(-0.3 to 0.8)	2.46(2.2 to 2.72)	<0.001
Puerto Rico	PRI	0.1(0.1 - 0.2)	10.3(7.3 - 14.3)	0.2(0.1 - 0.2)	15.1(10.2 - 21.5)	0.1(-0.2 to 0.6)	1.19(-0.48 to 2.88)	0.16
Honduras	HND	0(0 - 0)	1.8(1 - 3.1)	0.1(0 - 0.2)	2.2(1 - 4.4)	2.3(0.7 to 5.1)	0.72(0.1 to 1.34)	0.022
Nicaragua	NIC	0(0 - 0.1)	3.7(2.2 - 5.7)	0.2(0.1 - 0.3)	6.1(3.5 - 10)	2.7(1.6 to 4.6)	1.39(0.85 to 1.93)	<0.001
Guatemala	GTM	0.1(0.1 - 0.1)	4.6(3.7 - 5.7)	0.5(0.4 - 0.6)	7.8(5.9 - 10.2)	3.1(2.2 to 4.1)	1.5(1.21 to 1.78)	<0.001
El Salvador	SLV	0.1(0.1 - 0.1)	4.9(3.7 - 6.6)	0.3(0.2 - 0.4)	12.6(8.5 - 18.1)	2.4(1.5 to 3.7)	3(1.98 to 4.02)	<0.001
Venezuela (Bolivarian Republic of)	VEN	0.3(0.2 - 0.3)	3.7(2.9 - 4.6)	1(0.7 - 1.4)	10(6.9 - 13.9)	2.6(1.5 to 3.9)	3.5(2.81 to 4.19)	<0.001
Colombia	COL	1.2(0.8 - 1.6)	9.2(6.5 - 12.5)	3(1.9 - 4.4)	14.7(9.4 - 21.9)	1.5(0.7 to 2.5)	1.63(1.28 to 1.99)	<0.001
Mexico	MEX	1.5(1.4 - 1.6)	4.8(4.6 - 5.1)	5.4(4.7 - 6.2)	10.5(9.1 - 11.9)	2.6(2.2 to 3.1)	2.58(2.27 to 2.89)	<0.001
Costa Rica	CRI	0.2(0.1 - 0.2)	12.9(9.1 - 18.2)	0.3(0.2 - 0.4)	13(8.7 - 18.3)	0.7(0.2 to 1.2)	-0.47(-1.73 to 0.81)	0.47
Panama	PAN	0.1(0.1 - 0.2)	14.3(9.8 - 20.8)	0.2(0.1 - 0.3)	11.6(7.7 - 17.1)	0.4(-0.1 to 1.1)	-0.75(-1.82 to 0.35)	0.18
Uruguay	URY	0.1(0.1 - 0.2)	10(6.8 - 14.8)	0.2(0.1 - 0.3)	15.5(10.6 - 22.1)	0.7(0.1 to 1.5)	1.92(1.54 to 2.3)	<0.001
Argentina	ARG	0.9(0.6 - 1.2)	7.4(5.3 - 10.4)	1.6(1.1 - 2.3)	8.9(6.2 - 12.7)	0.8(0.3 to 1.5)	0.63(-0.29 to 1.56)	0.18
Chile	CHL	0.5(0.3 - 0.6)	8.4(6 - 11.6)	1(0.7 - 1.4)	13.5(9.7 - 18.7)	1.2(0.5 to 2.4)	1.55(0.78 to 2.32)	<0.001
Paraguay	PRY	0.1(0.1 - 0.1)	5.9(3.5 - 9.3)	0.3(0.1 - 0.4)	8.8(4.9 - 14.5)	2.1(1 to 4.1)	1.34(0.64 to 2.04)	<0.001
Brazil	BRA	2.3(2 - 2.6)	3.9(3.5 - 4.4)	6.2(5.6 - 7)	6.9(6.2 - 7.7)	1.7(1.4 to 2)	1.74(1.52 to 1.97)	<0.001
Afghanistan	AFG	0.2(0 - 0.5)	7.4(1.7 - 22.6)	1.5(0.5 - 3.2)	14.9(5.2 - 31.7)	7.8(3.9 to 18.7)	2.31(2.09 to 2.54)	<0.001

Location	ISO Code	Numbers (thousands), 1990	Age-standardised rate (per 100 000), 1990	Numbers (thousands), 2021	Age-standardised rate (per 100 000), 2021	Total percentage change in number, 1990-2021	AAPC of age-standardised rate, 1990-2021	p value
Yemen	YEM	0.1(0 - 0.2)	2.5(1 - 5.6)	0.8(0.4 - 1.5)	6.3(3.2 - 12)	7(2.9 to 17)	3.06(1.99 to 4.14)	<0.001
Sudan	SDN	0.4(0.1 - 1)	5.2(1.5 - 14.8)	2.1(0.9 - 3.9)	12.3(5.2 - 23.4)	4.9(1.6 to 14.7)	2.81(2.58 to 3.04)	<0.001
Morocco	MAR	0.7(0.4 - 1.3)	7.6(4.1 - 13.8)	1.9(0.9 - 4.1)	12.9(6.3 - 27.4)	1.6(0.3 to 4.7)	1.7(1.54 to 1.86)	<0.001
Egypt	EGY	1.1(0.6 - 1.8)	5.5(3.3 - 8.9)	4.6(2.6 - 7.5)	11.1(6.3 - 18.1)	3.2(1.7 to 5.6)	2.22(2.14 to 2.31)	<0.001
Syrian Arab Republic	SYR	0.2(0.1 - 0.7)	6.1(2.4 - 16.2)	0.7(0.4 - 1.4)	16.7(7.9 - 32.5)	2(0.5 to 5.6)	3.37(2.84 to 3.92)	<0.001
Palestine	PSE	0.1(0 - 0.1)	11.2(5.8 - 20.3)	0.3(0.2 - 0.5)	16.9(9.8 - 27.8)	3.7(1.9 to 7)	1.31(0.69 to 1.94)	<0.001
Algeria	DZA	1.2(0.7 - 2.1)	13.6(7.6 - 23.8)	5.2(2.7 - 9.7)	28.1(14.4 - 52.8)	3.3(1.8 to 6)	2.42(2.2 to 2.64)	<0.001
Iraq	IRQ	0.9(0.5 - 1.5)	14(7.4 - 23.8)	4.2(2.3 - 7.4)	25.4(14.3 - 45.2)	3.7(1.9 to 7.3)	1.82(1.25 to 2.4)	<0.001
Tunisia	TUN	0.5(0.2 - 0.8)	14.6(7.8 - 25.9)	1.4(0.7 - 2.5)	28.6(14.7 - 53.2)	2(0.7 to 4.4)	2.18(1.87 to 2.5)	<0.001
Iran (Islamic Republic of)	IRN	1.1(0.6 - 1.7)	5.7(3.4 - 8.9)	8.2(3.8 - 11.6)	20.1(9.6 - 28.4)	6.6(3.3 to 10)	4.07(3.59 to 4.56)	<0.001
Turkey	TUR	3(1.5 - 5.2)	13.5(6.9 - 23.6)	6.4(3.6 - 10.9)	19.3(10.8 - 32.8)	1.2(0.3 to 2.5)	1.15(0.67 to 1.63)	<0.001
Jordan	JOR	0.2(0.1 - 0.4)	17.4(9.4 - 30.3)	1.2(0.7 - 2)	23.4(13.4 - 39.8)	4.4(2.5 to 8.2)	1.1(0.5 to 1.71)	<0.001
Libya	LBY	0.3(0.2 - 0.6)	23.4(12.2 - 44.3)	1.4(0.7 - 2.6)	43.7(20.7 - 82.7)	3.1(1.3 to 6.3)	2.14(1.26 to 3.03)	<0.001
Lebanon	LBN	0.2(0.1 - 0.4)	20.7(10.6 - 37.1)	0.7(0.4 - 1.2)	27.4(16.1 - 45.8)	2.2(0.9 to 4.7)	0.92(0.61 to 1.23)	<0.001
Bahrain	BHR	0(0 - 0.1)	11.8(6.1 - 20.9)	0.2(0.1 - 0.3)	21.9(12.4 - 36.6)	4.4(2.5 to 8.3)	1.98(1.09 to 2.88)	<0.001
Oman	OMN	0.1(0 - 0.1)	6(3.3 - 10.8)	0.4(0.2 - 0.7)	14(7.3 - 24.2)	6.5(3.3 to 11.2)	2.79(2.08 to 3.5)	<0.001
Saudi Arabia	SAU	0.8(0.4 - 1.5)	13.2(6.8 - 23.4)	10.9(5.8 - 19)	50(26.7 - 88.1)	12.2(6.5 to 24.7)	4.39(4.18 to 4.6)	<0.001
Kuwait	KWT	0.2(0.2 - 0.3)	25.8(18.1 - 35.8)	0.7(0.5 - 1)	25.3(17.7 - 36)	2(1.2 to 3)	-0.3(-6.01 to 5.75)	0.92

Location	ISO Code	Numbers (thousands), 1990	Age-standardised rate (per 100 000), 1990	Numbers (thousands), 2021	Age-standardised rate (per 100 000), 2021	Total percentage change in number, 1990-2021	AAPC of age-standardised rate, 1990-2021	p value
Qatar	QAT	0(0 - 0.1)	13.3(7.3 - 22.8)	0.5(0.3 - 0.8)	21.4(12.4 - 35.4)	11.8(6.8 to 18.8)	1.35(-0.29 to 3.01)	0.11
United Arab Emirates	ARE	0.1(0.1 - 0.2)	12.2(6.2 - 21.3)	0.9(0.5 - 1.6)	20.4(11.1 - 35)	6.3(3.7 to 10.9)	1.78(0.73 to 2.84)	<0.001
Nepal	NPL	0.4(0.2 - 0.8)	6.3(2.8 - 11.7)	1.9(0.9 - 3.7)	14.7(6.8 - 29)	3.3(1.5 to 7.6)	2.83(2.6 to 3.07)	<0.001
Bhutan	BTN	0(0 - 0)	6.1(2.9 - 11.2)	0(0 - 0.1)	13.6(6.1 - 31.1)	2.2(0.5 to 5.7)	2.54(2.27 to 2.81)	<0.001
Bangladesh	BGD	2.6(1.3 - 4.7)	6.6(3.3 - 11.8)	10.6(4.5 - 25.8)	15.6(6.7 - 37.6)	3.1(1.1 to 7.3)	2.89(2.56 to 3.21)	<0.001
Pakistan	PAK	4.1(2.5 - 6.7)	11(6.8 - 17.7)	23.9(13.6 - 40.3)	24.9(14.3 - 42)	4.8(2.6 to 8.7)	2.64(2.43 to 2.86)	<0.001
India	IND	21.1(16 - 29.9)	6.4(4.9 - 9.1)	87.6(67 - 119)	14.5(11.1 - 19.7)	3.2(2 to 4.9)	2.59(2.02 to 3.17)	<0.001
Democratic People's Republic of Korea	PRK	0.7(0.4 - 1.3)	9.2(4.5 - 16.9)	1.5(0.7 - 2.8)	14.3(7 - 26.5)	1.1(0.2 to 2.7)	1.44(1.32 to 1.56)	<0.001
China	CHN	27.9(20.9 - 35.7)	5.4(4.1 - 6.9)	68.6(53.6 - 90.9)	12.8(10 - 17)	1.5(0.8 to 2.7)	2.87(2.55 to 3.19)	<0.001
Taiwan (Province of China)	TWN	2.7(1.9 - 3.7)	28.8(20.3 - 39.7)	3.1(2.1 - 4.5)	36.4(24.4 - 52.5)	0.2(-0.1 to 0.6)	0.84(0.51 to 1.16)	<0.001
Papua New Guinea	PNG	0(0 - 0.1)	2.6(1.1 - 4.9)	0.1(0.1 - 0.3)	3.7(1.7 - 7.3)	3.1(1.5 to 5.7)	1.2(0.89 to 1.5)	<0.001
Solomon Islands	SLB	0(0 - 0)	2.2(1 - 4)	0(0 - 0)	5(2.7 - 9.1)	4.5(2.4 to 8.4)	2.68(2.38 to 2.97)	<0.001
Vanuatu	VUT	0(0 - 0)	2.6(1.4 - 4.7)	0(0 - 0)	4.7(2.6 - 7.9)	3(1.4 to 5.8)	1.98(1.58 to 2.38)	<0.001

Location	ISO Code	Numbers (thousands), 1990	Age-standardised rate (per 100 000), 1990	Numbers (thousands), 2021	Age-standardised rate (per 100 000), 2021	Total percentage change in number, 1990-2021	AAPC of age-standardised rate, 1990-2021	p value
Kiribati	KIR	0(0 - 0)	0.1(0.1 - 0.3)	0(0 - 0)	0.2(0.1 - 0.4)	1.6(0.5 to 3.6)	1.26(1.2 to 1.32)	<0.001
Marshall Islands	MHL	0(0 - 0)	3(1.8 - 4.8)	0(0 - 0)	6.3(3.3 - 11.4)	2.3(1 to 4.3)	2.41(2.26 to 2.55)	<0.001
Tuvalu	TUV	0(0 - 0)	3.3(1.8 - 5.6)	0(0 - 0)	6.6(3.9 - 10.9)	1.6(0.6 to 3.7)	2.24(2.13 to 2.34)	<0.001
Micronesia (Federated States of)	FSM	0(0 - 0)	3.7(2.1 - 6.1)	0(0 - 0)	7(3.9 - 12)	1.1(0.2 to 2.6)	2.1(2.04 to 2.15)	<0.001
Samoa	WSM	0(0 - 0)	12.3(6.1 - 22.1)	0(0 - 0)	24.2(10.7 - 48.4)	1.7(0.5 to 3.7)	2.24(2.06 to 2.42)	<0.001
Nauru	NRU	0(0 - 0)	5.3(2.7 - 9)	0(0 - 0)	9.7(4.4 - 18)	1.1(0.3 to 2.6)	2.01(1.82 to 2.19)	<0.001
Tonga	TON	0(0 - 0)	3.4(2 - 5.8)	0(0 - 0)	6.6(3.4 - 11.9)	1.4(0.4 to 3.2)	2.18(1.9 to 2.45)	<0.001
Fiji	FJI	0(0 - 0.1)	11.5(6.4 - 19.6)	0.1(0 - 0.1)	15(8.1 - 27.3)	0.6(-0.1 to 1.7)	0.9(0.29 to 1.52)	0.004
Tokelau	TKL	0(0 - 0)	4(2 - 6.9)	0(0 - 0)	12.1(6.9 - 20.9)	1.8(0.6 to 4)	3.86(3.2 to 4.53)	<0.001
American Samoa	ASM	0(0 - 0)	6.5(3.3 - 11.5)	0(0 - 0)	13.4(7.2 - 22.9)	0.9(0.1 to 2.7)	2.42(1.97 to 2.86)	<0.001
Niue	NIU	0(0 - 0)	4.6(2.6 - 8)	0(0 - 0)	12.3(6.9 - 21.1)	1(0.2 to 2.7)	3.27(2.92 to 3.62)	<0.001
Palau	PLW	0(0 - 0)	5.6(3 - 9.5)	0(0 - 0)	10.7(5.8 - 18.8)	0.9(0.1 to 2)	2.07(1.91 to 2.23)	<0.001
Northern Mariana Islands	MNP	0(0 - 0)	3.9(1.9 - 7.3)	0(0 - 0)	6.7(4.1 - 10.5)	0.2(-0.3 to 1.3)	1.58(0.61 to 2.57)	0.001
Cook Islands	COK	0(0 - 0)	7.5(4.1 - 13.3)	0(0 - 0)	11.9(6 - 21.7)	0.3(-0.3 to 1.5)	1.61(0.82 to 2.4)	<0.001
Guam	GUM	0(0 - 0)	3(1.9 - 6)	0(0 - 0)	9.4(6.6 - 12.7)	1.8(0.3 to 3.4)	4.16(2.4 to 5.96)	<0.001
Timor-Leste	TLS	0(0 - 0)	3.5(1.7 - 6.4)	0(0 - 0.1)	6.5(3.3 - 11.8)	2(0.7 to 4.4)	2.02(1.11 to 2.95)	<0.001

Location	ISO Code	Numbers (thousands), 1990	Age-standardised rate (per 100 000), 1990	Numbers (thousands), 2021	Age-standardised rate (per 100 000), 2021	Total percentage change in number, 1990-2021	AAPC of age-standardised rate, 1990-2021	p value
Cambodia	KHM	0.2(0.1 - 0.4)	6.3(3 - 11.7)	0.9(0.5 - 1.8)	12.9(6.7 - 24.2)	3.4(1.4 to 6.8)	2.37(2.17 to 2.56)	<0.001
Lao People's Democratic Republic	LAO	0.1(0 - 0.1)	5.2(2.1 - 10)	0.3(0.2 - 0.6)	9.9(4.8 - 18.9)	3.3(1.3 to 7)	2.07(1.88 to 2.25)	<0.001
Myanmar	MMR	1.2(0.5 - 2.3)	7.5(3.3 - 14.3)	2.3(1.2 - 4.1)	10.3(5.3 - 18.7)	0.9(0.1 to 2.6)	1.06(0.95 to 1.18)	<0.001
Viet Nam	VNM	3.4(1.6 - 6.5)	12.9(6.4 - 24.6)	16.3(7.2 - 30.6)	38.8(16.9 - 73.2)	3.8(2.1 to 6.9)	3.61(3.49 to 3.73)	<0.001
Maldives	MDV	0(0 - 0)	3.5(1.2 - 6.5)	0(0 - 0)	4.3(2.4 - 7.5)	4.9(2.1 to 14.5)	0.74(0.12 to 1.35)	0.019
Philippines	PHL	3.2(2.3 - 4.3)	13.8(9.8 - 18.6)	7.4(5.2 - 11.3)	16.5(11.6 - 25.1)	1.3(0.7 to 2.4)	0.55(0.24 to 0.85)	<0.001
Indonesia	IDN	4.6(2.8 - 6.3)	6.3(3.9 - 8.8)	10.9(7.1 - 16.3)	9.3(6 - 13.9)	1.4(0.6 to 2.9)	1.28(1.07 to 1.48)	<0.001
Thailand	THA	2.5(1.5 - 4.1)	10.3(6.1 - 16.7)	5.2(2.9 - 9)	22.8(12.6 - 39)	1.1(0.2 to 2.5)	2.58(1.78 to 3.4)	<0.001
Sri Lanka	LKA	0.6(0.4 - 1.1)	9.1(5.2 - 14.9)	1.4(0.7 - 2.6)	17.1(8.3 - 32.1)	1.1(0.2 to 2.7)	2.17(1.21 to 3.15)	<0.001
Mauritius	MUS	0(0 - 0)	5.6(4.4 - 7)	0(0 - 0)	6.2(4.8 - 7.8)	0.1(-0.1 to 0.3)	-0.13(-3.31 to 3.15)	0.94
Seychelles	SYC	0(0 - 0)	3.4(1.9 - 5.6)	0(0 - 0)	5(2.8 - 8.5)	1.2(0.4 to 2.4)	1.45(1.03 to 1.86)	<0.001
Malaysia	MYS	0.9(0.4 - 1.6)	12.1(6.3 - 22)	3.1(1.7 - 5.5)	21.7(11.7 - 38.3)	2.6(1.4 to 4.4)	1.77(1.53 to 2.02)	<0.001
Central African Republic	CAF	0(0 - 0)	1.4(0.7 - 2.4)	0(0 - 0.1)	1.4(0.7 - 2.8)	1.2(0.3 to 2.4)	0.08(-0.11 to 0.27)	0.39
Democratic Republic of the Congo	COD	0.2(0.1 - 0.3)	1.4(0.6 - 2.6)	0.6(0.2 - 1.1)	1.7(0.8 - 3.5)	2.2(0.9 to 4.3)	0.72(0.45 to 0.99)	<0.001
Angola	AGO	0(0 - 0.1)	1.3(0.7 - 2.5)	0.3(0.1 - 0.5)	2.3(1 - 4.5)	4.3(2.2 to 7.7)	1.79(1.26 to 2.32)	<0.001

Location	ISO Code	Numbers (thousands), 1990	Age-standardised rate (per 100 000), 1990	Numbers (thousands), 2021	Age-standardised rate (per 100 000), 2021	Total percentage change in number, 1990-2021	AAPC of age-standardised rate, 1990-2021	p value
Congo	COG	0(0 - 0)	1.8(0.9 - 3.3)	0.1(0 - 0.1)	3.3(1.5 - 6.6)	3.7(1.7 to 7.1)	2.07(1.27 to 2.88)	<0.001
Gabon	GAB	0(0 - 0)	2.2(1.1 - 3.9)	0(0 - 0)	3.6(1.6 - 7.2)	2.2(0.7 to 4.5)	1.49(1.22 to 1.77)	<0.001
Equatorial Guinea	GNQ	0(0 - 0)	1.4(0.7 - 2.6)	0(0 - 0)	3.7(1.6 - 7.2)	11(5.3 to 21.5)	3.24(2.49 to 4)	<0.001
Somalia	SOM	0.1(0.1 - 0.3)	5.6(2.7 - 10.5)	0.5(0.2 - 1)	6.8(3.3 - 12.9)	2.4(1.1 to 4.6)	0.7(0.56 to 0.83)	<0.001
South Sudan	SSD	0.1(0.1 - 0.2)	5.7(2.6 - 11.2)	0.4(0.2 - 0.7)	11.1(5.2 - 22.8)	2(0.7 to 4.7)	2.33(1.92 to 2.74)	<0.001
Burundi	BDI	0.2(0.1 - 0.3)	8.6(4.2 - 16)	0.5(0.2 - 0.9)	9.4(4.5 - 18.5)	1.8(0.5 to 5.1)	0.27(0.1 to 0.45)	0.002
Mozambique	MOZ	0.3(0.1 - 0.7)	6.8(3.1 - 15)	1.3(0.6 - 2.9)	11.7(5.4 - 26.5)	3.2(1.4 to 6)	1.76(1.42 to 2.1)	<0.001
Ethiopia	ETH	3.2(1.8 - 5.1)	18.7(11 - 30)	11.3(6.4 - 21.7)	26.5(15.2 - 50.8)	2.6(1 to 6.5)	1.11(0.98 to 1.24)	<0.001
Malawi	MWI	0.3(0.1 - 0.5)	7.6(3.7 - 14.2)	1(0.4 - 2.2)	13.6(6 - 29.2)	2.8(1 to 6.3)	1.9(1.47 to 2.33)	<0.001
Madagascar	MDG	0.3(0.2 - 0.6)	7.9(4.2 - 14)	1.2(0.6 - 2.4)	11.4(5.5 - 21.6)	2.8(1.2 to 5.3)	1.16(1.05 to 1.26)	<0.001
Eritrea	ERI	0.1(0 - 0.1)	5.9(3.1 - 10.4)	0.3(0.1 - 0.5)	10(4.7 - 19.9)	2.9(1.2 to 6.1)	1.69(1.45 to 1.93)	<0.001
Uganda	UGA	0.4(0.2 - 0.7)	6.9(3.7 - 12.2)	3.4(1.8 - 6.4)	22.2(11.6 - 41)	7.6(3.9 to 14.4)	3.74(3.07 to 4.41)	<0.001
Rwanda	RWA	0.3(0.1 - 0.5)	11.2(5.8 - 19.9)	0.7(0.3 - 1.5)	13.1(6.1 - 27.5)	1.4(0.3 to 4.7)	0.45(-0.32 to 1.21)	0.25
United Republic of Tanzania	TZA	0.8(0.4 - 1.4)	8.7(4.5 - 16.2)	2.8(1.4 - 5.6)	13.2(6.4 - 25.7)	2.7(1.1 to 5.6)	1.33(1.18 to 1.48)	<0.001
Comoros	COM	0(0 - 0)	8.6(3.5 - 18.4)	0(0 - 0.1)	13.3(6.1 - 28.4)	2(0.6 to 5.1)	1(-1.45 to 3.5)	0.43
Djibouti	DJI	0(0 - 0)	5.4(2.6 - 10.9)	0.1(0 - 0.1)	9.7(4.2 - 21.9)	5.3(2.2 to 10.5)	2.04(1.83 to 2.26)	<0.001
Zambia	ZMB	0.2(0.1 - 0.4)	9.4(4.9 - 16.3)	1.5(0.5 - 4)	19.9(7.3 - 52.6)	4.9(1.5 to 14.3)	2.5(2.27 to 2.73)	<0.001
Kenya	KEN	0.2(0.1 - 0.3)	2.6(1.6 - 4.2)	1(0.6 - 1.7)	4.9(3 - 8.6)	3.7(2 to 6.4)	2.03(1.57 to 2.5)	<0.001

Location	ISO Code	Numbers (thousands), 1990	Age-standardised rate (per 100 000), 1990	Numbers (thousands), 2021	Age-standardised rate (per 100 000), 2021	Total percentage change in number, 1990-2021	AAPC of age-standardised rate, 1990-2021	p value
Zimbabwe	ZWE	0.3(0.1 - 0.5)	7.8(4 - 13.9)	1(0.4 - 1.9)	16.9(7.6 - 33)	2.7(1.1 to 5.5)	2.55(1.77 to 3.35)	<0.001
Lesotho	LSO	0(0 - 0)	1.6(0.7 - 3.3)	0(0 - 0)	3.2(1.6 - 6)	2.2(0.7 to 4.7)	2.34(1.63 to 3.05)	<0.001
Eswatini	SWZ	0(0 - 0)	3(1.5 - 5.6)	0(0 - 0)	4.9(2.1 - 9.7)	2(0.6 to 4.5)	1.56(1.39 to 1.73)	<0.001
Namibia	NAM	0(0 - 0)	3.3(1.6 - 6.1)	0.1(0 - 0.1)	6.7(3.1 - 13)	3.1(1.3 to 6)	2.37(1.82 to 2.92)	<0.001
Botswana	BWA	0(0 - 0)	2.7(1.2 - 5.6)	0(0 - 0.1)	3.4(1.5 - 7)	2.1(0.8 to 4.2)	0.69(-0.23 to 1.62)	0.14
South Africa	ZAF	0.7(0.6 - 1.1)	5.3(4.2 - 7.6)	1.2(0.9 - 1.9)	4.8(3.7 - 7.5)	0.6(0.3 to 1.6)	-0.32(-1.33 to 0.71)	0.55
Niger	NER	0(0 - 0.1)	1.1(0.5 - 2.3)	0.1(0 - 0.2)	1.1(0.5 - 2.2)	1.8(0.4 to 3.8)	-0.14(-0.45 to 0.17)	0.38
Chad	TCD	0(0 - 0)	1.1(0.5 - 2.3)	0.1(0 - 0.1)	1.3(0.6 - 2.5)	2.4(0.7 to 5.2)	0.71(0.55 to 0.86)	<0.001
Mali	MLI	0.2(0.1 - 0.3)	6.7(3.5 - 12.1)	0.6(0.3 - 1.3)	8.3(3.8 - 16.8)	2.5(0.8 to 5)	0.66(0.41 to 0.91)	<0.001
Burkina Faso	BFA	0(0 - 0.1)	1.7(0.8 - 3.6)	0.1(0.1 - 0.3)	1.8(0.8 - 3.6)	1.8(0.5 to 4)	0.04(-0.3 to 0.38)	0.82
Guinea	GIN	0(0 - 0.1)	2.5(1.4 - 4.5)	0.2(0.1 - 0.4)	3.9(2 - 7.9)	2.8(1.2 to 5.7)	1.35(1.16 to 1.55)	<0.001
Liberia	LBR	0(0 - 0)	1.3(0.6 - 2.6)	0(0 - 0.1)	2.3(1 - 4.3)	3(1.1 to 6.2)	1.74(1.39 to 2.08)	<0.001
Guinea-Bissau	GNB	0(0 - 0)	2.1(1 - 4)	0(0 - 0)	2.2(1.1 - 4.2)	1.5(0.3 to 3.6)	0.28(0.06 to 0.5)	0.014
Sierra Leone	SLE	0(0 - 0)	1.2(0.5 - 2.5)	0.1(0 - 0.1)	1.7(0.8 - 3.7)	2.3(0.8 to 5.1)	1.33(0.9 to 1.76)	<0.001
Benin	BEN	0(0 - 0)	1.2(0.6 - 2.5)	0.1(0 - 0.1)	1.5(0.7 - 3.1)	2.5(1.1 to 5)	0.61(0.36 to 0.85)	<0.001
Senegal	SEN	0(0 - 0.1)	1.6(0.7 - 3.4)	0.1(0 - 0.2)	1.8(0.8 - 4)	1.6(0.4 to 3.8)	0.26(-0.43 to 0.96)	0.46
Togo	TGO	0(0 - 0)	1.8(0.8 - 3.7)	0.1(0 - 0.1)	2.1(0.9 - 4.2)	1.9(0.7 to 3.9)	0.42(0.14 to 0.7)	0.003
Gambia	GMB	0(0 - 0)	1.9(0.9 - 3.7)	0(0 - 0.1)	3.4(1.5 - 6.9)	3.7(1.6 to 7.3)	1.86(0.68 to 3.05)	0.002
Coted'Ivoire	CIV	0.2(0.1 - 0.3)	3.7(1.8 - 7.1)	0.7(0.3 - 1.4)	6.7(3 - 13.3)	3.5(1.5 to 7.1)	1.97(1.46 to 2.48)	<0.001
Cameroon	CMR	0.1(0 - 0.1)	2.1(1 - 4.2)	0.3(0.1 - 0.5)	2.4(1.1 - 4.6)	2.9(1.2 to 5.6)	0.38(0.14 to 0.62)	0.002

Location	ISO Code	Numbers (thousands), 1990	Age-standardised rate (per 100 000), 1990	Numbers (thousands), 2021	Age-standardised rate (per 100 000), 2021	Total percentage change in number, 1990-2021	AAPC of age-standardised rate, 1990-2021	p value
Mauritania	MRT	0(0 - 0)	1.3(0.6 - 2.4)	0(0 - 0.1)	2(0.9 - 4.1)	2.3(0.7 to 5.7)	1.42(1.09 to 1.75)	<0.001
Nigeria	NGA	0.1(0.1 - 0.2)	0.3(0.2 - 0.6)	0.5(0.2 - 0.9)	0.6(0.3 - 1.2)	3.7(1.5 to 7.6)	2(1.71 to 2.3)	<0.001
Sao Tome and Principe	STP	0(0 - 0)	0.7(0.1 - 1.6)	0(0 - 0)	1.6(0.4 - 4)	4.4(2.2 to 9.3)	2.43(1.36 to 3.51)	<0.001
Cabo Verde	CPV	0(0 - 0)	0.7(0.3 - 1.4)	0(0 - 0)	4(0.7 - 8.3)	15.4(0.8 to 46.1)	6.03(5.39 to 6.67)	<0.001
Ghana	GHA	0(0 - 0)	0.2(0.1 - 0.3)	0(0 - 0.1)	0.4(0.2 - 0.7)	3.7(1.9 to 6.8)	1.92(1.78 to 2.06)	<0.001

Data in parentheses are 95% uncertainty intervals for numbers, total percentage change, and 95% CIs for age-standardised rates, and AAPCs. AAPC=average annual percentage change.

Table S7: DALYs of thyroid cancer in adolescents and young adults (AYAs), and their average annual percentage changes in 204 countries/territories from 1990 to 2021.

Location	ISO Code	Numbers (thousands), 1990	Age-standardised rate (per 100 000), 1990	Numbers (thousands), 2021	Age-standardised rate (per 100 000), 2021	Total percentage change in number, 1990-2021	AAPC of age-standardised rate, 1990-2021	p value
Tajikistan	TJK	0(0 - 0)	0.1(0 - 0.1)	0(0 - 0)	0(0 - 0.1)	0.5(-0.2 to 1.8)	-1.35(-1.63 to -1.08)	<0.001
Kyrgyzstan	KGZ	0.2(0.1 - 0.3)	10.8(7.5 - 15)	0.1(0.1 - 0.2)	4.9(3.3 - 7)	-0.3(-0.6 to 0.1)	-2.69(-5.4 to 0.1)	0.06
Mongolia	MNG	0(0 - 0.1)	5.4(3.3 - 8.5)	0.1(0 - 0.1)	4.5(3 - 6.6)	0.5(-0.1 to 1.3)	-0.5(-1.38 to 0.38)	0.26
Uzbekistan	UZB	0.1(0.1 - 0.1)	1(0.7 - 1.3)	0.3(0.2 - 0.4)	1.8(1.2 - 2.5)	2.4(1 to 4.5)	2.01(1.44 to 2.58)	<0.001
Turkmenistan	TKM	0.1(0.1 - 0.1)	6.6(5.9 - 7.4)	0.1(0.1 - 0.1)	5(3.8 - 6.6)	0.1(-0.2 to 0.5)	-0.98(-3.3 to 1.39)	0.42
Azerbaijan	AZE	0.1(0.1 - 0.2)	3.4(1.9 - 5.5)	0.1(0.1 - 0.2)	2.5(1.3 - 4.2)	0.1(-0.5 to 1.4)	-1.13(-1.78 to -0.47)	<0.001
Armenia	ARM	0.1(0 - 0.1)	3.9(2.8 - 5.4)	0(0 - 0.1)	4(2.8 - 5.5)	-0.2(-0.5 to 0.4)	0.36(-0.95 to 1.69)	0.59
Kazakhstan	KAZ	0.8(0.7 - 0.9)	11.5(10.3 - 12.9)	0.4(0.3 - 0.5)	4.9(3.9 - 6.1)	-0.5(-0.6 to -0.4)	-2.93(-5.27 to -0.52)	0.017
Georgia	GEO	0.1(0.1 - 0.1)	4.7(3.4 - 6.5)	0.1(0.1 - 0.1)	6.8(4.7 - 9.5)	-0.2(-0.5 to 0.3)	0.7(-1.36 to 2.8)	0.51
Albania	ALB	0.1(0 - 0.1)	4.5(3.1 - 6.4)	0(0 - 0)	2.7(1.5 - 4.3)	-0.6(-0.8 to -0.3)	-1.84(-2.49 to -1.19)	<0.001
Bosnia and Herzegovina	BIH	0.1(0 - 0.1)	3.3(2.5 - 4.1)	0(0 - 0)	1.8(1.2 - 2.9)	-0.7(-0.8 to -0.5)	-1.94(-2.62 to -1.27)	<0.001
North Macedonia	MKD	0(0 - 0)	4.2(3.3 - 5.2)	0(0 - 0)	2.1(1.4 - 3.2)	-0.5(-0.6 to -0.2)	-2.25(-2.88 to -1.61)	<0.001
Bulgaria	BGR	0.2(0.1 - 0.2)	5.2(4.1 - 6.4)	0.1(0 - 0.1)	2.8(2.1 - 3.7)	-0.6(-0.7 to -0.5)	-1.98(-3.03 to -0.92)	<0.001
Romania	ROU	0.4(0.3 - 0.6)	5(3.8 - 6.5)	0.1(0.1 - 0.2)	2(1.4 - 2.8)	-0.7(-0.8 to -0.6)	-2.82(-3.56 to -2.08)	<0.001
Hungary	HUN	0.3(0.2 - 0.4)	7.2(5.6 - 9)	0.1(0 - 0.1)	2.1(1.5 - 2.9)	-0.8(-0.8 to -0.7)	-3.78(-4.9 to -2.65)	<0.001
Serbia	SRB	0.1(0.1 - 0.2)	3.6(2.4 - 5.6)	0.1(0 - 0.1)	2(1.2 - 3.2)	-0.5(-0.7 to -0.2)	-1.68(-1.93 to -1.43)	<0.001
Montenegro	MNE	0(0 - 0)	4.3(3.3 - 5.6)	0(0 - 0)	3.6(2.5 - 5.2)	-0.3(-0.5 to 0)	-0.23(-0.96 to 0.51)	0.54
Croatia	HRV	0.1(0.1 - 0.1)	5(3.9 - 6.5)	0(0 - 0)	1.9(1.3 - 2.6)	-0.7(-0.8 to -0.6)	-3.2(-4.59 to -1.79)	<0.001
Slovakia	SVK	0.1(0.1 - 0.2)	5.1(3.4 - 7.6)	0.1(0 - 0.1)	2.9(1.8 - 4.5)	-0.5(-0.7 to -0.2)	-1.79(-2.38 to -1.19)	<0.001

Location	ISO Code	Numbers (thousands), 1990	Age-standardised rate (per 100 000), 1990	Numbers (thousands), 2021	Age-standardised rate (per 100 000), 2021	Total percentage change in number, 1990-2021	AAPC of age-standardised rate, 1990-2021	p value
Poland	POL	1.2(1.2 - 1.3)	7.7(7.2 - 8.4)	0.4(0.3 - 0.5)	2.7(2.3 - 3.2)	-0.7(-0.7 to -0.6)	-3.11(-4.16 to -2.06)	<0.001
Czechia	CZE	0.2(0.2 - 0.3)	5.5(4.2 - 7.1)	0.1(0.1 - 0.1)	2.2(1.5 - 3.2)	-0.6(-0.8 to -0.5)	-2.94(-3.77 to -2.11)	<0.001
Slovenia	SVN	0(0 - 0.1)	4.9(3.9 - 6.2)	0(0 - 0)	1.3(0.9 - 1.8)	-0.8(-0.9 to -0.7)	-4.46(-5.23 to -3.69)	<0.001
Republic of Moldova	MDA	0(0 - 0.1)	2.7(2.3 - 3)	0(0 - 0)	2(1.7 - 2.5)	-0.4(-0.5 to -0.3)	-1.01(-1.44 to -0.57)	<0.001
Ukraine	UKR	0.7(0.5 - 0.9)	3.4(2.5 - 4.6)	0.6(0.4 - 0.9)	3.8(2.5 - 5.5)	-0.1(-0.4 to 0.4)	0.43(-0.48 to 1.35)	0.36
Belarus	BLR	0.2(0.2 - 0.3)	6(4.3 - 8.1)	0.1(0.1 - 0.1)	2.6(1.7 - 3.9)	-0.6(-0.8 to -0.5)	-3.19(-5.18 to -1.16)	0.002
Russian Federation	RUS	2.3(2.2 - 2.5)	3.7(3.5 - 4)	1.8(1.6 - 2.1)	3.3(2.9 - 3.8)	-0.2(-0.3 to -0.1)	-0.37(-2.12 to 1.42)	0.69
Latvia	LVA	0.1(0 - 0.1)	6.4(4.5 - 8.8)	0(0 - 0)	2.9(2 - 4.1)	-0.7(-0.8 to -0.5)	-2.77(-4.64 to -0.86)	0.005
Estonia	EST	0(0 - 0.1)	6.8(4.9 - 9.6)	0(0 - 0)	2.4(1.6 - 3.2)	-0.7(-0.8 to -0.7)	-3.75(-5.13 to -2.36)	<0.001
Lithuania	LTU	0.1(0.1 - 0.1)	6.1(4.5 - 8.4)	0(0 - 0)	2.7(1.9 - 3.7)	-0.7(-0.8 to -0.6)	-2.74(-3.8 to -1.66)	<0.001
Australia	AUS	0.2(0.2 - 0.3)	3.4(2.6 - 4.6)	0.3(0.2 - 0.4)	2.8(1.9 - 4.1)	0.1(-0.2 to 0.6)	-0.6(-1.89 to 0.7)	0.36
New Zealand	NZL	0(0 - 0)	2.9(2.3 - 3.6)	0(0 - 0.1)	2.6(1.9 - 3.4)	0.3(-0.1 to 0.7)	-0.82(-1.46 to -0.18)	0.012
Brunei Darussalam	BRN	0(0 - 0)	4.7(2.6 - 7.6)	0(0 - 0)	3.5(2.1 - 5.5)	0.4(-0.1 to 1.1)	-0.83(-1.11 to -0.55)	<0.001
Singapore	SGP	0.1(0 - 0.1)	3.4(2.6 - 4.5)	0(0 - 0)	1.5(1 - 2.1)	-0.4(-0.6 to -0.2)	-2.17(-3.8 to -0.5)	0.011
Japan	JPN	1(0.9 - 1.2)	2.3(2 - 2.7)	0.7(0.6 - 0.9)	2.1(1.8 - 2.6)	-0.3(-0.4 to -0.2)	-0.2(-0.65 to 0.25)	0.38
Republic of Korea	KOR	0.8(0.5 - 1.2)	3.9(2.5 - 6)	0.5(0.3 - 0.9)	3.1(1.7 - 5.1)	-0.3(-0.6 to 0)	-0.73(-1.07 to -0.4)	<0.001
Greenland	GRL	0(0 - 0)	5.5(2.5 - 9.4)	0(0 - 0)	1.8(1 - 3.1)	-0.7(-0.9 to -0.4)	-3.42(-3.99 to -2.84)	<0.001
United States of America	USA	2.8(2.5 - 3.1)	2.6(2.3 - 2.9)	3.8(3.3 - 4.4)	3.3(2.9 - 3.8)	0.4(0.3 to 0.5)	0.84(0.09 to 1.6)	0.028
Canada	CAN	0.4(0.3 - 0.6)	3.7(2.7 - 5)	0.3(0.2 - 0.5)	2.6(1.8 - 3.7)	-0.2(-0.4 to 0)	-0.98(-1.92 to -0.03)	0.044
Portugal	PRT	0.2(0.1 - 0.2)	4.7(3.5 - 6.2)	0.1(0 - 0.1)	2.1(1.4 - 3)	-0.6(-0.7 to -0.5)	-2.58(-3.6 to -1.54)	<0.001

Location	ISO Code	Numbers (thousands), 1990	Age-standardised rate (per 100 000), 1990	Numbers (thousands), 2021	Age-standardised rate (per 100 000), 2021	Total percentage change in number, 1990-2021	AAPC of age-standardised rate, 1990-2021	p value
Spain	ESP	0.6(0.5 - 0.8)	4.4(3.4 - 5.6)	0.2(0.2 - 0.3)	1.6(1.1 - 2.3)	-0.6(-0.7 to -0.5)	-3.3(-3.72 to -2.88)	<0.001
Greece	GRC	0.1(0.1 - 0.1)	2.8(2.4 - 3.2)	0.1(0.1 - 0.1)	2(1.6 - 2.5)	-0.4(-0.5 to -0.3)	-1.09(-1.5 to -0.68)	<0.001
Malta	MLT	0(0 - 0)	3.4(2.5 - 4.4)	0(0 - 0)	2(1.4 - 2.8)	-0.4(-0.6 to -0.1)	-1.1(-2.6 to 0.43)	0.16
Italy	ITA	1.1(1 - 1.3)	5.3(4.7 - 6)	0.5(0.4 - 0.6)	2.8(2.4 - 3.5)	-0.6(-0.6 to -0.5)	-2.04(-2.32 to -1.77)	<0.001
Israel	ISR	0.1(0.1 - 0.1)	4.1(3.1 - 5.2)	0.1(0.1 - 0.1)	2.3(1.7 - 3)	0(-0.3 to 0.3)	-1.42(-4.11 to 1.33)	0.31
Cyprus	CYP	0(0 - 0)	4.7(2.8 - 7.6)	0(0 - 0)	1.9(1.1 - 3.3)	-0.2(-0.5 to 0.5)	-2.88(-4.07 to -1.68)	<0.001
France	FRA	1.3(1 - 1.6)	5.5(4.3 - 7.1)	0.8(0.5 - 1.1)	3.6(2.4 - 5.2)	-0.4(-0.6 to -0.2)	-1.4(-1.79 to -1.01)	<0.001
Belgium	BEL	0.2(0.1 - 0.2)	4.1(3.2 - 5.2)	0.1(0.1 - 0.1)	2.5(1.8 - 3.2)	-0.4(-0.6 to -0.3)	-1.91(-2.72 to -1.09)	<0.001
Austria	AUT	0.2(0.1 - 0.2)	5.5(4.2 - 7)	0.1(0.1 - 0.1)	2.7(1.9 - 3.7)	-0.5(-0.6 to -0.3)	-2.32(-2.92 to -1.71)	<0.001
United Kingdom	GBR	0.6(0.6 - 0.7)	2.9(2.8 - 3.1)	0.5(0.5 - 0.6)	2.2(2 - 2.4)	-0.2(-0.2 to -0.1)	-1.01(-2.11 to 0.1)	0.07
Finland	FIN	0.1(0.1 - 0.1)	5.5(4.2 - 7)	0(0 - 0.1)	2(1.5 - 2.8)	-0.7(-0.7 to -0.5)	-3.04(-4.3 to -1.76)	<0.001
Andorra	AND	0(0 - 0)	3.2(1.8 - 4.9)	0(0 - 0)	2.4(1.3 - 4)	-0.2(-0.5 to 0.5)	-1.04(-1.28 to -0.8)	<0.001
Ireland	IRL	0(0 - 0.1)	3(2.3 - 3.8)	0(0 - 0)	1.8(1.3 - 2.5)	-0.2(-0.5 to 0.1)	-1.48(-2.62 to -0.32)	0.013
Iceland	ISL	0(0 - 0)	10.7(8.2 - 13.8)	0(0 - 0)	5.1(3.6 - 7.1)	-0.4(-0.6 to -0.2)	-2.32(-3.41 to -1.22)	<0.001
Luxembourg	LUX	0(0 - 0)	5.9(5.2 - 6.8)	0(0 - 0)	1.9(1.5 - 2.4)	-0.5(-0.6 to -0.4)	-3.6(-4.25 to -2.94)	<0.001
Sweden	SWE	0.1(0.1 - 0.1)	2.6(2.1 - 3.1)	0.1(0 - 0.1)	1.8(1.4 - 2.3)	-0.2(-0.4 to 0)	-0.92(-2.24 to 0.41)	0.17
San Marino	SMR	0(0 - 0)	5.5(3.5 - 8.2)	0(0 - 0)	3.6(1.7 - 6)	-0.4(-0.6 to 0)	-1.57(-1.74 to -1.41)	<0.001
Netherlands	NLD	0.2(0.1 - 0.2)	3(2.4 - 3.9)	0.1(0.1 - 0.2)	2.3(1.7 - 3.1)	-0.3(-0.5 to -0.2)	-0.98(-1.51 to -0.45)	<0.001
Denmark	DNK	0.1(0 - 0.1)	2.7(2.1 - 3.4)	0(0 - 0)	0.9(0.6 - 1.3)	-0.7(-0.8 to -0.6)	-3.3(-4.55 to -2.03)	<0.001
Germany	DEU	1.5(1.2 - 1.9)	4.8(3.8 - 6.1)	0.7(0.5 - 1)	2.5(1.7 - 3.6)	-0.5(-0.6 to -0.4)	-2.21(-2.73 to -1.7)	<0.001
Monaco	MCO	0(0 - 0)	4(2.3 - 6.1)	0(0 - 0)	4.4(2.3 - 7.6)	0.1(-0.4 to 0.8)	0.37(0.27 to 0.46)	<0.001

Location	ISO Code	Numbers (thousands), 1990	Age-standardised rate (per 100 000), 1990	Numbers (thousands), 2021	Age-standardised rate (per 100 000), 2021	Total percentage change in number, 1990-2021	AAPC of age-standardised rate, 1990-2021	p value
Norway	NOR	0.1(0.1 - 0.1)	3.5(3.1 - 3.9)	0(0 - 0)	1.9(1.6 - 2.3)	-0.4(-0.5 to -0.3)	-1.92(-2.49 to -1.35)	<0.001
Switzerland	CHE	0.1(0.1 - 0.2)	4.7(3.8 - 6)	0(0 - 0)	1.2(0.9 - 1.5)	-0.7(-0.8 to -0.7)	-4.75(-6.45 to -3.03)	<0.001
Bolivia (Plurinational State of)	BOL	0.2(0.1 - 0.4)	9.9(5.4 - 16)	0.4(0.2 - 0.6)	7.4(4.1 - 12.4)	0.6(0 to 1.9)	-0.92(-1.04 to -0.8)	<0.001
Ecuador	ECU	0.2(0.1 - 0.2)	5(3.9 - 6.4)	0.5(0.4 - 0.7)	6.9(4.9 - 9.4)	1.6(1 to 2.4)	1.26(0.75 to 1.77)	<0.001
Peru	PER	0.5(0.3 - 0.7)	5.6(3.7 - 8.1)	0.7(0.4 - 1.1)	4.7(2.8 - 7.2)	0.5(0 to 1.3)	-0.55(-1.52 to 0.43)	0.27
Haiti	HTI	0.2(0.1 - 0.3)	7.2(3.4 - 12.8)	0.3(0.1 - 0.6)	5.7(2.8 - 10.2)	0.8(0.2 to 2.1)	-0.62(-1.07 to -0.15)	0.009
Belize	BLZ	0(0 - 0)	1.9(1.7 - 2.1)	0(0 - 0)	3.4(2.8 - 3.9)	3.8(3.1 to 4.6)	1.58(0.07 to 3.11)	0.041
Dominican Republic	DOM	0.1(0.1 - 0.2)	4.1(2.9 - 6)	0.2(0.1 - 0.3)	4.3(2.6 - 7)	0.7(0.2 to 1.5)	-0.03(-0.55 to 0.5)	0.92
Suriname	SUR	0(0 - 0)	4.4(2.9 - 6.7)	0(0 - 0)	4.4(2.6 - 6.9)	0.5(0 to 1.1)	-0.13(-1.04 to 0.79)	0.78
Saint Vincent and the Grenadines	VCT	0(0 - 0)	7.1(6.2 - 8.1)	0(0 - 0)	9.4(7.8 - 11.4)	0.4(0.1 to 0.6)	0.94(0.14 to 1.75)	0.021
Guyana	GUY	0(0 - 0)	3.8(2.9 - 5)	0(0 - 0)	6(4.1 - 8.7)	0.5(0 to 1.1)	1.47(0.12 to 2.84)	0.033
Cuba	CUB	0.2(0.1 - 0.2)	4(3.1 - 5)	0.1(0.1 - 0.2)	3.7(2.8 - 5)	-0.2(-0.4 to 0)	-0.46(-1.38 to 0.47)	0.33
Grenada	GRD	0(0 - 0)	8.5(6.3 - 11.5)	0(0 - 0)	6.8(5 - 9.3)	0(-0.3 to 0.3)	-0.87(-1.55 to -0.19)	0.012
Saint Lucia	LCA	0(0 - 0)	6.4(5.6 - 7.3)	0(0 - 0)	6.2(4.9 - 7.7)	0.4(0.1 to 0.7)	-0.2(-1 to 0.61)	0.63
Jamaica	JAM	0(0 - 0)	2.4(1.8 - 3.3)	0(0 - 0.1)	4.2(2.6 - 6.4)	1.3(0.6 to 2.5)	2.19(0.49 to 3.91)	0.011
Barbados	BRB	0(0 - 0)	4.6(4 - 5.2)	0(0 - 0)	4.4(3.3 - 5.8)	-0.1(-0.3 to 0.2)	-0.21(-1.08 to 0.68)	0.65
Dominica	DMA	0(0 - 0)	3(2 - 4.4)	0(0 - 0)	4.2(2.6 - 6.4)	0.3(-0.1 to 0.9)	1.01(0.36 to 1.67)	0.002
Antigua and Barbuda	ATG	0(0 - 0)	4.5(3.8 - 5.2)	0(0 - 0)	3.8(3.2 - 4.5)	0.2(0 to 0.4)	-0.53(-1.6 to 0.55)	0.34
Saint Kitts and Nevis	KNA	0(0 - 0)	5.4(4.5 - 6.4)	0(0 - 0)	2.5(1.7 - 3.6)	-0.3(-0.5 to 0)	-2.43(-3.12 to -1.74)	<0.001

Location	ISO Code	Numbers (thousands), 1990	Age-standardised rate (per 100 000), 1990	Numbers (thousands), 2021	Age-standardised rate (per 100 000), 2021	Total percentage change in number, 1990-2021	AAPC of age-standardised rate, 1990-2021	p value
Trinidad and Tobago	TTO	0(0 - 0)	4.4(3.9 - 4.9)	0(0 - 0)	5.8(4.3 - 7.8)	0.5(0.1 to 1)	1.02(-0.22 to 2.27)	0.11
Bahamas	BHS	0(0 - 0)	6(5.2 - 6.9)	0(0 - 0)	8(6.1 - 10.5)	0.9(0.4 to 1.5)	0.86(-0.07 to 1.8)	0.07
Bermuda	BMU	0(0 - 0)	4.9(3.8 - 6.2)	0(0 - 0)	4.6(3.4 - 6.3)	-0.3(-0.5 to -0.1)	-0.27(-1.82 to 1.3)	0.73
United States Virgin Islands	VIR	0(0 - 0)	3(2 - 4.5)	0(0 - 0)	4.7(2.6 - 7.9)	-0.1(-0.4 to 0.4)	2.22(1.77 to 2.67)	<0.001
Puerto Rico	PRI	0.1(0.1 - 0.1)	5(3.8 - 6.4)	0(0 - 0.1)	4.3(3.1 - 5.9)	-0.3(-0.5 to -0.1)	-0.56(-1.72 to 0.61)	0.34
Honduras	HND	0(0 - 0)	2(1.3 - 3.1)	0.1(0 - 0.1)	1.5(0.7 - 2.7)	0.9(0 to 2.4)	-1.02(-1.33 to -0.7)	<0.001
Nicaragua	NIC	0(0 - 0.1)	3(2 - 4.4)	0.1(0 - 0.1)	2.7(1.7 - 4.2)	1(0.5 to 1.7)	-0.5(-0.89 to -0.1)	0.014
Guatemala	GTM	0.2(0.1 - 0.2)	5.7(5.2 - 6.4)	0.3(0.2 - 0.4)	4.7(3.9 - 5.7)	1(0.7 to 1.3)	-0.36(-1.37 to 0.67)	0.50
El Salvador	SLV	0.1(0.1 - 0.1)	4.8(3.8 - 5.9)	0.1(0.1 - 0.2)	5.2(3.7 - 7)	0.4(0.1 to 0.9)	0.14(-0.75 to 1.04)	0.76
Venezuela (Bolivarian Republic of)	VEN	0.2(0.2 - 0.2)	2.8(2.5 - 3.1)	0.4(0.3 - 0.6)	4.7(3.4 - 6.2)	1.1(0.5 to 1.8)	1.88(1.27 to 2.5)	<0.001
Colombia	COL	1(0.8 - 1.2)	7.3(5.6 - 9.3)	1(0.7 - 1.5)	5.1(3.5 - 7.2)	0(-0.2 to 0.4)	-1.19(-1.67 to -0.7)	<0.001
Mexico	MEX	1.3(1.2 - 1.4)	4.1(3.9 - 4.3)	2.5(2.2 - 2.8)	4.8(4.3 - 5.4)	0.9(0.7 to 1.1)	0.54(0.27 to 0.81)	<0.001
Costa Rica	CRI	0.1(0.1 - 0.1)	5.9(4.5 - 7.6)	0.1(0.1 - 0.1)	4(3 - 5.4)	0.1(-0.1 to 0.4)	-1.78(-3.03 to -0.51)	0.006
Panama	PAN	0.1(0.1 - 0.1)	8.6(6.5 - 11.5)	0.1(0 - 0.1)	4.1(2.9 - 5.6)	-0.2(-0.4 to 0.1)	-2.49(-3.64 to -1.33)	<0.001
Uruguay	URY	0.1(0 - 0.1)	5.5(3.9 - 7.4)	0.1(0 - 0.1)	5.3(3.8 - 7.2)	0.1(-0.2 to 0.5)	0.14(-0.62 to 0.9)	0.72
Argentina	ARG	0.6(0.5 - 0.7)	4.8(3.8 - 6.1)	0.6(0.5 - 0.8)	3.5(2.6 - 4.7)	0.1(-0.2 to 0.4)	-0.99(-1.79 to -0.19)	0.016
Chile	CHL	0.3(0.2 - 0.4)	5.1(4 - 6.6)	0.3(0.2 - 0.4)	3.7(2.7 - 4.9)	0(-0.3 to 0.3)	-0.98(-1.75 to -0.21)	0.013
Paraguay	PRY	0.1(0 - 0.1)	4.3(2.7 - 6.7)	0.1(0.1 - 0.2)	4.2(2.5 - 6.5)	1(0.4 to 2)	-0.14(-0.6 to 0.33)	0.56
Brazil	BRA	2(1.8 - 2.2)	3.3(3 - 3.6)	3(2.7 - 3.3)	3.3(3 - 3.7)	0.5(0.4 to 0.6)	-0.1(-0.43 to 0.23)	0.54

Location	ISO Code	Numbers (thousands), 1990	Age-standardised rate (per 100 000), 1990	Numbers (thousands), 2021	Age-standardised rate (per 100 000), 2021	Total percentage change in number, 1990-2021	AAPC of age-standardised rate, 1990-2021	p value
Afghanistan	AFG	0.1(0 - 0.4)	5.8(1.4 - 17.5)	0.8(0.3 - 1.6)	7.4(2.9 - 14.6)	4.5(2.1 to 10.4)	0.79(0.41 to 1.16)	<0.001
Yemen	YEM	0.1(0 - 0.1)	1.5(0.7 - 3)	0.3(0.2 - 0.6)	2.4(1.3 - 4.3)	4.2(1.5 to 9.5)	1.51(0.84 to 2.19)	<0.001
Sudan	SDN	0.2(0.1 - 0.6)	3.1(1 - 8.4)	0.7(0.3 - 1.2)	4(1.9 - 7.1)	2.2(0.4 to 7.1)	0.84(0.67 to 1.02)	<0.001
Morocco	MAR	0.4(0.2 - 0.7)	3.9(2.2 - 6.7)	0.5(0.3 - 1)	3.5(1.8 - 7)	0.4(-0.3 to 1.8)	-0.35(-0.56 to -0.13)	0.001
Egypt	EGY	0.6(0.4 - 0.9)	2.9(1.8 - 4.3)	1.2(0.7 - 1.9)	2.9(1.8 - 4.6)	1.1(0.4 to 2.1)	0.01(-0.59 to 0.63)	0.96
Syrian Arab Republic	SYR	0.1(0 - 0.2)	2.4(1 - 5.9)	0.1(0.1 - 0.3)	3.2(1.6 - 5.9)	0.5(-0.2 to 2)	0.98(0.62 to 1.35)	<0.001
Palestine	PSE	0(0 - 0)	4(2.3 - 6.7)	0.1(0 - 0.1)	3.6(2.1 - 5.6)	1.7(0.7 to 3.3)	-0.39(-0.76 to -0.03)	0.035
Algeria	DZA	0.5(0.3 - 0.8)	5.4(3.2 - 8.8)	1.1(0.6 - 1.8)	5.8(3.1 - 10.3)	1.1(0.5 to 2.3)	0.31(0.18 to 0.43)	<0.001
Iraq	IRQ	0.4(0.2 - 0.6)	5.9(3.4 - 9.3)	0.9(0.5 - 1.6)	5.6(3.3 - 9.4)	1.4(0.6 to 3.1)	-0.24(-0.64 to 0.16)	0.23
Tunisia	TUN	0.1(0.1 - 0.2)	4.5(2.6 - 7.8)	0.2(0.1 - 0.4)	5.1(2.7 - 9)	0.7(0 to 1.6)	0.35(0.2 to 0.5)	<0.001
Iran (Islamic Republic of)	IRN	0.3(0.2 - 0.4)	1.5(1 - 2.1)	1.3(0.6 - 1.8)	3.3(1.6 - 4.5)	3.4(1.3 to 5.2)	2.52(2.15 to 2.89)	<0.001
Turkey	TUR	1.3(0.7 - 2.1)	5.8(3.2 - 9.3)	1.2(0.7 - 1.9)	3.6(2.1 - 5.9)	-0.1(-0.4 to 0.4)	-1.56(-1.98 to -1.14)	<0.001
Jordan	JOR	0.1(0 - 0.1)	5.8(3.4 - 9.5)	0.2(0.1 - 0.4)	4.4(2.5 - 7.5)	1.9(1 to 3.6)	-0.79(-1.39 to -0.19)	0.010
Libya	LBY	0.1(0.1 - 0.2)	7.5(4.3 - 13.5)	0.3(0.1 - 0.5)	9.1(4.7 - 16.4)	1.5(0.5 to 3.2)	0.58(-0.02 to 1.18)	0.06
Lebanon	LBN	0.1(0 - 0.1)	6.3(3.5 - 10.8)	0.1(0.1 - 0.2)	4.5(2.7 - 7.5)	0.7(0 to 1.9)	-1.05(-1.28 to -0.82)	<0.001
Bahrain	BHR	0(0 - 0)	3.9(2.1 - 6.4)	0(0 - 0)	3.7(2.1 - 6)	1.8(0.9 to 3.6)	-0.17(-0.89 to 0.55)	0.63
Oman	OMN	0(0 - 0)	2.2(1.3 - 3.8)	0.1(0 - 0.1)	2.6(1.3 - 4.5)	2.8(1.3 to 5.1)	0.61(0.02 to 1.2)	0.042
Saudi Arabia	SAU	0.4(0.2 - 0.6)	5.9(3.3 - 9.8)	2.1(1.2 - 3.5)	9.7(5.4 - 16.5)	4.5(2.2 to 8.9)	1.58(1.43 to 1.74)	<0.001
Kuwait	KWT	0(0 - 0.1)	5.5(4.1 - 7.5)	0.1(0.1 - 0.1)	3.6(2.4 - 5.1)	0.9(0.4 to 1.5)	-1.69(-7.32 to 4.28)	0.57
Qatar	QAT	0(0 - 0)	3.9(2.2 - 6.3)	0.1(0 - 0.1)	3.5(2 - 5.7)	6(3.4 to 9.6)	-0.54(-1.34 to 0.27)	0.19

Location	ISO Code	Numbers (thousands), 1990	Age-standardised rate (per 100 000), 1990	Numbers (thousands), 2021	Age-standardised rate (per 100 000), 2021	Total percentage change in number, 1990-2021	AAPC of age-standardised rate, 1990-2021	p value
United Arab Emirates	ARE	0.1(0 - 0.1)	5.2(2.8 - 8.4)	0.2(0.1 - 0.3)	4.8(2.7 - 8)	3.1(1.6 to 5.3)	0.16(-0.87 to 1.2)	0.76
Nepal	NPL	0.6(0.3 - 1.1)	8.8(4.3 - 15.4)	1.3(0.6 - 2.4)	9.8(4.9 - 18.5)	1(0.2 to 2.9)	0.35(0.26 to 0.45)	<0.001
Bhutan	BTN	0(0 - 0)	8.5(4.5 - 14.3)	0(0 - 0.1)	8.6(4 - 18.3)	0.4(-0.3 to 1.9)	0.05(-0.1 to 0.2)	0.53
Bangladesh	BGD	3.7(2 - 6.1)	9.2(5 - 15)	6.1(2.8 - 13.6)	9(4 - 19.7)	0.7(-0.1 to 2.2)	0.08(-0.28 to 0.45)	0.65
Pakistan	PAK	5.5(3.6 - 8.4)	14.2(9.4 - 21.6)	19.9(12.3 - 31.5)	20.5(12.7 - 32.3)	2.7(1.4 to 4.9)	1.23(1.13 to 1.34)	<0.001
India	IND	26.3(20.3 - 37.1)	7.9(6.1 - 11.2)	54(41.8 - 72)	8.9(6.9 - 11.9)	1.1(0.5 to 1.9)	0.3(-0.2 to 0.79)	0.24
Democratic People's Republic of Korea	PRK	0.4(0.2 - 0.7)	5.2(2.8 - 8.9)	0.5(0.3 - 0.9)	4.8(2.6 - 8.2)	0.2(-0.3 to 1)	-0.27(-0.33 to -0.21)	<0.001
China	CHN	20.3(15.7 - 25.9)	3.9(3 - 4.9)	17.2(13.4 - 22.4)	3.3(2.6 - 4.3)	-0.2(-0.3 to 0.2)	-0.53(-0.97 to -0.08)	0.021
Taiwan (Province of China)	TWN	0.8(0.6 - 1)	8.3(6.3 - 10.6)	0.5(0.3 - 0.7)	5.5(3.9 - 7.8)	-0.4(-0.5 to -0.2)	-1.43(-2.35 to -0.5)	0.003
Papua New Guinea	PNG	0(0 - 0.1)	2.4(1.1 - 4.4)	0.1(0.1 - 0.2)	2.8(1.3 - 5.1)	2.2(1.1 to 4.2)	0.54(0.27 to 0.81)	<0.001
Solomon Islands	SLB	0(0 - 0)	2.5(1.3 - 4.3)	0(0 - 0)	3.9(2.3 - 6.4)	2.7(1.4 to 5.1)	1.38(1.11 to 1.65)	<0.001
Vanuatu	VUT	0(0 - 0)	2.5(1.4 - 4.1)	0(0 - 0)	3.6(2.1 - 5.9)	2.2(1 to 4)	1.21(0.9 to 1.53)	<0.001
Kiribati	KIR	0(0 - 0)	0.2(0.1 - 0.4)	0(0 - 0)	0.2(0.1 - 0.4)	1(0.1 to 2.6)	0.35(0.24 to 0.47)	<0.001
Marshall Islands	MHL	0(0 - 0)	3(1.9 - 4.7)	0(0 - 0)	4.5(2.6 - 7.5)	1.3(0.5 to 2.4)	1.29(1.23 to 1.36)	<0.001
Tuvalu	TUV	0(0 - 0)	3.3(2 - 5.3)	0(0 - 0)	4(2.4 - 6.3)	0.6(0 to 1.6)	0.62(0.55 to 0.68)	<0.001
Micronesia (Federated States of)	FSM	0(0 - 0)	3.9(2.3 - 6.2)	0(0 - 0)	4.6(2.6 - 7.7)	0.3(-0.2 to 1.2)	0.54(0.5 to 0.58)	<0.001
Samoa	WSM	0(0 - 0)	8.1(4.4 - 13.9)	0(0 - 0)	9.8(4.8 - 17.9)	0.6(-0.1 to 1.6)	0.61(0.57 to 0.66)	<0.001
Nauru	NRU	0(0 - 0)	4.6(2.6 - 7.6)	0(0 - 0)	5.8(3 - 10.2)	0.4(-0.1 to 1.3)	0.75(0.71 to 0.79)	<0.001

Location	ISO Code	Numbers (thousands), 1990	Age-standardised rate (per 100 000), 1990	Numbers (thousands), 2021	Age-standardised rate (per 100 000), 2021	Total percentage change in number, 1990-2021	AAPC of age-standardised rate, 1990-2021	p value
Tonga	TON	0(0 - 0)	2.2(1.3 - 3.5)	0(0 - 0)	3.2(1.7 - 5.7)	0.8(0.1 to 2)	1.24(1.05 to 1.42)	<0.001
Fiji	FJI	0(0 - 0)	8.3(4.9 - 13)	0(0 - 0)	7.6(4.5 - 12.5)	0.1(-0.3 to 0.7)	-0.3(-0.57 to -0.03)	0.032
Tokelau	TKL	0(0 - 0)	3.1(1.7 - 5.3)	0(0 - 0)	5.1(3.1 - 8.4)	0.5(-0.1 to 1.4)	1.76(1.31 to 2.21)	<0.001
American Samoa	ASM	0(0 - 0)	3.3(1.8 - 5.4)	0(0 - 0)	5.1(3 - 8)	0.4(-0.1 to 1.5)	1.52(0.99 to 2.05)	<0.001
Niue	NIU	0(0 - 0)	3.1(1.8 - 5.2)	0(0 - 0)	5(3 - 8.3)	0.2(-0.3 to 1.1)	1.57(1.36 to 1.79)	<0.001
Palau	PLW	0(0 - 0)	3.5(2 - 5.8)	0(0 - 0)	5.1(2.8 - 8.9)	0.4(-0.1 to 1.4)	1.16(1.02 to 1.3)	<0.001
Northern Mariana Islands	MNP	0(0 - 0)	1.7(0.8 - 3.3)	0(0 - 0)	2.3(1.4 - 3.5)	0(-0.4 to 0.8)	0.67(-0.62 to 1.97)	0.31
Cook Islands	COK	0(0 - 0)	5.1(2.8 - 8.6)	0(0 - 0)	4.1(2.1 - 7.3)	-0.3(-0.7 to 0.3)	-0.69(-1.37 to -0.01)	0.048
Guam	GUM	0(0 - 0)	1.4(0.9 - 2.7)	0(0 - 0)	3.4(2.3 - 4.6)	1.2(-0.1 to 2.4)	3.14(1.55 to 4.76)	<0.001
Timor-Leste	TLS	0(0 - 0)	3.7(1.9 - 6.5)	0(0 - 0)	3.7(2.1 - 6.3)	0.7(0 to 1.8)	-0.1(-1 to 0.8)	0.82
Cambodia	KHM	0.2(0.1 - 0.4)	6.6(3.4 - 11.4)	0.5(0.3 - 0.8)	6.2(3.5 - 10.7)	1(0.2 to 2.3)	-0.16(-0.25 to -0.06)	0.001
Lao People's Democratic Republic	LAO	0.1(0 - 0.2)	6.5(3 - 11.7)	0.2(0.1 - 0.3)	5.7(3.1 - 10)	1(0.2 to 2.5)	-0.43(-0.6 to -0.27)	<0.001
Myanmar	MMR	1.2(0.6 - 2.2)	7.5(3.7 - 13.7)	1.1(0.6 - 1.8)	4.9(2.9 - 8.3)	-0.1(-0.5 to 0.6)	-1.36(-1.46 to -1.27)	<0.001
Viet Nam	VNM	2.1(1.1 - 3.7)	7.7(4.1 - 13.9)	4.3(2 - 7.6)	10.4(4.9 - 18.4)	1.1(0.4 to 2.2)	0.96(0.87 to 1.05)	<0.001
Maldives	MDV	0(0 - 0)	2.6(1 - 4.5)	0(0 - 0)	1.4(0.8 - 2.5)	1.3(0.3 to 4)	-2(-2.69 to -1.3)	<0.001
Philippines	PHL	2.1(1.6 - 2.5)	8.7(6.9 - 10.6)	3.3(2.6 - 4.6)	7.3(5.8 - 10.2)	0.6(0.2 to 1.2)	-0.61(-0.89 to -0.33)	<0.001
Indonesia	IDN	3.7(2.4 - 5)	5.1(3.3 - 6.8)	5(3.4 - 7.1)	4.3(2.9 - 6.1)	0.3(-0.1 to 1)	-0.51(-0.58 to -0.45)	<0.001
Thailand	THA	1.3(0.9 - 2)	5.3(3.5 - 8)	1.2(0.7 - 2)	5.5(3.3 - 8.8)	-0.1(-0.4 to 0.5)	0.1(-0.7 to 0.91)	0.81
Sri Lanka	LKA	0.4(0.2 - 0.6)	5.2(3.4 - 7.9)	0.3(0.2 - 0.6)	4.2(2.3 - 7.2)	-0.1(-0.4 to 0.5)	-0.72(-1.61 to 0.17)	0.11

Location	ISO Code	Numbers (thousands), 1990	Age-standardised rate (per 100 000), 1990	Numbers (thousands), 2021	Age-standardised rate (per 100 000), 2021	Total percentage change in number, 1990-2021	AAPC of age-standardised rate, 1990-2021	p value
Mauritius	MUS	0(0 - 0)	2.6(2.2 - 2.9)	0(0 - 0)	1.8(1.5 - 2.1)	-0.3(-0.4 to -0.2)	-1.31(-3.92 to 1.36)	0.33
Seychelles	SYC	0(0 - 0)	1.8(1.1 - 2.8)	0(0 - 0)	1.5(0.9 - 2.4)	0.3(-0.1 to 0.8)	-0.3(-0.69 to 0.09)	0.14
Malaysia	MYS	0.5(0.3 - 0.9)	7.1(4 - 12)	0.9(0.5 - 1.6)	6.6(3.8 - 11)	0.8(0.3 to 1.5)	-0.45(-0.7 to -0.2)	<0.001
Central African Republic	CAF	0(0 - 0)	2.6(1.4 - 4.4)	0(0 - 0.1)	2.3(1.2 - 4.1)	0.8(0.2 to 1.7)	-0.49(-0.66 to -0.32)	<0.001
Democratic Republic of the Congo	COD	0.3(0.1 - 0.5)	2.1(1.1 - 3.6)	0.6(0.3 - 1.1)	1.8(0.8 - 3.3)	1.2(0.4 to 2.6)	-0.51(-0.69 to -0.33)	<0.001
Angola	AGO	0.1(0 - 0.1)	2.3(1.2 - 3.9)	0.3(0.1 - 0.5)	2.3(1.1 - 4.2)	2(0.9 to 3.9)	-0.04(-0.49 to 0.41)	0.86
Congo	COG	0(0 - 0)	2.7(1.5 - 4.6)	0.1(0 - 0.1)	2.8(1.4 - 5.1)	1.6(0.6 to 3.2)	0.29(-0.3 to 0.89)	0.33
Gabon	GAB	0(0 - 0)	2.8(1.6 - 4.6)	0(0 - 0)	2.6(1.3 - 4.8)	0.8(0 to 2)	-0.3(-0.6 to 0)	0.052
Equatorial Guinea	GNQ	0(0 - 0)	2.4(1.3 - 4.3)	0(0 - 0)	2.5(1.1 - 4.5)	3.7(1.6 to 7.8)	0.13(-0.45 to 0.72)	0.66
Somalia	SOM	0.3(0.1 - 0.5)	9.8(5.2 - 16.6)	0.8(0.4 - 1.4)	10.2(5.5 - 18)	1.9(0.9 to 3.6)	0.14(0.06 to 0.22)	<0.001
South Sudan	SSD	0.2(0.1 - 0.3)	8.1(4.1 - 14.8)	0.4(0.2 - 0.7)	11.3(5.9 - 21.1)	1.2(0.2 to 2.8)	1.21(0.77 to 1.65)	<0.001
Burundi	BDI	0.3(0.2 - 0.5)	14.3(7.8 - 24.7)	0.6(0.3 - 1.1)	11.1(5.7 - 21.2)	1(0.1 to 2.9)	-0.83(-1.22 to -0.44)	<0.001
Mozambique	MOZ	0.5(0.2 - 1)	10.3(5.2 - 21.8)	1.5(0.8 - 3.1)	13.6(6.9 - 28.2)	2.3(1 to 4.3)	0.89(0.72 to 1.07)	<0.001
Ethiopia	ETH	6(3.8 - 9.2)	34.4(22 - 52.9)	10.2(6.3 - 18.3)	23.2(14.3 - 41.6)	0.7(0 to 2.5)	-1.27(-1.36 to -1.17)	<0.001
Malawi	MWI	0.4(0.2 - 0.7)	10.8(5.9 - 19.7)	0.9(0.5 - 2)	12.3(6 - 25.4)	1.5(0.4 to 3.3)	0.48(0.31 to 0.64)	<0.001
Madagascar	MDG	0.5(0.3 - 0.8)	11(6.2 - 17.9)	1.3(0.7 - 2.2)	11.3(6 - 19.8)	1.7(0.6 to 3.2)	0.09(-0.01 to 0.18)	0.06
Eritrea	ERI	0.1(0.1 - 0.2)	10(5.8 - 16.4)	0.3(0.2 - 0.6)	11.6(5.9 - 21.8)	1.6(0.6 to 3.5)	0.41(0.19 to 0.62)	<0.001
Uganda	UGA	0.6(0.3 - 1)	9.7(5.5 - 16)	3.2(1.8 - 5.8)	20.3(11.5 - 35.9)	4.6(2.3 to 8.7)	2.3(1.88 to 2.72)	<0.001
Rwanda	RWA	0.5(0.3 - 0.8)	19.2(10.6 - 31.7)	0.6(0.3 - 1.2)	11.7(6 - 22.7)	0.3(-0.3 to 1.6)	-1.6(-1.78 to -1.41)	<0.001

Location	ISO Code	Numbers (thousands), 1990	Age-standardised rate (per 100 000), 1990	Numbers (thousands), 2021	Age-standardised rate (per 100 000), 2021	Total percentage change in number, 1990-2021	AAPC of age-standardised rate, 1990-2021	p value
United Republic of Tanzania	TZA	1(0.5 - 1.8)	11.1(6.2 - 19.5)	2.5(1.3 - 4.9)	11.6(5.8 - 22.1)	1.6(0.5 to 3.3)	0.15(0.02 to 0.28)	0.020
Comoros	COM	0(0 - 0)	11.6(5 - 23.5)	0(0 - 0.1)	12.3(6.1 - 24.3)	1(0.1 to 3.2)	-0.44(-3.12 to 2.31)	0.75
Djibouti	DJI	0(0 - 0)	7.2(3.6 - 13.8)	0(0 - 0.1)	8.8(4 - 18)	3.1(1.2 to 6.1)	0.52(0.1 to 0.95)	0.016
Zambia	ZMB	0.4(0.2 - 0.6)	13.7(7.8 - 22.6)	1.5(0.5 - 3.7)	19.2(7.2 - 47.3)	2.9(0.6 to 8.3)	1.1(0.9 to 1.31)	<0.001
Kenya	KEN	0.2(0.1 - 0.3)	2.7(1.7 - 4.2)	0.8(0.5 - 1.3)	3.7(2.4 - 6.3)	2.4(1.3 to 4.1)	1.01(0.85 to 1.17)	<0.001
Zimbabwe	ZWE	0.3(0.1 - 0.5)	7.6(4.2 - 12.9)	1(0.5 - 1.8)	16.3(7.7 - 30.7)	2.6(1 to 5)	2.46(1.42 to 3.5)	<0.001
Lesotho	LSO	0(0 - 0)	2.1(1 - 3.8)	0(0 - 0.1)	3.8(2 - 6.7)	1.9(0.6 to 3.8)	2.04(1.68 to 2.41)	<0.001
Eswatini	SWZ	0(0 - 0)	3.6(1.9 - 6)	0(0 - 0)	4.7(2.2 - 8.3)	1.4(0.3 to 3.2)	0.88(0.67 to 1.09)	<0.001
Namibia	NAM	0(0 - 0)	3.9(2 - 6.7)	0(0 - 0.1)	4.8(2.3 - 8.9)	1.4(0.4 to 3.1)	0.71(0.27 to 1.15)	0.002
Botswana	BWA	0(0 - 0)	3.3(1.6 - 6.2)	0(0 - 0.1)	2.8(1.2 - 5.3)	1(0.2 to 2.2)	-0.63(-1.3 to 0.03)	0.06
South Africa	ZAF	0.6(0.5 - 0.9)	4.4(3.6 - 6.1)	0.8(0.6 - 1.2)	3.1(2.4 - 4.6)	0.2(0 to 0.7)	-1.1(-1.83 to -0.35)	0.004
Niger	NER	0(0 - 0.1)	1.6(0.8 - 3.2)	0.1(0 - 0.2)	1(0.5 - 2.1)	0.9(0.1 to 2.3)	-1.46(-1.78 to -1.13)	<0.001
Chad	TCD	0(0 - 0.1)	1.5(0.8 - 2.9)	0.1(0 - 0.1)	1.4(0.7 - 2.4)	1.6(0.4 to 3.5)	-0.19(-0.47 to 0.09)	0.18
Mali	MLI	0.2(0.1 - 0.4)	8.9(5 - 15.3)	0.6(0.3 - 1.1)	7.3(3.6 - 14.2)	1.4(0.3 to 2.8)	-0.62(-0.89 to -0.34)	<0.001
Burkina Faso	BFA	0.1(0 - 0.1)	2.1(1 - 4.2)	0.1(0.1 - 0.2)	1.5(0.7 - 3)	1(0.2 to 2.4)	-1(-1.29 to -0.7)	<0.001
Guinea	GIN	0.1(0 - 0.1)	3.8(2.2 - 6.4)	0.2(0.1 - 0.4)	4.2(2.3 - 7.7)	1.7(0.6 to 3.6)	0.29(0.09 to 0.5)	0.006
Liberia	LBR	0(0 - 0)	1.9(0.9 - 3.5)	0(0 - 0.1)	1.6(0.8 - 3)	1.1(0.1 to 2.9)	-0.37(-0.67 to -0.08)	0.014
Guinea-Bissau	GNB	0(0 - 0)	3.2(1.7 - 5.8)	0(0 - 0)	2.3(1.1 - 4.1)	0.6(-0.1 to 1.8)	-1.12(-1.3 to -0.94)	<0.001
Sierra Leone	SLE	0(0 - 0)	1.5(0.7 - 2.9)	0(0 - 0.1)	1.4(0.7 - 2.8)	1.2(0.3 to 2.7)	-0.08(-0.43 to 0.26)	0.64
Benin	BEN	0(0 - 0)	1.6(0.8 - 3.1)	0.1(0 - 0.1)	1.2(0.6 - 2.4)	1.2(0.3 to 2.6)	-0.93(-1.2 to -0.66)	<0.001

Location	ISO Code	Numbers (thousands), 1990	Age-standardised rate (per 100 000), 1990	Numbers (thousands), 2021	Age-standardised rate (per 100 000), 2021	Total percentage change in number, 1990-2021	AAPC of age-standardised rate, 1990-2021	p value
Senegal	SEN	0(0 - 0.1)	1.9(0.9 - 3.8)	0.1(0 - 0.2)	1.3(0.7 - 2.7)	0.6(-0.1 to 2)	-1.24(-1.55 to -0.92)	<0.001
Togo	TGO	0(0 - 0)	2(1.1 - 4)	0(0 - 0.1)	1.5(0.7 - 2.9)	0.9(0.1 to 2.1)	-0.96(-1.13 to -0.79)	<0.001
Gambia	GMB	0(0 - 0)	2.1(1.1 - 3.8)	0(0 - 0)	2.6(1.3 - 4.8)	2.3(0.9 to 4.4)	0.68(-0.97 to 2.36)	0.42
Coted'Ivoire	CIV	0.2(0.1 - 0.4)	4.3(2.3 - 7.9)	0.5(0.2 - 1)	4.8(2.3 - 9.5)	1.7(0.6 to 3.8)	0.36(-0.06 to 0.78)	0.09
Cameroon	CMR	0.1(0 - 0.2)	2.4(1.3 - 4.5)	0.2(0.1 - 0.4)	1.8(0.9 - 3.2)	1.5(0.5 to 3.2)	-0.99(-1.09 to -0.88)	<0.001
Mauritania	MRT	0(0 - 0)	1.6(0.9 - 2.8)	0(0 - 0)	1.1(0.6 - 2.2)	0.5(-0.2 to 1.9)	-1.11(-1.56 to -0.66)	<0.001
Nigeria	NGA	0.1(0.1 - 0.2)	0.4(0.2 - 0.6)	0.3(0.2 - 0.6)	0.4(0.2 - 0.7)	1.8(0.6 to 3.8)	0.26(0.08 to 0.43)	0.003
Sao Tome and Principe	STP	0(0 - 0)	1.1(0.2 - 2.6)	0(0 - 0)	1.5(0.3 - 3.8)	2.1(0.9 to 4.9)	0.93(0.6 to 1.26)	<0.001
Cabo Verde	CPV	0(0 - 0)	0.5(0.2 - 1)	0(0 - 0)	2(0.3 - 4.4)	10.3(0 to 34.7)	4.91(4.01 to 5.81)	<0.001
Ghana	GHA	0(0 - 0)	0.3(0.2 - 0.4)	0(0 - 0.1)	0.3(0.2 - 0.5)	2(1 to 3.7)	0.51(0.33 to 0.69)	<0.001

Data in parentheses are 95% uncertainty intervals for numbers, total percentage change, and 95% CIs for age-standardised rates, and AAPCs. DALY=disability-adjusted life-year. AAPC=average annual percentage change.

Table S8. The results of frontier analysis based on SDI and age-standardized DALYs rate for thyroid cancer in adolescents and young adults from 1990 to 2021. SDI= Sociodemographic index. ASDR=Age-standardized DALYs rates. DALYs=disability-adjusted life-years.

Location	SDI	Rate of ASDR	Frontier ASDR	Effective difference	Effective difference rank (ASDR rank)
Somalia	0.077688109	10.21(5.5 to 18.01)	9.36	0.85	9 (189)
Niger	0.168072774	1.03(0.5 to 2.08)	1.03	0	2 (6)
Chad	0.240436019	1.37(0.71 to 2.38)	1.03	0.34	5 (12)
Mali	0.268579941	7.28(3.57 to 14.2)	1.02	6.26	171 (174)
South Sudan	0.278371125	11.26(5.87 to 21.12)	1.03	10.24	190 (192)
Burkina Faso	0.285118402	1.54(0.74 to 2.96)	0.86	0.69	7 (19)
Burundi	0.289374365	11.12(5.71 to 21.18)	0.5	10.63	192 (191)
Central African Republic	0.30916769	2.25(1.16 to 4.05)	0.42	1.83	34 (49)
Mozambique	0.326462614	13.58(6.86 to 28.15)	0.38	13.2	199 (199)
Guinea	0.336401293	4.18(2.28 to 7.74)	0.37	3.81	114 (121)
Afghanistan	0.337199998	7.43(2.95 to 14.62)	0.38	7.05	176 (176)
Liberia	0.352442452	1.64(0.82 to 3.03)	0.37	1.27	17 (22)

**Location**	**SDI**	**Rate of ASDR**	**Frontier ASDR**	**Effective difference**	**Effective difference rank (ASDR rank)**
Guinea-Bissau	0.353109621	2.28(1.13 to 4.07)	0.38	1.9	36 (51)
Sierra Leone	0.358665881	1.42(0.67 to 2.79)	0.38	1.04	11 (14)
Ethiopia	0.358823295	23.25(14.33 to 41.56)	0.38	22.87	204 (204)
Benin	0.373486574	1.2(0.6 to 2.38)	0.37	0.84	8 (9)
Democratic Republic of the Congo	0.383179849	1.79(0.85 to 3.32)	0.27	1.52	23 (26)
Malawi	0.384553634	12.32(6.02 to 25.36)	0.29	12.03	197 (198)
Madagascar	0.400246943	11.27(6.01 to 19.8)	0.27	11	193 (193)
Eritrea	0.403863943	11.63(5.92 to 21.83)	0.27	11.36	195 (195)
Senegal	0.408054193	1.35(0.67 to 2.73)	0.27	1.08	12 (11)
Togo	0.408533695	1.53(0.74 to 2.95)	0.27	1.27	16 (17)
Gambia	0.40971416	2.63(1.27 to 4.82)	0.27	2.36	58 (66)
Papua New Guinea	0.417797443	2.76(1.29 to 5.1)	0.22	2.54	65 (73)
Uganda	0.423261181	20.31(11.48 to 35.86)	0.23	20.08	202 (202)
Coted'Ivoire	0.425941883	4.82(2.26 to 9.47)	0.23	4.59	137 (142)

Location	SDI	Rate of ASDR	Frontier ASDR	Effective difference	Effective difference rank (ASDR rank)
Solomon Islands	0.429360316	3.87(2.26 to 6.36)	0.23	3.65	107 (112)
Nepal	0.433174635	9.81(4.9 to 18.47)	0.23	9.58	187 (188)
Rwanda	0.435588706	11.67(5.97 to 22.68)	0.23	11.43	196 (196)
Timor-Leste	0.444667619	3.66(2.08 to 6.27)	0.24	3.42	94 (104)
United Republic of Tanzania	0.446568273	11.58(5.83 to 22.07)	0.23	11.35	194 (194)
Haiti	0.448278285	5.66(2.78 to 10.16)	0.24	5.41	160 (162)
Yemen	0.450376375	2.42(1.29 to 4.31)	0.22	2.19	52 (56)
Angola	0.453721949	2.25(1.08 to 4.24)	0.22	2.03	46 (48)
Bhutan	0.473062378	8.57(3.97 to 18.31)	0.05	8.52	181 (180)
Vanuatu	0.473100706	3.64(2.13 to 5.85)	0.05	3.59	105 (103)
Cambodia	0.473621491	6.21(3.52 to 10.73)	0.05	6.17	170 (169)
Zimbabwe	0.473819486	16.35(7.73 to 30.74)	0.05	16.3	200 (200)
Comoros	0.475978688	12.3(6.05 to 24.35)	0.04	12.26	198 (197)
Cameroon	0.479691223	1.79(0.88 to 3.23)	0.04	1.75	27 (25)

Location	SDI	Rate of ASDR	Frontier ASDR	Effective difference	Effective difference rank (ASDR rank)
Djibouti	0.487958371	8.77(4.04 to 18.03)	0.04	8.73	182 (181)
Lao People's Democratic Republic	0.489136091	5.69(3.06 to 9.98)	0.04	5.65	164 (163)
Bangladesh	0.492420885	8.96(4.04 to 19.74)	0.05	8.91	184 (183)
Mauritania	0.4989451	1.13(0.57 to 2.2)	0.04	1.1	13 (7)
Nigeria	0.503390833	0.41(0.23 to 0.72)	0.04	0.37	6 (4)
Pakistan	0.504028689	20.49(12.68 to 32.31)	0.04	20.45	203 (203)
Sao Tome and Principe	0.505413747	1.55(0.3 to 3.85)	0.04	1.51	22 (20)
Zambia	0.505948954	19.2(7.15 to 47.27)	0.04	19.16	201 (201)
Lesotho	0.510393066	3.84(2.03 to 6.73)	0.04	3.8	113 (111)
Honduras	0.513037248	1.5(0.72 to 2.74)	0.04	1.46	20 (16)
Kenya	0.523768077	3.72(2.42 to 6.3)	0.04	3.68	109 (107)
Nicaragua	0.523958472	2.75(1.68 to 4.16)	0.04	2.71	74 (72)
Kiribati	0.527186583	0.25(0.13 to 0.43)	0.04	0.21	3 (2)
Cabo Verde	0.533534539	2.01(0.3 to 4.38)	0.04	1.97	39 (36)

Location	SDI	Rate of ASDR	Frontier ASDR	Effective difference	Effective difference rank (ASDR rank)
Myanmar	0.53390084	4.95(2.87 to 8.25)	0.04	4.91	149 (148)
Guatemala	0.539972424	4.75(3.95 to 5.66)	0.04	4.71	142 (140)
Tajikistan	0.541511187	0.04(0.02 to 0.06)	0.04	0	1 (1)
Sudan	0.541949735	4.01(1.87 to 7.13)	0.04	3.97	118 (116)
Morocco	0.562698301	3.5(1.75 to 7.05)	0.04	3.46	96 (94)
El Salvador	0.563775188	5.18(3.74 to 6.99)	0.04	5.14	158 (157)
Ghana	0.56493039	0.31(0.18 to 0.53)	0.04	0.27	4 (3)
Democratic People's Republic of Korea	0.569854634	4.78(2.63 to 8.17)	0.04	4.74	143 (141)
Marshall Islands	0.574091128	4.51(2.6 to 7.5)	0.04	4.47	132 (131)
India	0.575401649	8.92(6.91 to 11.87)	0.04	8.88	183 (182)
Tuvalu	0.576620529	3.98(2.44 to 6.33)	0.04	3.95	115 (113)
Congo	0.583075236	2.77(1.35 to 5.06)	0.04	2.73	75 (74)
Eswatini	0.585459713	4.73(2.22 to 8.31)	0.04	4.69	141 (139)
Micronesia (Federated States of)	0.587534967	4.59(2.63 to 7.68)	0.04	4.55	136 (135)

Location	SDI	Rate of ASDR	Frontier ASDR	Effective difference	Effective difference rank (ASDR rank)
Samoa	0.593392769	9.8(4.78 to 17.91)	0.04	9.76	189 (187)
Venezuela (Bolivarian Republic of)	0.596513059	4.65(3.37 to 6.17)	0.04	4.61	138 (136)
Bolivia (Plurinational State of)	0.599010799	7.43(4.09 to 12.38)	0.04	7.4	178 (177)
Kyrgyzstan	0.603979328	4.88(3.32 to 6.96)	0.04	4.84	147 (146)
Egypt	0.606787094	2.92(1.77 to 4.55)	0.04	2.88	82 (81)
Belize	0.610229002	3.36(2.84 to 3.94)	0.04	3.32	93 (92)
Namibia	0.617564872	4.83(2.29 to 8.9)	0.04	4.8	144 (143)
Mongolia	0.617621565	4.54(3.04 to 6.65)	0.04	4.5	133 (132)
Dominican Republic	0.619388201	4.34(2.63 to 7.03)	0.04	4.3	126 (125)
Syrian Arab Republic	0.623004075	3.19(1.6 to 5.92)	0.04	3.15	85 (84)
Nauru	0.625177834	5.8(2.96 to 10.16)	0.04	5.76	165 (164)
Tonga	0.626349936	3.19(1.74 to 5.66)	0.04	3.15	86 (85)
Viet Nam	0.627933721	10.4(4.88 to 18.44)	0.04	10.36	191 (190)
Palestine	0.631011665	3.56(2.14 to 5.62)	0.04	3.52	101 (99)

Location	SDI	Rate of ASDR	Frontier ASDR	Effective difference	Effective difference rank (ASDR rank)
Suriname	0.633665739	4.36(2.61 to 6.87)	0.04	4.32	128 (127)
Gabon	0.634691393	2.57(1.3 to 4.75)	0.04	2.53	64 (62)
Paraguay	0.635718099	4.21(2.52 to 6.48)	0.04	4.17	124 (123)
Saint Vincent and the Grenadines	0.637195963	9.38(7.77 to 11.39)	0.04	9.35	186 (185)
Botswana	0.642721629	2.8(1.24 to 5.32)	0.04	2.76	76 (75)
Guyana	0.650812335	6.04(4.12 to 8.66)	0.04	6.01	168 (167)
Maldives	0.650886627	1.38(0.8 to 2.49)	0.04	1.34	18 (13)
Philippines	0.651219329	7.29(5.79 to 10.18)	0.04	7.25	177 (175)
Brazil	0.653043887	3.32(3.05 to 3.67)	0.04	3.28	90 (89)
Colombia	0.655442913	5.06(3.5 to 7.19)	0.04	5.02	152 (151)
Indonesia	0.656868336	4.34(2.94 to 6.12)	0.04	4.3	127 (126)
Equatorial Guinea	0.657857456	2.51(1.14 to 4.54)	0.04	2.47	63 (61)
Algeria	0.659500924	5.81(3.08 to 10.26)	0.04	5.77	166 (165)
Ecuador	0.661017053	6.95(4.88 to 9.36)	0.04	6.91	175 (173)

Location	SDI	Rate of ASDR	Frontier ASDR	Effective difference	Effective difference rank (ASDR rank)
Peru	0.662054037	4.7(2.79 to 7.17)	0.04	4.66	140 (138)
Uzbekistan	0.662621694	1.77(1.23 to 2.51)	0.04	1.73	25 (23)
Iraq	0.662626231	5.57(3.3 to 9.4)	0.04	5.53	163 (161)
Mexico	0.664575304	4.84(4.29 to 5.42)	0.04	4.81	146 (145)
Cuba	0.668729864	3.73(2.77 to 5.02)	0.04	3.69	110 (108)
Grenada	0.668993028	6.77(4.97 to 9.33)	0.04	6.73	173 (171)
Saint Lucia	0.672509735	6.2(4.93 to 7.75)	0.04	6.16	169 (168)
Fiji	0.675051631	7.57(4.54 to 12.55)	0.04	7.53	179 (178)
South Africa	0.679626598	3.1(2.43 to 4.61)	0.04	3.06	84 (83)
Turkmenistan	0.682160776	4.98(3.79 to 6.58)	0.04	4.95	150 (149)
Tunisia	0.682432216	5.06(2.7 to 8.99)	0.04	5.02	153 (152)
Thailand	0.682547933	5.49(3.3 to 8.81)	0.04	5.45	161 (159)
Jamaica	0.683263064	4.16(2.65 to 6.4)	0.04	4.12	121 (119)
Tokelau	0.686425621	5.12(3.07 to 8.43)	0.04	5.08	156 (155)

Location	SDI	Rate of ASDR	Frontier ASDR	Effective difference	Effective difference rank (ASDR rank)
Azerbaijan	0.694851274	2.46(1.29 to 4.17)	0.04	2.42	60 (58)
Iran (Islamic Republic of)	0.697207398	3.31(1.63 to 4.54)	0.04	3.27	89 (88)
Costa Rica	0.700340477	3.99(2.95 to 5.35)	0.04	3.95	116 (114)
Sri Lanka	0.701534935	4.19(2.26 to 7.2)	0.04	4.15	123 (122)
Armenia	0.701833194	4(2.84 to 5.48)	0.04	3.96	117 (115)
Albania	0.706849791	2.71(1.55 to 4.35)	0.04	2.67	72 (70)
Panama	0.708864828	4.06(2.88 to 5.58)	0.04	4.02	119 (117)
Turkey	0.712692673	3.57(2.13 to 5.89)	0.04	3.54	102 (100)
Mauritius	0.718260446	1.81(1.51 to 2.15)	0.04	1.77	30 (29)
Uruguay	0.719283445	5.34(3.81 to 7.21)	0.04	5.3	159 (158)
China	0.72162976	3.33(2.6 to 4.34)	0.04	3.29	91 (90)
Bosnia and Herzegovina	0.723077893	1.8(1.18 to 2.91)	0.04	1.76	29 (28)
Argentina	0.723122973	3.53(2.63 to 4.66)	0.04	3.49	98 (96)
American Samoa	0.723727533	5.1(2.97 to 8.01)	0.04	5.06	154 (153)

Location	SDI	Rate of ASDR	Frontier ASDR	Effective difference	Effective difference rank (ASDR rank)
Kazakhstan	0.725144495	4.9(3.91 to 6.08)	0.04	4.86	148 (147)
Jordan	0.725307227	4.41(2.52 to 7.53)	0.04	4.37	130 (129)
Libya	0.725771399	9.11(4.67 to 16.36)	0.04	9.07	185 (184)
Niue	0.72622205	5(3.02 to 8.28)	0.04	4.96	151 (150)
Seychelles	0.730150775	1.54(0.94 to 2.36)	0.04	1.5	21 (18)
Republic of Moldova	0.732214875	2.02(1.66 to 2.46)	0.04	1.98	42 (39)
Georgia	0.732473604	6.85(4.7 to 9.48)	0.04	6.81	174 (172)
Malaysia	0.742523828	6.57(3.78 to 10.96)	0.04	6.53	172 (170)
Portugal	0.744151851	2.07(1.41 to 2.96)	0.04	2.03	47 (43)
Lebanon	0.744746351	4.55(2.72 to 7.45)	0.04	4.51	134 (133)
Barbados	0.746748764	4.41(3.33 to 5.78)	0.04	4.37	129 (128)
Dominica	0.746967185	4.18(2.62 to 6.43)	0.04	4.14	122 (120)
Antigua and Barbuda	0.749886887	3.77(3.21 to 4.51)	0.04	3.74	111 (109)
North Macedonia	0.750629703	2.06(1.44 to 3.16)	0.04	2.02	45 (42)

Location	SDI	Rate of ASDR	Frontier ASDR	Effective difference	Effective difference rank (ASDR rank)
Bahrain	0.753043204	3.71(2.12 to 6.04)	0.04	3.67	108 (106)
Palau	0.754046931	5.14(2.84 to 8.86)	0.04	5.1	157 (156)
Saint Kitts and Nevis	0.754987055	2.51(1.69 to 3.55)	0.04	2.47	62 (60)
Ukraine	0.760773913	3.82(2.53 to 5.52)	0.04	3.78	112 (110)
Bulgaria	0.768150939	2.83(2.09 to 3.75)	0.04	2.79	77 (76)
Romania	0.768453864	2.02(1.39 to 2.8)	0.04	1.98	41 (38)
Trinidad and Tobago	0.768763254	5.83(4.26 to 7.76)	0.04	5.79	167 (166)
Spain	0.769283698	1.63(1.15 to 2.3)	0.04	1.59	24 (21)
Chile	0.771514716	3.67(2.74 to 4.92)	0.04	3.63	106 (105)
Northern Mariana Islands	0.771535213	2.34(1.43 to 3.54)	0.04	2.31	55 (53)
Oman	0.773391602	2.63(1.31 to 4.52)	0.04	2.6	69 (67)
Cook Islands	0.779109955	4.08(2.12 to 7.32)	0.04	4.04	120 (118)
Belarus	0.784484711	2.61(1.73 to 3.86)	0.04	2.57	67 (64)
Hungary	0.790754768	2.11(1.53 to 2.91)	0.04	2.08	48 (44)

Location	SDI	Rate of ASDR	Frontier ASDR	Effective difference	Effective difference rank (ASDR rank)
Greece	0.791854408	2.03(1.64 to 2.52)	0.04	1.99	43 (40)
Serbia	0.792416294	2.01(1.25 to 3.16)	0.04	1.97	40 (37)
Montenegro	0.795800584	3.61(2.52 to 5.17)	0.04	3.57	104 (102)
Croatia	0.798341027	1.86(1.31 to 2.6)	0.04	1.82	33 (32)
Malta	0.801585034	2(1.4 to 2.8)	0.04	1.96	38 (35)
Guam	0.803982203	3.36(2.3 to 4.57)	0.04	3.32	92 (91)
Bahamas	0.805020668	8(6.08 to 10.46)	0.04	7.96	180 (179)
Italy	0.805773534	2.84(2.37 to 3.47)	0.04	2.8	79 (78)
Russian Federation	0.808536005	3.29(2.93 to 3.76)	0.04	3.25	88 (87)
Israel	0.809011652	2.28(1.7 to 3)	0.04	2.24	54 (52)
Brunei Darussalam	0.810234367	3.52(2.15 to 5.51)	0.04	3.48	97 (95)
Slovakia	0.81061053	2.9(1.76 to 4.46)	0.04	2.86	81 (80)
Poland	0.812042809	2.68(2.28 to 3.16)	0.04	2.64	70 (68)
Saudi Arabia	0.815143493	9.71(5.45 to 16.55)	0.04	9.67	188 (186)

**Location**	**SDI**	**Rate of ASDR**	**Frontier ASDR**	**Effective difference**	**Effective difference rank (ASDR rank)**
Bermuda	0.821365422	4.58(3.37 to 6.28)	0.04	4.55	135 (134)
United States Virgin Islands	0.821830853	4.69(2.58 to 7.9)	0.04	4.65	139 (137)
Puerto Rico	0.825525847	4.31(3.08 to 5.93)	0.04	4.28	125 (124)
Greenland	0.826210336	1.85(0.99 to 3.08)	0.04	1.81	31 (30)
Czechia	0.828450433	2.2(1.48 to 3.17)	0.04	2.16	51 (47)
Latvia	0.830663516	2.89(1.98 to 4.07)	0.04	2.86	80 (79)
Cyprus	0.835630545	1.94(1.1 to 3.29)	0.04	1.9	37 (34)
France	0.838364875	3.6(2.38 to 5.16)	0.04	3.56	103 (101)
Slovenia	0.842430731	1.28(0.9 to 1.76)	0.04	1.24	15 (10)
Australia	0.844252814	2.84(1.92 to 4.05)	0.04	2.8	78 (77)
Estonia	0.844917787	2.36(1.63 to 3.24)	0.04	2.32	56 (54)
Kuwait	0.846651055	3.55(2.41 to 5.06)	0.04	3.51	99 (97)
Qatar	0.846860584	3.47(1.98 to 5.73)	0.04	3.43	95 (93)
United Arab Emirates	0.849317734	4.84(2.68 to 7.98)	0.04	4.8	145 (144)

Location	SDI	Rate of ASDR	Frontier ASDR	Effective difference	Effective difference rank (ASDR rank)
New Zealand	0.849442499	2.62(1.93 to 3.44)	0.04	2.59	68 (65)
Belgium	0.853654016	2.45(1.82 to 3.23)	0.04	2.41	59 (57)
Austria	0.853837004	2.73(1.94 to 3.75)	0.04	2.69	73 (71)
Singapore	0.856097766	1.45(1.03 to 2.08)	0.04	1.42	19 (15)
Lithuania	0.856484049	2.69(1.92 to 3.66)	0.04	2.65	71 (69)
United Kingdom	0.859000182	2.16(1.97 to 2.38)	0.04	2.12	50 (46)
Finland	0.859831368	2.03(1.46 to 2.84)	0.04	2	44 (41)
United States of America	0.862448354	3.28(2.88 to 3.79)	0.04	3.24	87 (86)
Andorra	0.869444113	2.36(1.27 to 3.95)	0.04	2.32	57 (55)
Japan	0.871241813	2.12(1.78 to 2.57)	0.04	2.08	49 (45)
Canada	0.87317068	2.6(1.79 to 3.66)	0.04	2.56	66 (63)
Ireland	0.87375385	1.79(1.27 to 2.51)	0.04	1.76	28 (27)
Taiwan (Province of China)	0.874747053	5.53(3.91 to 7.83)	0.04	5.49	162 (160)
Iceland	0.87636168	5.11(3.57 to 7.12)	0.04	5.07	155 (154)

Location	SDI	Rate of ASDR	Frontier ASDR	Effective difference	Effective difference rank (ASDR rank)
Luxembourg	0.884428955	1.86(1.47 to 2.35)	0.04	1.82	32 (31)
Republic of Korea	0.886675267	3.06(1.73 to 5.06)	0.04	3.02	83 (82)
Sweden	0.886880299	1.78(1.36 to 2.28)	0.04	1.74	26 (24)
San Marino	0.888005474	3.55(1.75 to 5.99)	0.04	3.51	100 (98)
Netherlands	0.888464256	2.27(1.66 to 3.08)	0.04	2.23	53 (50)
Denmark	0.896424204	0.92(0.65 to 1.28)	0.04	0.88	10 (5)
Germany	0.902957091	2.49(1.7 to 3.57)	0.04	2.45	61 (59)
Monaco	0.908262831	4.44(2.32 to 7.59)	0.04	4.4	131 (130)
Norway	0.91613281	1.9(1.61 to 2.27)	0.04	1.86	35 (33)
Switzerland	0.933059111	1.16(0.87 to 1.52)	0.04	1.12	14 (8)

Table S9: Incidence of thyroid cancer in adolescents and young adults (AYAs), and their average annual percentage changes from 1990 to 2021 by age at the global level.

Sex	Age	Numbers (thousands), 1990	Age-standardised rate (per 100 000), 1990	Numbers (thousands), 2021	Age-standardised rate (per 100 000), 2021	Total percentage change in number, 1990-2021	AAPC of age-standardised rate, 1990-2021	p value
Both	15 to 19	1.5(1.3 - 1.8)	0.3(0.3 - 0.3)	2.8(2.3 - 3.8)	0.5(0.4 - 0.6)	0.9(0.5 to 1.6)	2.07 (1.90 to 2.24)	<0.001
Both	20 to 24	2.3(2 - 2.7)	0.5(0.4 - 0.6)	5.2(4.3 - 7)	0.9(0.7 - 1.2)	1.2(0.9 to 2)	2.63 (2.50 to 2.76)	<0.001
Both	25 to 29	3.5(3.1 - 4.2)	0.8(0.7 - 0.9)	8.4(7 - 10.5)	1.4(1.2 - 1.8)	1.4(1.1 to 2)	2.89 (2.69 to 3.10)	<0.001
Both	30 to 34	5(4.5 - 5.7)	1.3(1.2 - 1.5)	13.7(11.6 - 16.5)	2.3(1.9 - 2.7)	1.7(1.4 to 2.3)	3.36 (3.19 to 3.52)	<0.001
Both	35 to 39	6.9(6.3 - 7.7)	2(1.8 - 2.2)	18(15.6 - 20.6)	3.2(2.8 - 3.7)	1.6(1.3 to 2)	3.09 (2.88 to 3.30)	<0.001
Female	15 to 19	1.1(0.9 - 1.4)	0.4(0.4 - 0.5)	2.1(1.6 - 3)	0.7(0.5 - 1)	0.9(0.5 to 1.9)	2.19 (1.99 to 2.38)	<0.001
Female	20 to 24	1.8(1.5 - 2.2)	0.7(0.6 - 0.9)	4(3.1 - 5.7)	1.4(1.1 - 1.9)	1.2(0.7 to 2.2)	2.64 (2.45 to 2.82)	<0.001
Female	25 to 29	2.6(2.2 - 3.2)	1.2(1 - 1.5)	6.1(4.8 - 8)	2.1(1.6 - 2.7)	1.3(0.9 to 2.1)	2.74 (2.37 to 3.11)	<0.001
Female	30 to 34	3.9(3.4 - 4.5)	2(1.8 - 2.4)	10(8.1 - 12.7)	3.3(2.7 - 4.3)	1.6(1.2 to 2.3)	3.15 (2.97 to 3.32)	<0.001
Female	35 to 39	5.2(4.6 - 5.9)	3(2.6 - 3.4)	12.7(10.6 - 15.4)	4.6(3.8 - 5.5)	1.4(1.1 to 1.9)	2.83 (2.75 to 2.91)	<0.001
Male	15 to 19	0.4(0.4 - 0.5)	0.2(0.1 - 0.2)	0.7(0.6 - 0.8)	0.2(0.2 - 0.3)	0.7(0.2 to 1.1)	1.80 (1.63 to 1.97)	<0.001
Male	20 to 24	0.6(0.5 - 0.6)	0.2(0.2 - 0.3)	1.2(1 - 1.4)	0.4(0.3 - 0.5)	1.2(0.7 to 1.6)	2.58 (2.39 to 2.78)	<0.001
Male	25 to 29	0.9(0.8 - 0.9)	0.4(0.4 - 0.4)	2.3(1.9 - 2.7)	0.8(0.6 - 0.9)	1.7(1.2 to 2)	3.23 (3.03 to 3.43)	<0.001
Male	30 to 34	1.2(1.1 - 1.3)	0.6(0.5 - 0.6)	3.8(3.1 - 4.3)	1.2(1 - 1.4)	2.3(1.8 to 2.7)	3.91 (3.71 to 4.10)	<0.001
Male	35 to 39	1.7(1.6 - 1.9)	1(0.9 - 1)	5.2(4.4 - 5.9)	1.8(1.6 - 2.1)	2.1(1.6 to 2.4)	3.63 (3.29 to 3.98)	<0.001

Data in parentheses are 95% uncertainty intervals for numbers, total percentage change, and 95% CIs for age-standardised rates, and AAPCs. DALY=disability-adjusted life-year.

Table S10: Prevalence of thyroid cancer in adolescents and young adults (AYAs) , and their average annual percentage changes from 1990 to 2021 by age at the global level.

Sex	Age	Numbers (thousands), 1990	Age-standardised rate (per 100 000), 1990	Numbers (thousands), 2021	Age-standardised rate (per 100 000), 2021	Total percentage change in number, 1990-2021	AAPC of age-standardised rate, 1990-2021	p value
Both	15 to 19	13.4(11.9 - 15.8)	2.6(2.3 - 3)	25.7(20.7 - 34.3)	4.1(3.3 - 5.5)	0.9(0.5 to 1.6)	2.22 (2.03 to 2.41)	<0.001
Both	20 to 24	20.7(18 - 24.1)	4.2(3.7 - 4.9)	47(38.5 - 62.9)	7.9(6.4 - 10.5)	1.3(0.9 to 2)	2.69 (2.50 to 2.87)	<0.001
Both	25 to 29	31(27.4 - 37.2)	7(6.2 - 8.4)	76.3(63.8 - 94.7)	13(10.8 - 16.1)	1.5(1.1 to 2.1)	2.78 (2.41 to 3.14)	<0.001
Both	30 to 34	45.1(40.6 - 51.2)	11.7(10.5 - 13.3)	124.6(104.8 - 149.5)	20.6(17.3 - 24.7)	1.8(1.4 to 2.3)	3.18 (3.00 to 3.36)	<0.001
Both	35 to 39	62.1(56.3 - 68.6)	17.6(16 - 19.5)	162.6(141.2 - 186.6)	29(25.2 - 33.3)	1.6(1.3 to 2)	2.86 (2.78 to 2.94)	<0.001
Female	15 to 19	9.9(8.3 - 12.2)	3.9(3.2 - 4.8)	19.3(14.8 - 27.4)	6.4(4.9 - 9)	1(0.5 to 1.9)	2.13 (1.96 to 2.30)	<0.001
Female	20 to 24	15.8(13 - 19.3)	6.5(5.3 - 7.9)	36(27.9 - 51.6)	12.2(9.5 - 17.6)	1.3(0.8 to 2.2)	2.69 (2.56 to 2.81)	<0.001
Female	25 to 29	23.4(20 - 28.9)	10.6(9.1 - 13.2)	55.4(43.6 - 72.6)	19(15 - 24.9)	1.4(0.9 to 2.1)	2.93 (2.73 to 3.13)	<0.001
Female	30 to 34	34.9(30.6 - 40.9)	18.3(16.1 - 21.5)	90.8(73.3 - 115.9)	30.4(24.5 - 38.8)	1.6(1.2 to 2.3)	3.39 (3.23 to 3.55)	<0.001
Female	35 to 39	47(41.1 - 53.1)	27.1(23.7 - 30.6)	115.6(96.3 - 139.9)	41.6(34.7 - 50.4)	1.5(1.1 to 1.9)	3.13 (2.92 to 3.34)	<0.001
Male	15 to 19	3.6(3.2 - 4.1)	1.3(1.2 - 1.6)	6.3(5.1 - 7.5)	2(1.6 - 2.3)	0.8(0.3 to 1.2)	1.88 (1.71 to 2.06)	<0.001
Male	20 to 24	4.9(4.5 - 5.5)	2(1.8 - 2.2)	11.1(9.1 - 12.8)	3.7(3 - 4.2)	1.3(0.8 to 1.6)	2.67 (2.47 to 2.87)	<0.001
Male	25 to 29	7.6(6.9 - 8.4)	3.4(3.1 - 3.8)	20.8(17.1 - 23.9)	7(5.8 - 8)	1.7(1.2 to 2.1)	3.29 (3.09 to 3.49)	<0.001
Male	30 to 34	10.2(9.3 - 11.1)	5.2(4.8 - 5.7)	33.8(28.3 - 38.7)	11.1(9.3 - 12.7)	2.3(1.8 to 2.7)	3.96 (3.76 to 4.16)	<0.001
Male	35 to 39	15.1(13.9 - 16.4)	8.4(7.8 - 9.2)	47(39.7 - 52.9)	16.6(14 - 18.7)	2.1(1.7 to 2.5)	3.68 (3.42 to 3.94)	<0.001

Data in parentheses are 95% uncertainty intervals for numbers, total percentage change, and 95% CIs for age-standardised rates, and AAPCs. DALY=disability-adjusted life-year.

Table S11: DALYs of thyroid cancer in adolescents and young adults (AYAs), and their average annual percentage changes from 1990 to 2021 by age at the global level.

Sex	Age	Numbers (thousands), 1990	Age-standardised rate (per 100 000), 1990	Numbers (thousands), 2021	Age-standardised rate (per 100 000), 2021	Total percentage change in number, 1990-2021	AAPC of age-standardised rate, 1990-2021	p value
Both	15 to 19	13.6(12 - 16.3)	2.6(2.3 - 3.1)	17.4(13.8 - 23.9)	2.8(2.2 - 3.8)	0.3(0 to 0.8)	0.83 (0.67 to 0.99)	<0.001
Both	20 to 24	20(16.9 - 23.8)	4.1(3.4 - 4.8)	29.7(23.2 - 40.8)	5(3.9 - 6.8)	0.5(0.2 to 1)	1.29 (1.05 to 1.53)	<0.001
Both	25 to 29	24.8(20.9 - 30.7)	5.6(4.7 - 6.9)	39.5(32.2 - 50.9)	6.7(5.5 - 8.7)	0.6(0.3 to 1.1)	1.47 (1.18 to 1.77)	<0.001
Both	30 to 34	24.9(21.8 - 29.8)	6.5(5.7 - 7.7)	44.2(36.7 - 54.7)	7.3(6.1 - 9.1)	0.8(0.5 to 1.2)	1.88 (1.78 to 1.99)	<0.001
Both	35 to 39	31.4(27.5 - 36.3)	8.9(7.8 - 10.3)	52.6(44 - 62)	9.4(7.8 - 11.1)	0.7(0.5 to 1)	1.67 (1.43 to 1.92)	<0.001
Female	15 to 19	9(7.1 - 11.6)	3.5(2.8 - 4.5)	12.1(8.8 - 18.1)	4(2.9 - 5.9)	0.4(0 to 1.1)	1.00 (0.72 to 1.28)	<0.001
Female	20 to 24	13.9(10.8 - 17.8)	5.7(4.4 - 7.3)	20.8(15.4 - 31.5)	7.1(5.2 - 10.7)	0.5(0.1 to 1.2)	1.35 (1.05 to 1.65)	<0.001
Female	25 to 29	16.9(13.4 - 22.5)	7.7(6.1 - 10.2)	25.9(19.4 - 36.3)	8.9(6.7 - 12.5)	0.5(0.2 to 1.2)	1.39 (1.12 to 1.66)	<0.001
Female	30 to 34	17.1(14.1 - 21.8)	9(7.4 - 11.5)	28.2(21.9 - 37.5)	9.4(7.3 - 12.6)	0.6(0.3 to 1.2)	1.62 (1.47 to 1.76)	<0.001
Female	35 to 39	20.9(17.1 - 25.4)	12.1(9.9 - 14.6)	32.2(25.4 - 41.8)	11.6(9.1 - 15.1)	0.5(0.3 to 0.9)	1.35 (1.00 to 1.71)	<0.001
Male	15 to 19	4.6(3.9 - 5.5)	1.8(1.5 - 2.1)	5.3(4.1 - 6.4)	1.7(1.3 - 2)	0.1(-0.2 to 0.5)	0.43 (0.33 to 0.53)	<0.001
Male	20 to 24	6.1(5.3 - 7.3)	2.5(2.2 - 2.9)	8.9(6.9 - 10.6)	2.9(2.3 - 3.5)	0.5(0.1 to 0.8)	1.19 (1.08 to 1.30)	<0.001
Male	25 to 29	7.9(7 - 9)	3.5(3.1 - 4.1)	13.6(10.7 - 16.4)	4.6(3.6 - 5.5)	0.7(0.4 to 1)	1.76 (1.61 to 1.90)	<0.001
Male	30 to 34	7.8(7 - 8.9)	4(3.6 - 4.6)	16(13 - 18.8)	5.2(4.3 - 6.2)	1.1(0.7 to 1.3)	2.37 (2.14 to 2.60)	<0.001
Male	35 to 39	10.5(9.4 - 12)	5.9(5.2 - 6.7)	20.4(16.5 - 23.6)	7.2(5.8 - 8.3)	0.9(0.6 to 1.2)	2.15 (1.85 to 2.44)	<0.001

Data in parentheses are 95% uncertainty intervals for numbers, percentage change, and 95% CIs for age-standardised rates, and AAPCs. DALY=disability-adjusted life-year. AAPC=average annual percentage change.

Table S12: Decomposition of the percentage changes in thyroid cancer among adolescents and young adults (AYAs) worldwide from 1990 to 2021, alterations in incidence as influenced by aging, population growth, and epidemiological change.

Location	Overall difference	Population aging	Population growth	Epidemiological change	Percent change of population aging (%)	Percent change of population growth (%)	Percent change of epidemiological change (%)	Overall percent change (%)
Global	28935.1	2352.6	9728.1	16854.3	12.2	50.5	87.5	150.2
High SDI	2847.6	422.1	126.2	2299.4	7.5	2.2	40.7	50.4
High-middle SDI	3427.3	862.4	-168.3	2733.2	19.4	-3.8	61.4	77.0
Middle SDI	10869.5	1312.8	2035.9	7520.8	26.4	40.9	151.2	218.5
Low-middle SDI	7863.1	317.1	3423.4	4122.6	11.0	118.9	143.2	273.2
Low SDI	3917.4	-3.3	2460.7	1460.1	-0.3	190.9	113.3	303.9
Andean Latin America	296.2	24.6	118.5	153.1	25.7	123.6	159.7	308.9
Australasia	107.5	10.7	42.2	54.6	8.8	34.9	45.1	88.8
Caribbean	79.1	14.6	27.5	37.0	14.6	27.5	37.0	79.1
Central Asia	61.9	32.7	69.2	-40.0	14.6	31.0	-17.9	27.7
Central Europe	-259.8	49.7	-160.4	-149.2	7.2	-23.2	-21.6	-37.7
Central Latin America	803.9	87.5	288.0	428.4	22.0	72.4	107.6	202.0
Central Sub-Saharan Africa	77.6	0.8	57.3	19.4	2.8	190.3	64.4	257.5
East Asia	4530.4	1180.0	-971.7	4322.1	33.6	-27.7	123.1	129.1
Eastern Europe	193.5	126.7	-308.3	375.1	11.8	-28.7	34.9	18.0

Location	Overall difference	Population aging	Population growth	Epidemiological change	Percent change of population aging (%)	Percent change of population growth (%)	Percent change of epidemiological change (%)	Overall percent change (%)
Eastern Sub-Saharan Africa	2158.8	14.1	1430.0	714.6	1.9	187.5	93.7	283.0
High-income Asia Pacific	245.0	109.2	-298.9	434.7	12.3	-33.7	49.0	27.6
High-income North America	1124.9	-44.8	193.5	976.2	-2.5	10.9	55.1	63.5
North Africa and Middle East	4660.7	487.4	1931.1	2242.2	38.6	153.1	177.8	369.6
Oceania	19.3	1.5	13.9	3.9	14.0	129.9	36.7	180.6
South Asia	10602.2	321.8	4471.5	5808.9	9.9	137.6	178.8	326.4
Southeast Asia	3425.7	380.6	1133.8	1911.3	20.3	60.6	102.2	183.1
Southern Latin America	146.1	14.3	68.5	63.4	8.8	42.3	39.1	90.2
Southern Sub-Saharan Africa	150.7	25.2	83.7	41.8	20.9	69.6	34.7	125.2
Tropical Latin America	451.9	59.9	145.9	246.2	22.4	54.7	92.2	169.3
Western Europe	-189.3	175.7	-259.4	-105.6	6.8	-10.1	-4.1	-7.4
Western Sub-Saharan Africa	248.7	-1.1	183.0	66.7	-1.2	199.8	72.8	271.5

Table S13: Decomposition of the percentage changes in thyroid cancer among adolescents and young adults (AYAs) worldwide from 1990 to 2021, alterations in prevalence as influenced by aging, population growth, and epidemiological change.

Location	Overall difference	Population aging	Population growth	Epidemiological change	Percent change of population aging (%)	Percent change of population growth (%)	Percent change of epidemiological change (%)	Overall percent change (%)
Global	263880.5	21289.7	87641.2	154949.6	12.4	50.9	89.9	153.2
High SDI	26245.5	3851.1	1152.2	21242.2	7.5	2.2	41.2	51.0
High-middle SDI	31674.8	7832.2	-1528.5	25371.1	19.5	-3.8	63.2	78.9
Middle SDI	99498.2	11861.5	18371.1	69265.6	26.8	41.5	156.4	224.7
Low SDI	35293.9	-30.1	21576.0	13748.0	-0.3	195.7	124.7	320.1
Low-middle SDI	71075.2	2826.5	30393.0	37855.7	11.3	121.1	150.8	283.1
Andean Latin America	2703.6	221.5	1063.1	1419.1	26.2	125.9	168.0	320.1
Australasia	987.0	97.6	385.0	504.4	8.9	35.0	45.8	89.7
Caribbean	720.4	131.3	246.7	342.4	14.7	27.6	38.3	80.6
Central Asia	578.3	294.1	624.1	-339.9	14.7	31.1	-17.0	28.9
Central Europe	-2307.4	450.7	-1455.6	-1302.5	7.2	-23.4	-20.9	-37.0
Central Latin America	7323.0	787.1	2585.8	3950.1	22.3	73.2	111.8	207.3
Central Sub-Saharan Africa	694.6	7.4	499.4	187.9	2.9	194.2	73.1	270.1
East Asia	41933.2	10708.3	-8805.7	40030.6	34.2	-28.1	127.7	133.7
Eastern Europe	1775.4	1145.1	-2794.8	3425.1	11.8	-28.7	35.2	18.2
Eastern Sub-Saharan Africa	19443.5	122.4	12475.8	6845.2	1.9	192.5	105.6	300.0

Location	Overall difference	Population aging	Population growth	Epidemiological change	Percent change of population aging (%)	Percent change of population growth (%)	Percent change of epidemiological change (%)	Overall percent change (%)
High-income Asia Pacific	2287.5	1000.6	-2737.7	4024.6	12.3	-33.8	49.6	28.2
High-income North America	10303.3	-409.0	1766.7	8945.5	-2.5	10.9	55.4	63.8
North Africa and Middle East	42636.2	4438.1	17595.9	20602.1	38.8	153.8	180.1	372.8
Oceania	173.2	13.3	123.3	36.6	14.1	130.7	38.8	183.5
South Asia	95864.1	2867.3	39704.4	53292.3	10.2	140.6	188.7	339.4
Southeast Asia	31363.0	3435.0	10225.3	17702.7	20.6	61.3	106.1	188.0
Southern Latin America	1344.6	129.3	619.4	596.0	8.9	42.6	40.9	92.4
Southern Sub-Saharan Africa	1312.2	217.6	731.7	362.9	20.7	69.5	34.4	124.6
Tropical Latin America	4119.7	538.2	1309.8	2271.7	22.7	55.2	95.8	173.7
Western Europe	-1606.6	1604.7	-2369.7	-841.5	6.9	-10.1	-3.6	-6.9
Western Sub-Saharan Africa	2231.8	-9.5	1612.5	628.8	-1.2	202.7	79.0	280.5

Table S14: Decomposition of the percentage changes in thyroid cancer among adolescents and young adults (AYAs) worldwide from 1990 to 2021, alterations in DALYs as influenced by aging, population growth, and epidemiological change.

Location	Overall difference	Population aging	Population growth	Epidemiological change	Percent change of population aging (%)	Percent change of population growth (%)	Percent change of epidemiological change (%)	Overall percent change (%)
Global	68762.5	6105.8	44634.1	18022.6	5.3	38.9	15.7	59.9
High SDI	-1147.0	631.7	236.0	-2014.8	4.6	1.7	-14.7	-8.4
High-middle SDI	-2987.0	1966.0	-506.9	-4446.0	9.9	-2.5	-22.3	-15.0
Middle SDI	16874.6	4005.3	8757.0	4112.4	11.7	25.7	12.0	49.4
Low-middle SDI	33227.4	1549.1	25129.8	6548.5	5.2	84.4	22.0	111.6
Low SDI	22790.6	-31.8	24030.4	-1208.0	-0.2	140.6	-7.1	133.4
Andean Latin America	689.3	102.9	665.1	-78.8	11.7	75.9	-9.0	78.6
Australasia	43.1	15.0	74.9	-46.7	5.4	26.9	-16.8	15.5
Caribbean	225.8	59.3	151.3	15.2	9.3	23.6	2.4	35.2
Central Asia	-263.1	140.9	370.1	-774.1	9.8	25.7	-53.7	-18.3
Central Europe	-1982.7	145.4	-537.0	-1591.1	5.0	-18.3	-54.2	-67.6
Central Latin America	1727.0	333.3	1473.5	-79.8	11.3	49.9	-2.7	58.4
Central Sub-Saharan Africa	571.2	6.2	638.1	-73.1	1.5	153.4	-17.6	137.3
East Asia	-3359.2	2966.0	-3296.8	-3028.4	13.8	-15.3	-14.1	-15.6
Eastern Europe	-857.0	254.5	-781.1	-330.4	7.3	-22.5	-9.5	-24.7
Eastern Sub-Saharan Africa	13841.7	93.3	15358.8	-1610.4	0.9	141.2	-14.8	127.3

Location	Overall difference	Populati on aging	Population growth	Epidemiological change	Percent change of population aging (%)	Percent change of population growth (%)	Percent change of epidemiological change (%)	Overall percent change (%)
High-income Asia Pacific	-580.1	123.5	-462.1	-241.5	6.5	-24.2	-12.6	-30.4
High-income North America	921.2	-62.0	307.7	675.5	-1.9	9.5	20.9	28.5
North Africa and Middle East	6730.3	933.2	4926.1	871.0	19.1	100.9	17.8	137.9
Oceania	98.2	8.3	90.3	-0.4	10.6	115.8	-0.6	125.9
South Asia	45258.8	1596.3	33456.2	10206.3	4.4	92.7	28.3	125.4
Southeast Asia	5281.3	1224.9	4827.7	-771.3	10.5	41.4	-6.6	45.3
Southern Latin America	48.9	48.1	288.5	-287.7	5.2	31.4	-31.3	5.3
Southern Sub-Saharan Africa	934.3	136.6	624.2	173.5	14.2	64.7	18.0	96.9
Tropical Latin America	1066.3	241.6	799.4	25.3	11.9	39.4	1.2	52.6
Western Europe	-3085.5	275.3	-517.5	-2843.4	4.2	-7.9	-43.5	-47.2
Western Sub-Saharan Africa	1452.9	-7.8	1625.3	-164.5	-0.8	159.4	-16.1	142.5

Table S15: Prediction results of Bayesian age–period–cohort analysis (BAPC) in thyroid cancer among adolescents and young adults (AYAs) worldwide in 2040.

Location	Incidence		Prevalence		DALYs	
	Numbers (thousands), 2040	Age-standardised rate (per 100,000 population), 2040	Numbers (thousands), 2040	Age-standardised rate (per 100,000 population), 2040	Numbers (thousands), 2040	Age-standardised rate (per 100,000 population), 2040
Global	60.2	0.7	558.4	17.3	199.7	6.2
male	15.4	0.8	128.9	7.7	60.2	3.6
female	46.2	2.9	436.8	27.8	142.8	9.1
Andean Latin America	0.5	1.9	2.4	8.1	1.3	4.4
Australasia	0.1	2.1	1.1	9.7	0.2	1.4
Caribbean	0.2	1.1	1.2	7.1	0.7	4.2
Central Asia	0.2	0.7	2.4	5.8	0.9	2.2
Central Europe	0.2	0.8	1.3	4.8	0.3	1.1
Central Latin America	1.8	1.8	12.4	10.9	4.1	3.6
Central Sub-Saharan Africa	0.4	0.8	2.9	3.2	1.9	2.1
East Asia	6.2	1.6	59.3	15.7	10.0	2.6
Eastern Europe	1.1	1.3	11.4	20.0	2.2	3.8
Eastern Sub-Saharan Africa	8.9	1.2	79.4	27.0	45.0	15.3
High-income Asia Pacific	0.8	1.9	9.6	23.7	1.0	2.5
High-income North America	1.4	0.5	19.4	15.5	2.1	1.7
North Africa and Middle East	6.3	2.1	55.6	18.2	10.7	3.5
Oceania	0.0	2.9	0.5	6.2	0.3	3.5

Location	Incidence		Prevalence		DALYs	
	Numbers (thousands), 2040	Age-standardised rate (per 100,000 population), 2040	Numbers (thousands), 2040	Age-standardised rate (per 100,000 population), 2040	Numbers (thousands), 2040	Age-standardised rate (per 100,000 population), 2040
South Asia	23.8	2.6	244.2	29.7	95.2	11.6
Southeast Asia	7.0	1.6	62.8	23.2	16.1	5.9
Southern Latin America	0.3	0.5	1.5	6.0	0.5	2.0
Southern Sub-Saharan Africa	0.7	0.2	4.9	12.3	3.2	8.1
Tropical Latin America	0.6	3.0	6.0	7.5	2.0	2.5
Western Europe	0.8	0.4	8.6	6.9	1.3	1.0
Western Sub-Saharan Africa	0.8	1.8	7.1	2.1	3.8	1.2

Figure S1. Cases and age-standardized rates of incidence at the regional level in 1990 and 2021. (A) Incidence cases; (B) Age-standardized incidence rates.

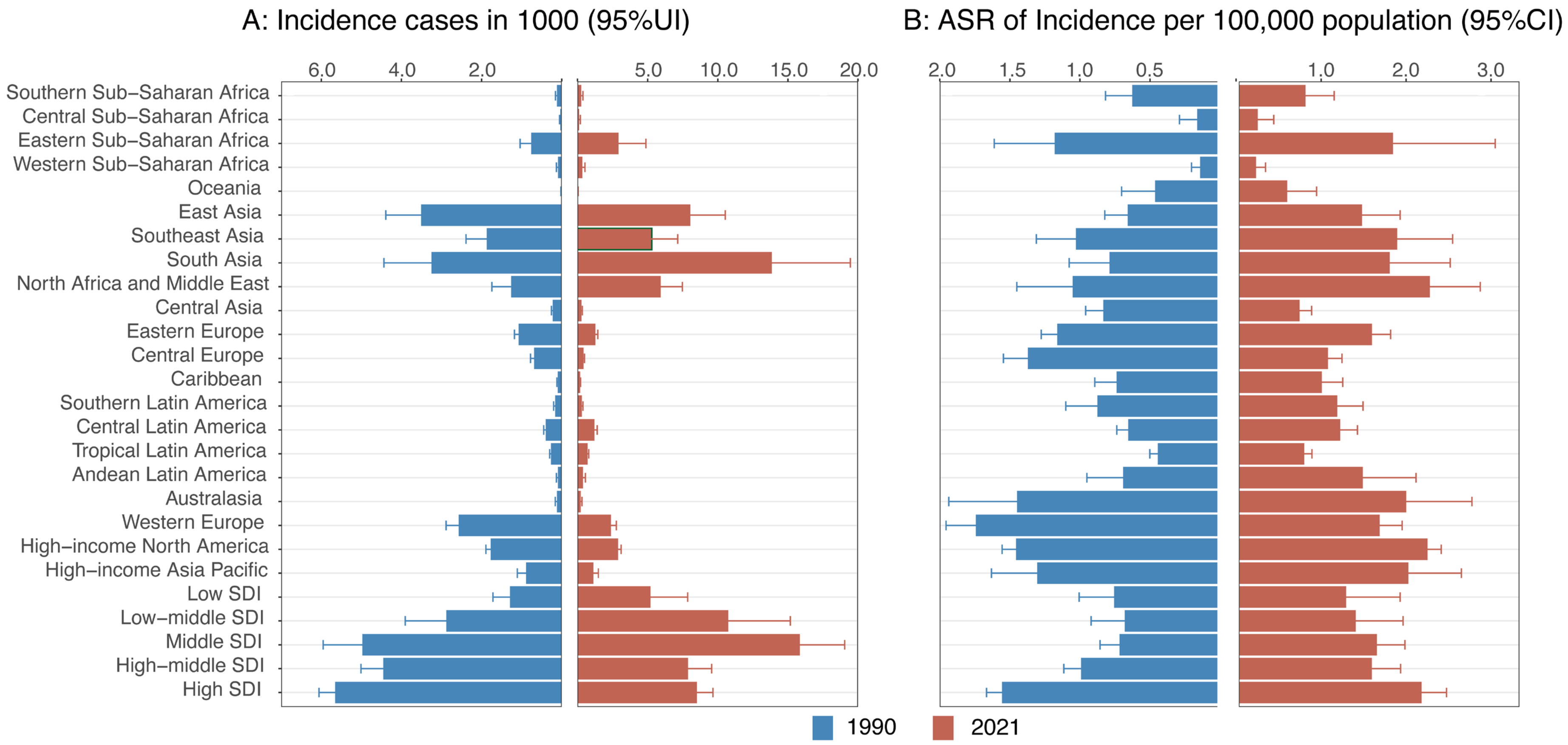

Fgiure S2: The APCs of ASR for thyroid cancer among adolescents and young adults (AYAs) at the global and regional level based on the Joinpoint regression analysis model. (A) Incidence cases; (B) Prevalence cases; (C) DALYs cases. DALYs= disability-adjusted life-years.

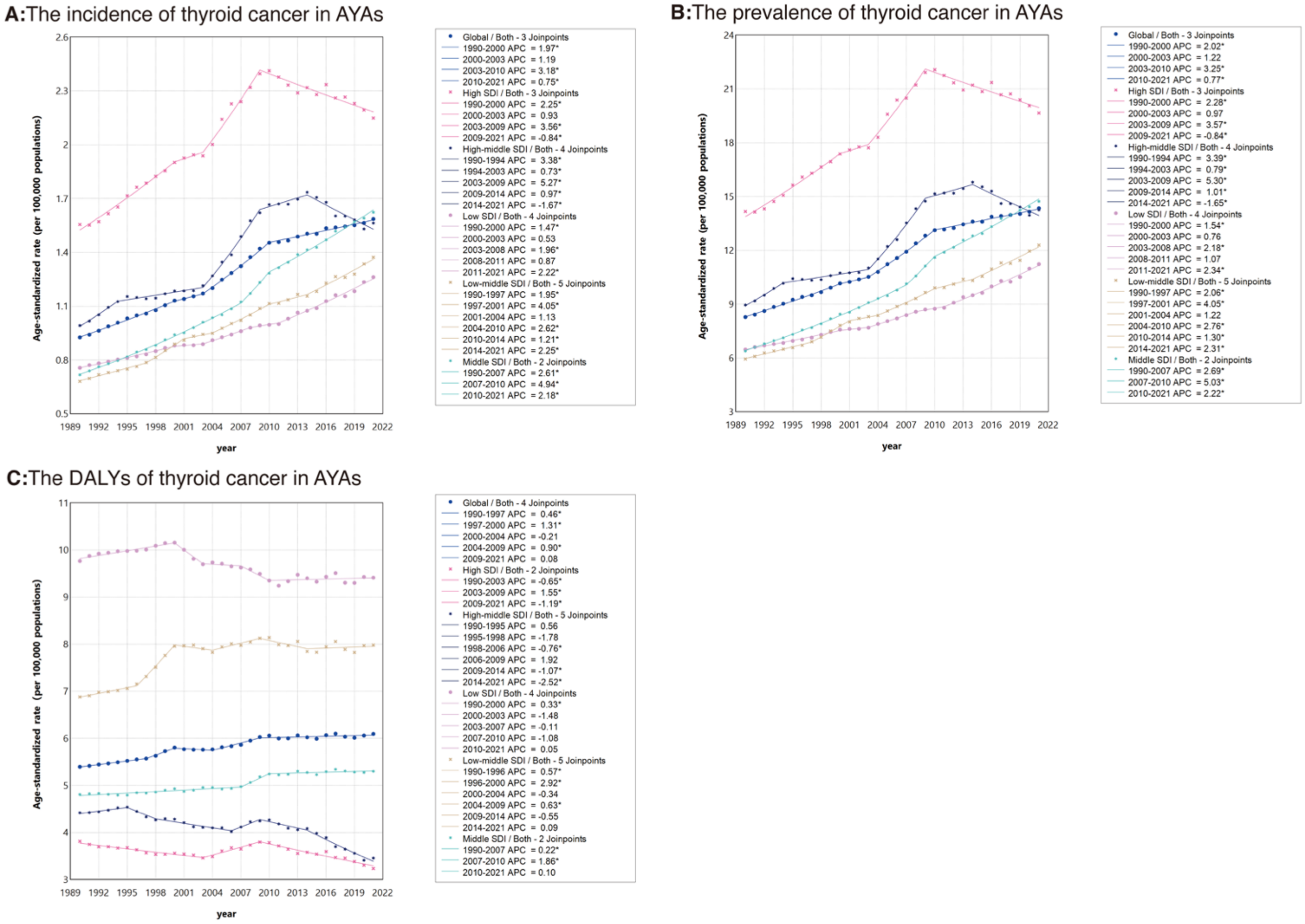

Figure S3. Cases and age-standardized rates of prevalence at the regional level in 1990 and 2021. (A) Prevalence cases; (B) Age-standardized prevalence rates.

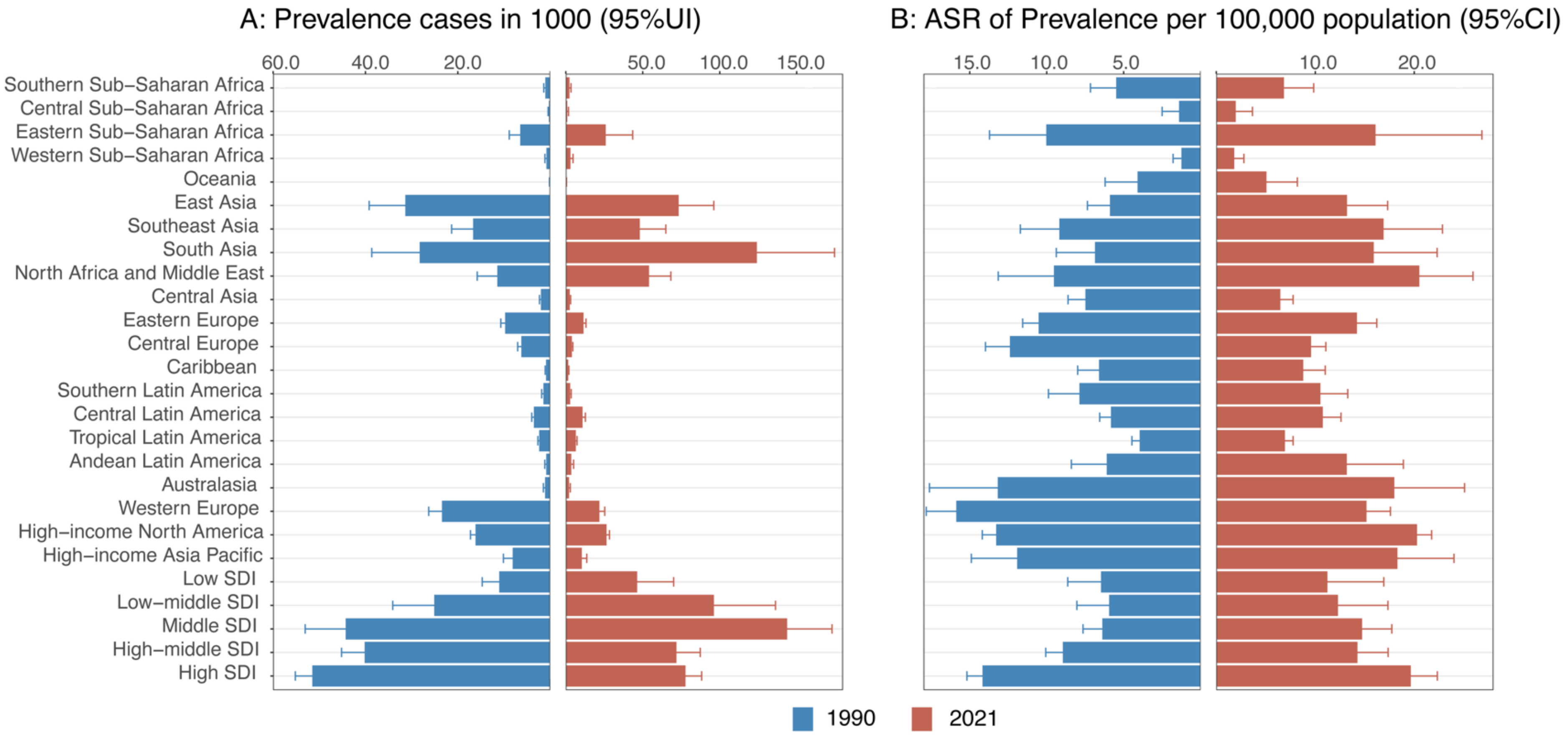

Figure S4. Cases and age-standardized rates of DALYs at the regional level in 1990 and 2021. (A) DALYs cases; (B) Age-standardized DALYs rates. DALYs=disability-adjusted life-years.

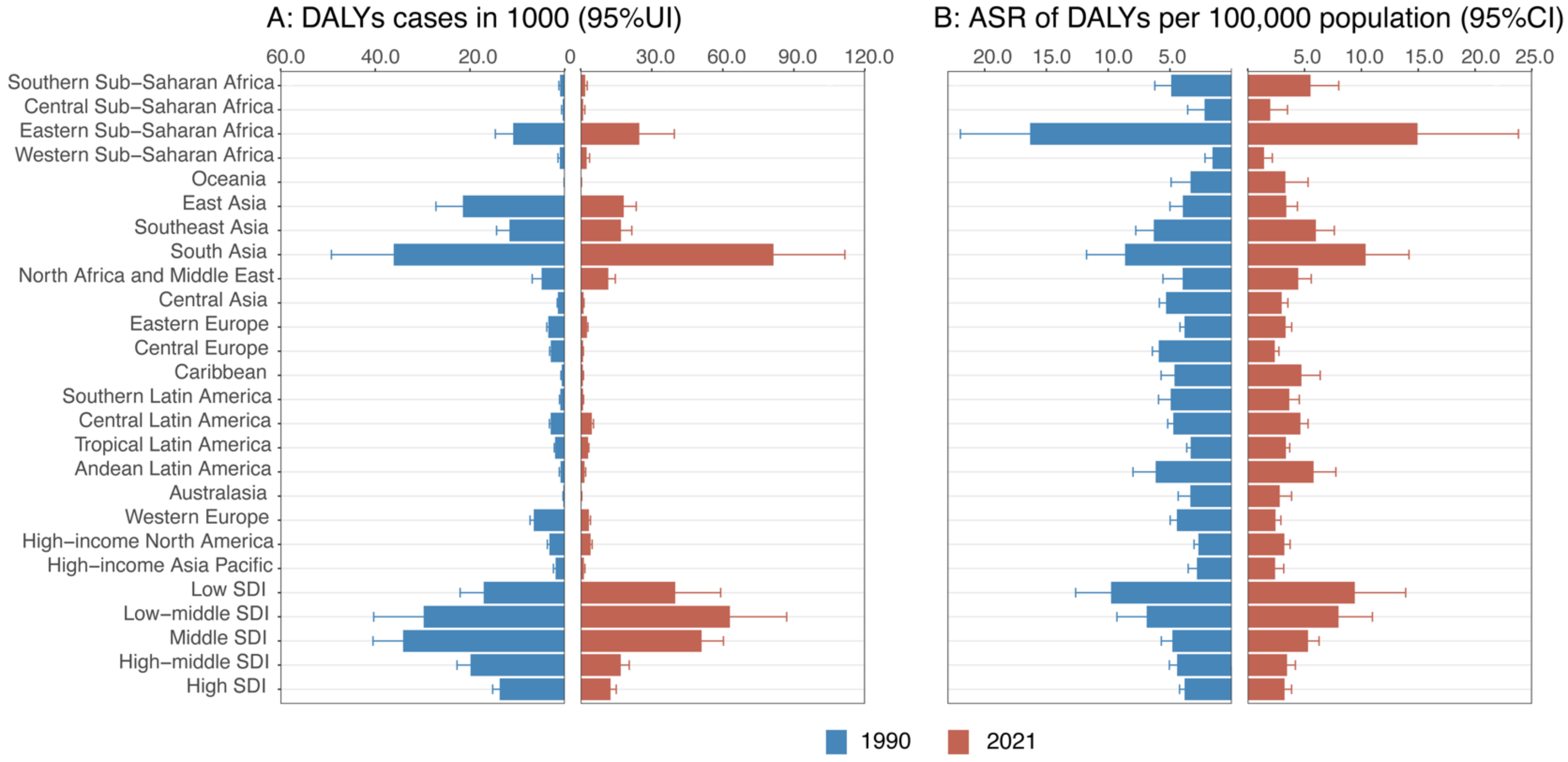

Figure S5. Numbers of incidence (thousand) for thyroid cancer among adolescents and young adults at global level in 2021. From outermost to innermost, the layers are number of incidence (thousand), 5 SDI regions, 21 GBD regions, and 5 GBD super regions. The ISO-3 codes were utilized to identify the 204 countries and territories, with their full names detailed in S4 Table within S1 File of the updated manuscript. ASIR= Age-standardized incidence rates. SDI= Sociodemographic index.

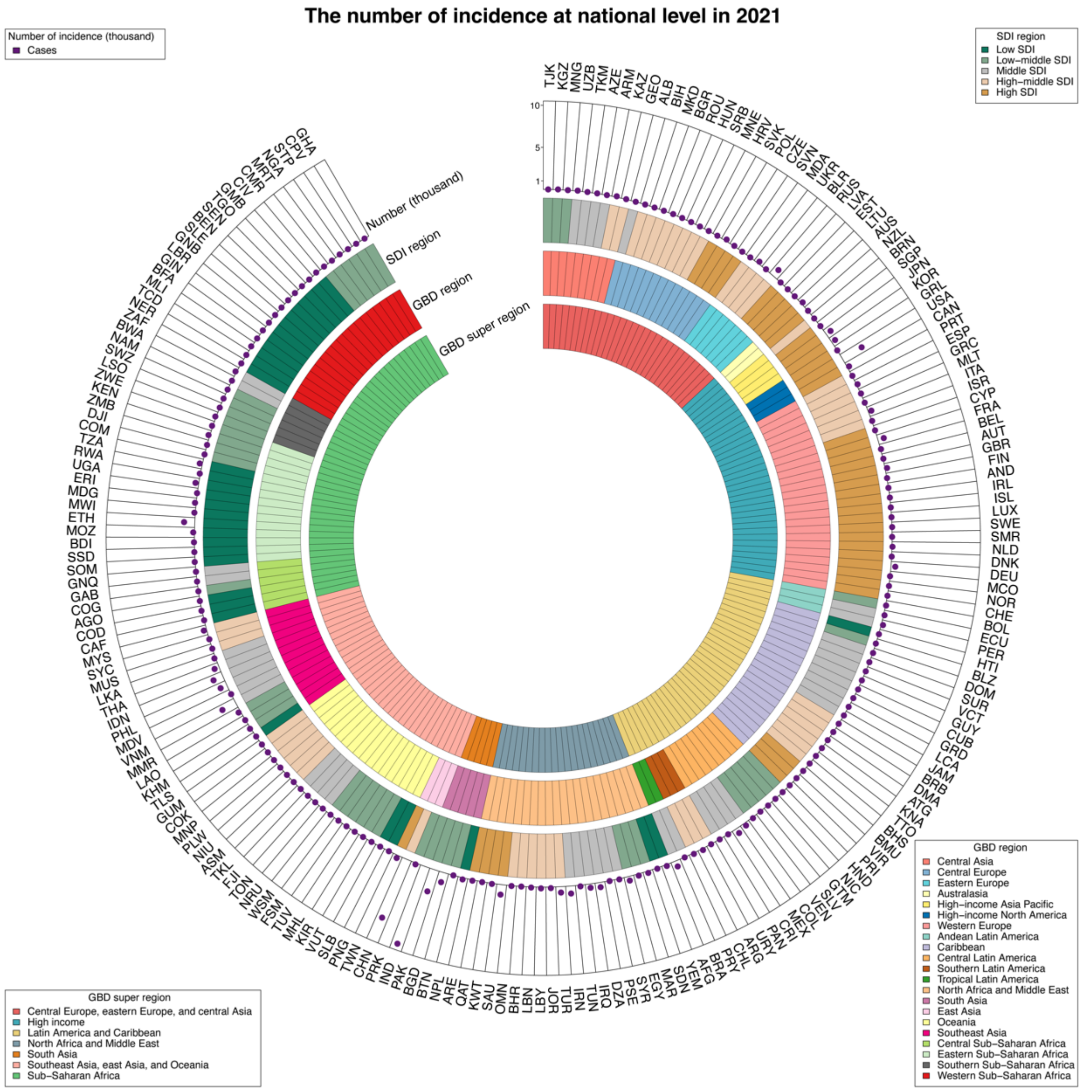

Figure S6. Age-standardized rates for thyroid cancer in adolescents and young adults by SDI across 204 countries/territories in 2021. (A), Age-standardized incidence rates; (B), Age-standardized prevalence rates; (C), Age-standardized DALYs rates. SDI= Sociodemographic index. DALYs=disability-adjusted life-years.

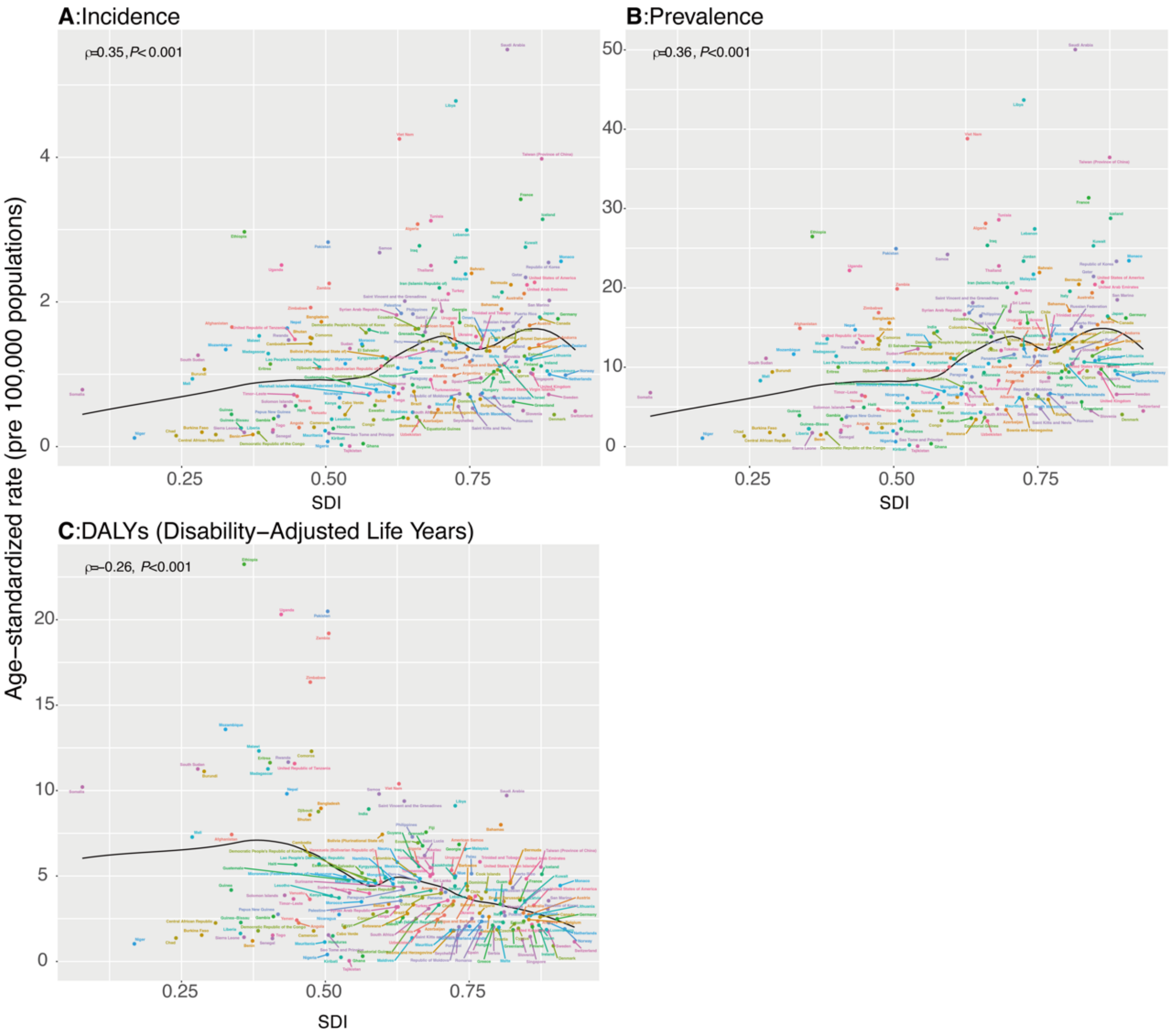

Figure S7. Number of prevalence (thousand) for thyroid cancer among adolescents and young adults at global level in 2021. From outermost to innermost, the layers are number of prevalence (thousand), 5 SDI regions, 21 GBD regions, and 5 GBD super regions. The ISO-3 codes were utilized to identify the 204 countries and territories, with their full names detailed in S5 Table within S1 File of the updated manuscript. ASPR= Age-standardized prevalence rates. SDI= Sociodemographic index.

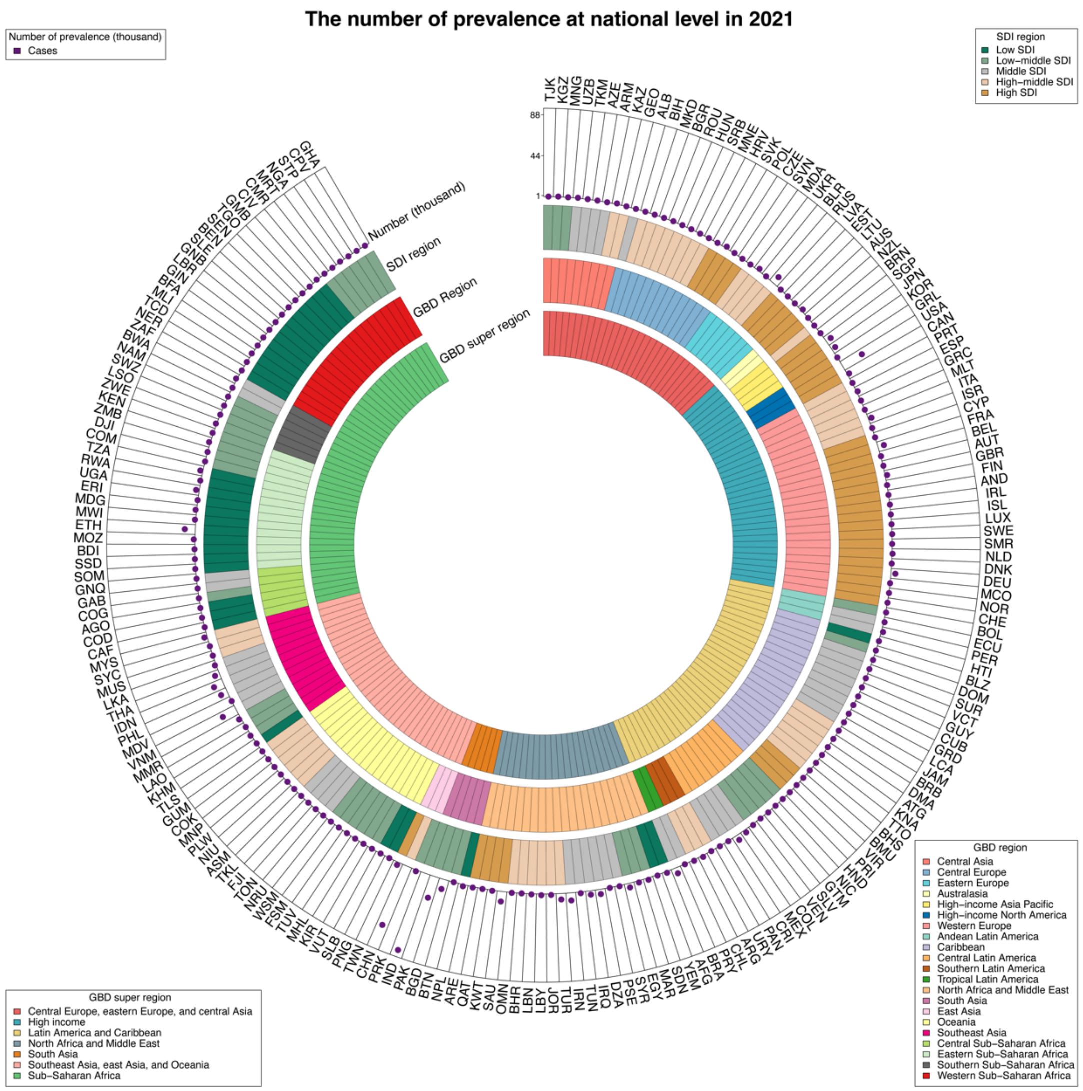

Figure S8. Number of DALYs (thousand) for thyroid cancer among adolescents and young adults at global level in 2021. From outermost to innermost, the layers are number of DALYs (thousand), 5 SDI regions, 21 GBD regions, and 5 GBD super regions. The ISO-3 codes were utilized to identify the 204 countries and territories, with their full names detailed in S6 Table within S1 File of the updated manuscript. ASDR= Age-standardized DALYs rates. DALY=disability-adjusted life-year. SDI= Sociodemographic index.

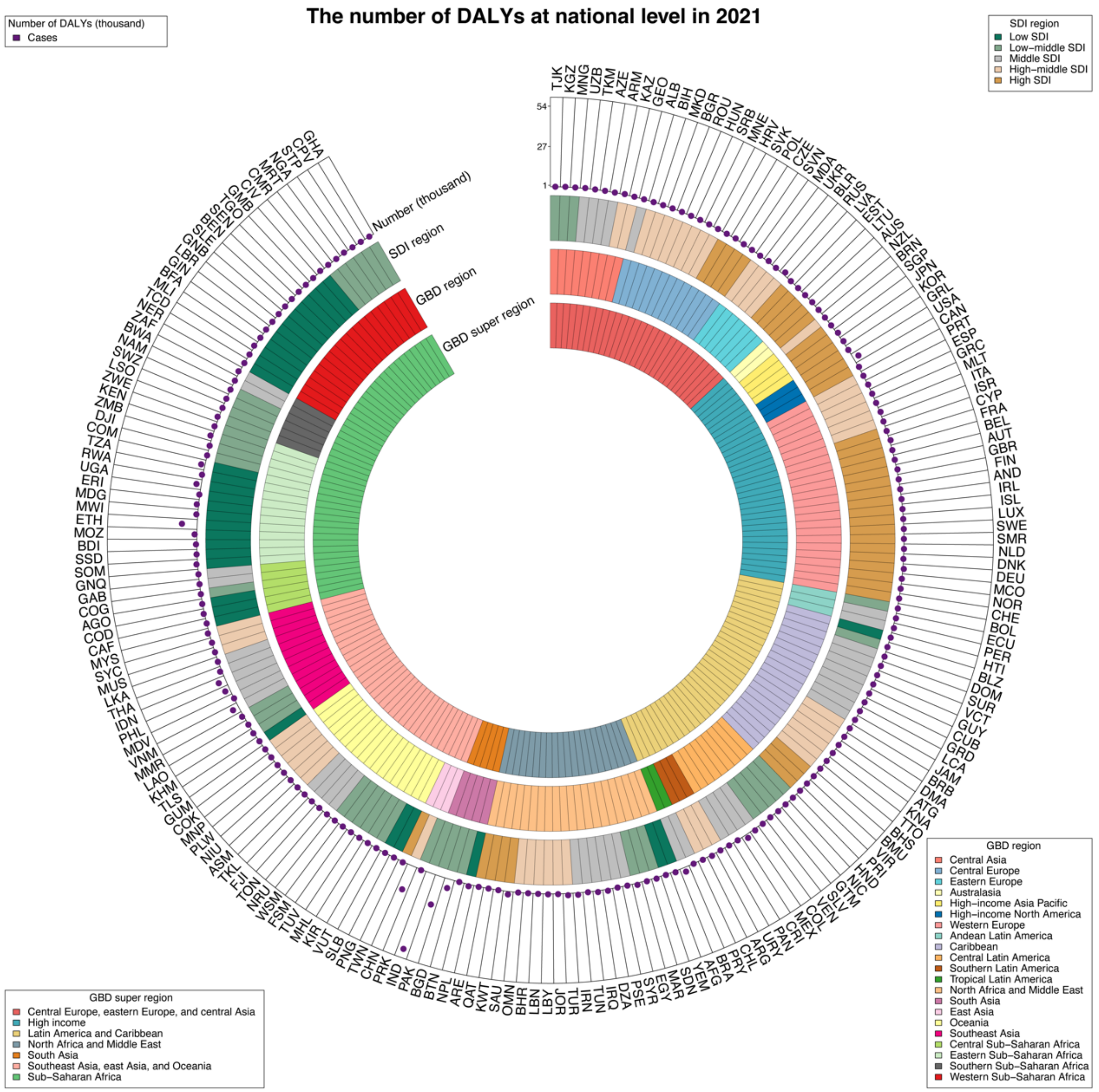

Figure S9. Trends of incidence, prevalence and DALYs for thyroid cancer among adolescents and young adults from 1990 to 2021. (A) Trends in incident cases and age special incidence rate. (B) Trends in death cases and age special death rate. (C) Trends in DALYs cases and age special DALYs rate. DALYs=disability-adjusted life-years.

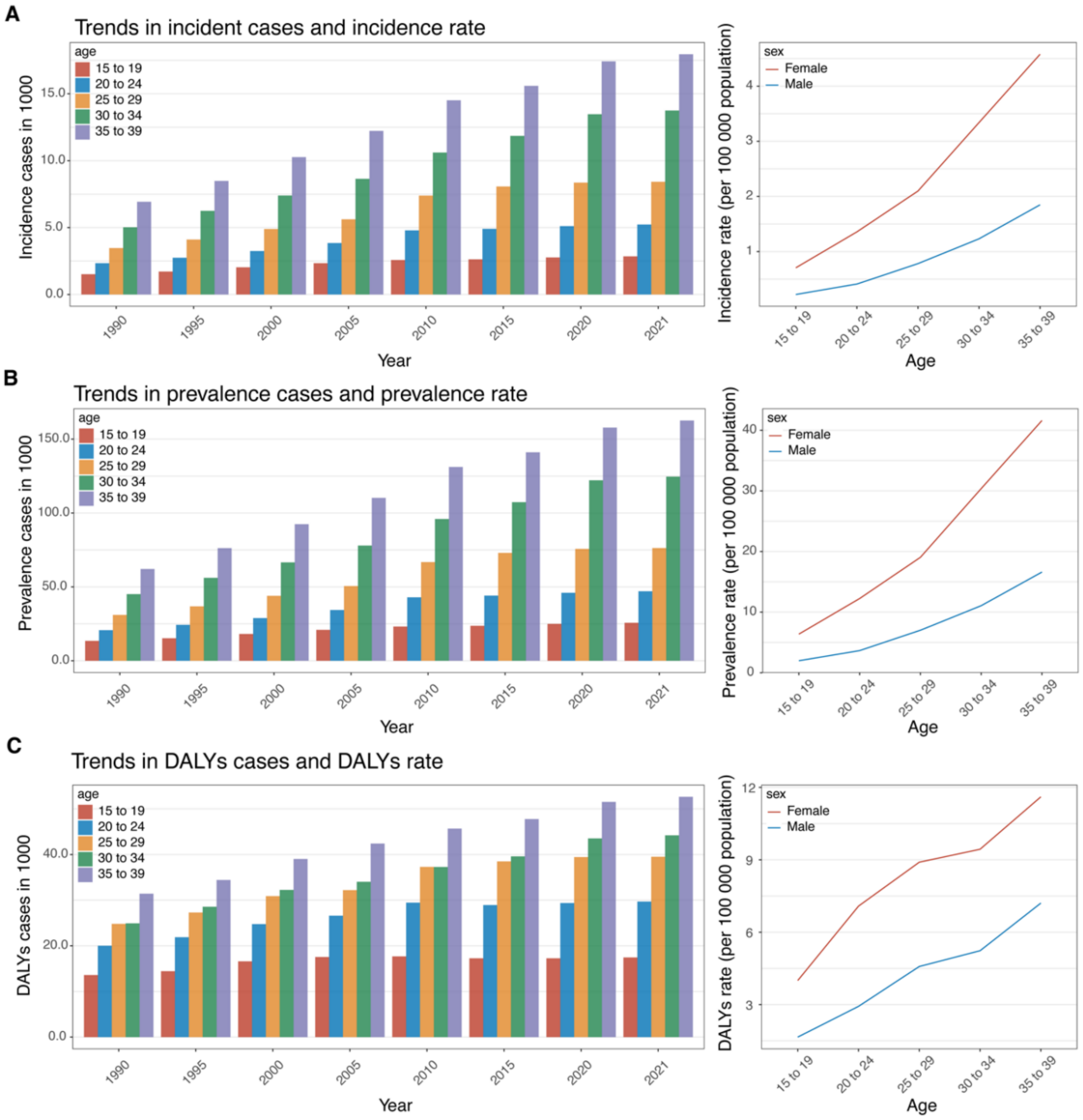

Figure S10: The results of Joinpoint regression analysis in five age groups at the global level from 1990 to 2021. (A) Incidence cases; (B) Prevalence cases; (C) DALYs cases. DALYs=disability-adjusted life-years.

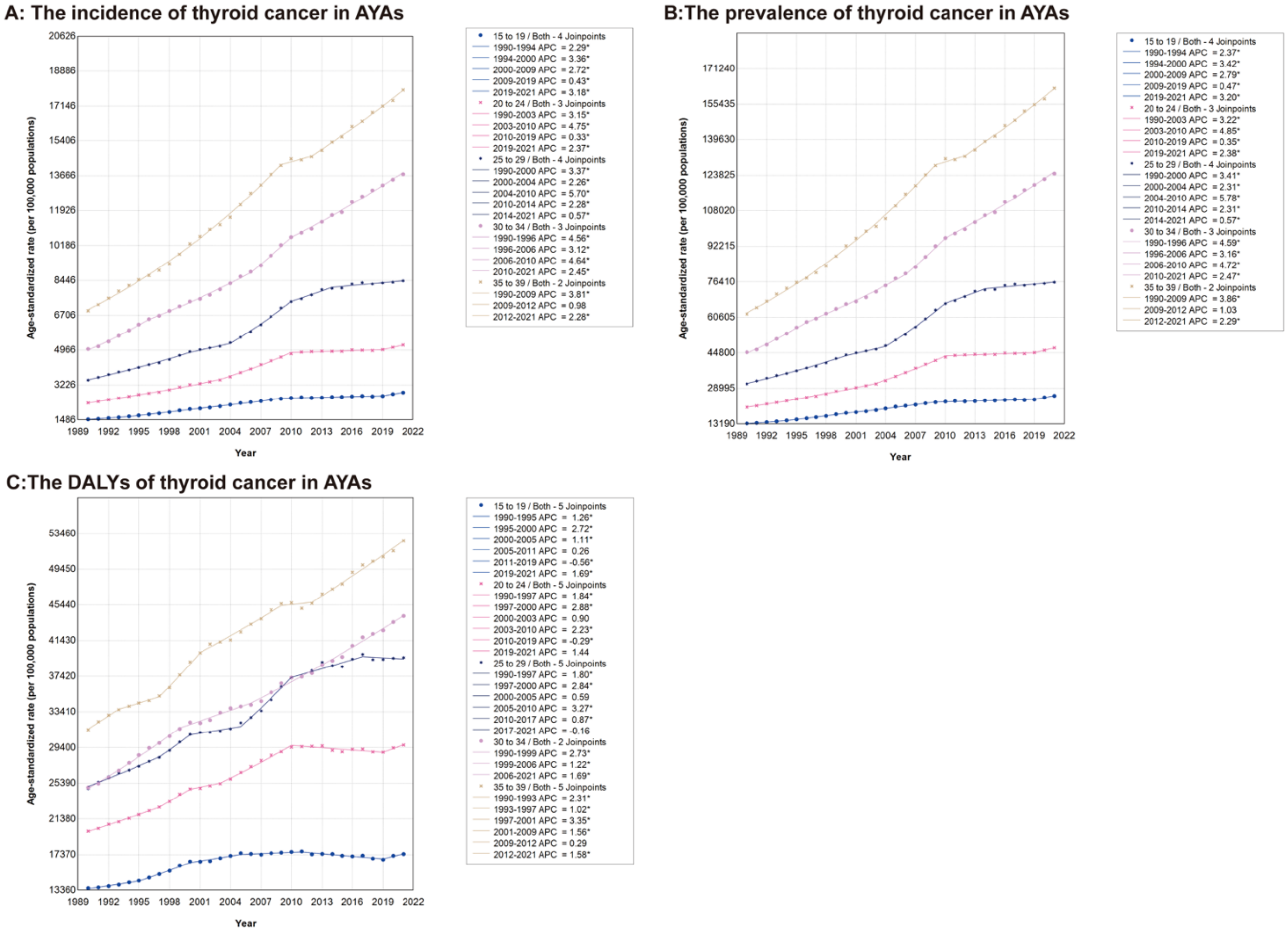

Figure S11. The trends of numbers in incidence (A), prevalence (B), and DALYs (C) for thyroid cancer among adolescents and young adults by sex, from 1990 to 2021. DALYs=disability-adjusted life-years.

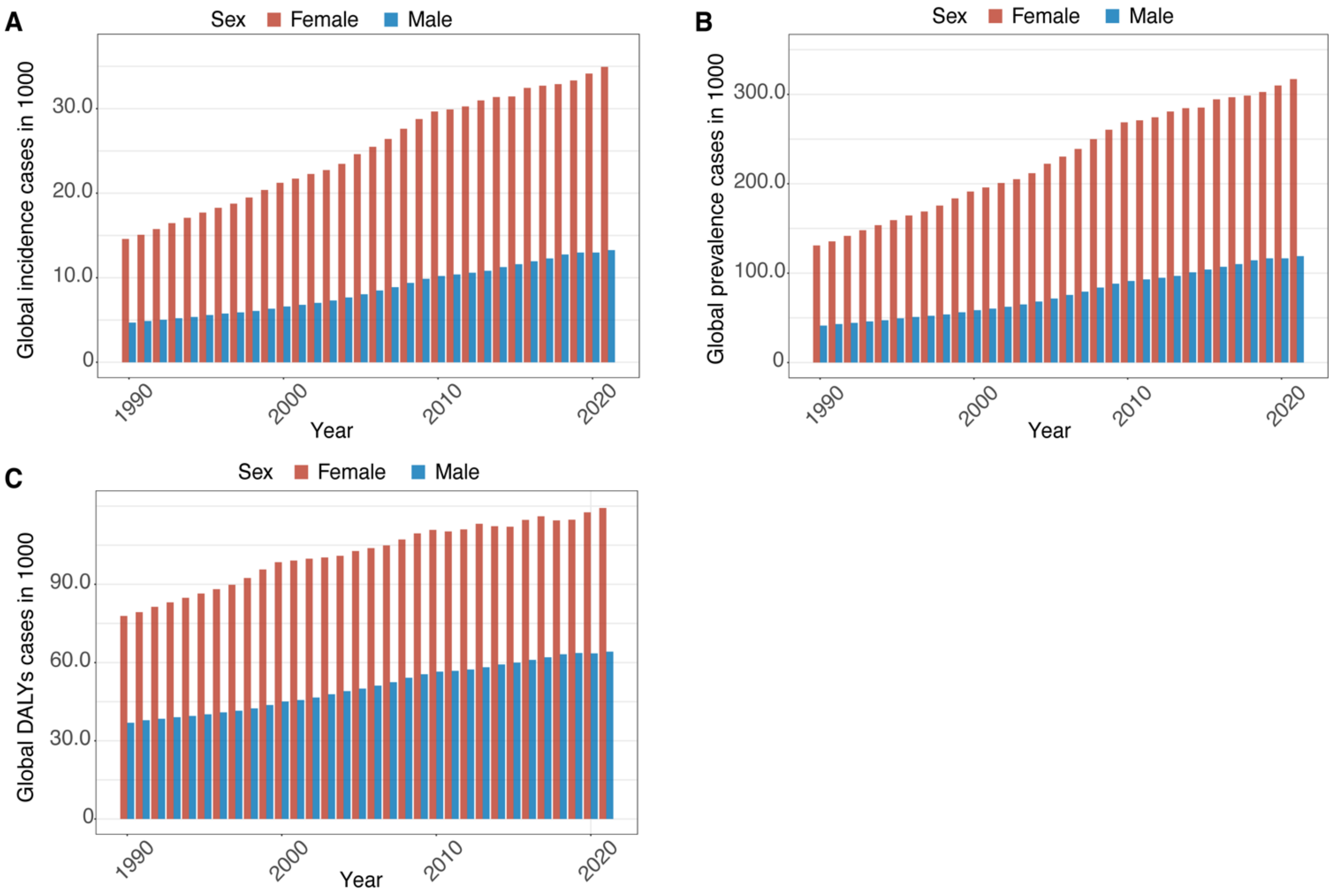

Supplement: S1 File — S1 Table. The Guidelines for Accurate and Transparent Health Assessment Reporting (GATHER). S2 Table. The Socio-demographic Index (SDI) reference values from the Global Burden of Disease (GBD) data released by the Institute for Health Metrics and Evaluation (IHME) in 2021. S3 Table. The Socio-demographic Index (SDI) values of 204 countries/territories from the Global Burden of Disease (GBD) data released by the Institute for Health Metrics and Evaluation (IHME) in 2021. S4 Table. The correspondence of Global Burden of Disease (GBD) regions with 204 countries/territories from the GBD data released by the Institute for Health Metrics and Evaluation (IHME) in 2021. S5 Table. Incidence of thyroid cancer in adolescents and young adults (AYAs), and their average annual percentage changes in 204 countries/territories from 1990 to 2021. S6 Table. Prevalence of thyroid cancer in adolescents and young adults (AYAs), and their average annual percentage changes in 204 countries/territories from 1990 to 2021. S7 Table. DALYs of thyroid cancer in adolescents and young adults (AYAs), and their average annual percentage changes in 204 countries/territories from 1990 to 2021. DALY = disability-adjusted life-year. S8 Table. The results of frontier analysis based on SDI and age-standardized DALYs rate for thyroid cancer in adolescents and young adults from 1990 to 2021. S9 Table. Incidence of thyroid cancer in adolescents and young adults (AYAs), and their average annual percentage changes from 1990 to 2021 by age at the global level. S10 Table. Prevalence of thyroid cancer in adolescents and young adults (AYAs), and their average annual percentage changes from 1990 to 2021 by age at the global level. S11 Table. DALYs of thyroid cancer in adolescents and young adults (AYAs), and their average annual percentage changes from 1990 to 2021 by age at the global level. DALY = disability-adjusted life-year. S12 Table. Decomposition of the percentage changes in thyroid cancer among adolescent [file pone.0318605.s001.pdf]
